# Integrating genomic structural equation modeling and experimental validation to unravel the genetic basis of male genital lichen sclerosus

**DOI:** 10.3389/fimmu.2026.1857945

**Published:** 2026-07-16

**Authors:** Jianbai Chen, Zhiming Zhang, Qiang Fu, Haozhong Hou, Gongquan Xu, Zhenyu Li, Zhiguo Lu, Jianxin Qiu, Ke Wu, Xiaoping Gao, Geng Zhang, Longfei Yang, Rundong Song, Wei Zhang

**Affiliations:** 1 Tangdu Hospital, The Fourth Military Medical University, Department of Urology, Xi ‘an, Shaanxi, China; 2 Xi’an People’s Hospital (Xi’an Fourth Hospital), Department of Ophthalmology, Xi ‘an, Shaanxi, China; 3 Tangdu Hospital, The Fourth Military Medical University, Department of Transfusion Medicine, Xi ‘an, Shaanxi, China; 4 The Second Affiliated Hospital of Xi’an Jiaotong University, Department of Urology, Xi ‘an, Shaanxi, China

**Keywords:** experimental verification, genome-wide association study, genomic structural equation modeling, male genital lichen sclerosus, single-cell sequencing analysis

## Abstract

**Objective:**

To investigate the genetic architecture of male genital lichen sclerosus (MGLSc) and to identify potential susceptibility loci, candidate genes, and biological pathways associated with disease pathogenesis by integrating genomic structural equation modeling (Genomic-SEM) with multi-omics analyses and experimental validation.

**Methods:**

Publicly available genome-wide association study (GWAS) summary statistics of MGLSc-related traits were integrated to construct a Genomic-SEM framework. Linkage disequilibrium score regression (LDSC) was used to estimate genetic correlations and evaluate model stability. Functional mapping and annotation were performed using FUMA, and novel loci were further screened through a GWAS subtraction strategy. Fine-mapping was conducted using SuSIE and FINEMAP to prioritize candidate causal variants. Transcriptome-wide association study (TWAS) and FOCUS were applied to identify candidate genes. MAGMA-based gene enrichment, partitioned heritability analysis, and polygenic risk score (PRS) analyses were further performed to characterize the biological relevance of associated loci. Finally, RT-qPCR was conducted *in vitro* to validate the expression of prioritized genes.

**Results:**

The Genomic-SEM showed a good overall fit and generated an indirect GWAS framework comprising 2,451,318 SNPs for MGLSc. A total of 208 SNPs reached conventional genome-wide significance, and FUMA annotation identified 43 risk loci, 52 lead SNPs, and 14 candidate genes. Using the GWAS subtraction strategy, 13 novel SNPs were further identified, including rs715299 and rs10774625. Fine-mapping highlighted four high-confidence variants, namely rs3134608, rs3134952, rs3763307, and rs2076524, mainly clustered in the chromosome 6 major histocompatibility complex region. TWAS identified HLA-DPA1 as the most significant gene, and FOCUS further supported its likely causal role. MAGMA and enrichment analyses suggested that immune-related and regulatory regions contributed substantially to MGLSc heritability. PRS analysis demonstrated marked heterogeneity across chromosomes, with chromosome 6 showing the strongest SNP-level contribution. RT-qPCR confirmed that HLA-DPA1 expression was significantly decreased in MGLSc samples compared with controls (*P* < 0.0001), consistent with the bioinformatics prediction. In addition, several MAGMA-prioritized genes, including C4B, DDR1, BBS7, VARS1, PRRT1, PPT2, EGFL8, BTNL2, and EME1, were downregulated, whereas AGPAT1 and PBX2 were upregulated in MGLSc samples.

**Conclusion:**

This study provides a systematic view of the genetic basis of MGLSc by integrating Genomic-SEM, fine-mapping, transcriptomic prioritization, and experimental validation. Our findings indicate that MGLSc is influenced by a shared polygenic architecture enriched in immune-related loci, particularly within the HLA region. HLA-DPA1 emerged as a high-confidence susceptibility gene, and multiple novel loci and candidate genes were identified, offering new insights into the molecular mechanisms underlying MGLSc and potential targets for future mechanistic and translational studies.

## 1 Introduction

Male genital lichen sclerosus (MGLSc) is a chronic lichenoid inflammatory fibrosing disorder primarily diagnosed based on clinical symptoms, which include male sexual dysfunction, pruritus, and cutaneous lesions (1, 2). The etiology of MGLSc remains uncertain, with several hypotheses suggesting autoimmune mechanisms, immune dysregulation, and infectious factors (1, 3, 4). Previous studies have highlighted the important role of the foreskin in the development of MGLSc, as the condition is exceedingly rare in men who have undergone circumcision at birth (2, 5). Another hypothesis posits that MGLSc may result from susceptible epithelial cells being exposed to urine, however, the specific components or characteristics of urine responsible, as well as the relevant susceptibility factors, remain unidentified (5, 6). As a rare disease, current researches on MGLSc primarily focus on clinical studies and case reports. Unfortunately, our understanding of the underlying basic research on MGLSc remains nearly blank, especially regarding the exploration of its genetic and biological mechanisms. To address the current lack of precise measurement of MGLSc mechanisms, we designed a genome-wide association study (GWAS) targeting potentially unmeasured MGLSc.

In this study, we employed Genomic Structural Equation Modeling (Genomic-SEM), a method that leverages publicly available GWAS summary statistics from MGLSc-associated diseases and biomarkers. Using these statistics, we estimated associations between single-nucleotide polymorphisms (SNPs) and the phenotype of MGLSc, thereby constructing a novel GWAS for unmeasured MGLSc and developing a unique Genomic-SEM framework for MGLSc. Additionally, drawing on integrative analysis methods from systems biology, we defined the portion of MGLSc genetic variation unexplained by known biomarkers as “potential relevant genetic markers” and conducted multiple GWAS-related investigations on these markers. This analytical framework has been validated in prior studies, including investigations of allergic diseases (7) and inflammatory bowel disease (8, 9).

Although this method does not perfectly capture the true relationships between MGLSc-related pathways and multi-factorial interactions—given that MGLSc is inherently a complex process driven by genetic, environmental, and stochastic factors—our study simulates MGLSc construction using Genomic-SEM to bypass these limitations. Importantly, this approach excludes known confounding effects on MGLSc-related biomarkers, enabling the analysis of previously challenging data. Collectively, our study seeks to establish a straightforward bridge between genomic statistics, basic research, and clinical practice.

To conclude, we have explored the genetic and biological mechanisms of MGLSc from a novel angle, thereby deepening our understanding of its underlying genetic and biological underpinnings.

## 2 Methods

### 2.1 Genomic-SEM GWAS data source

**Figure 1:**
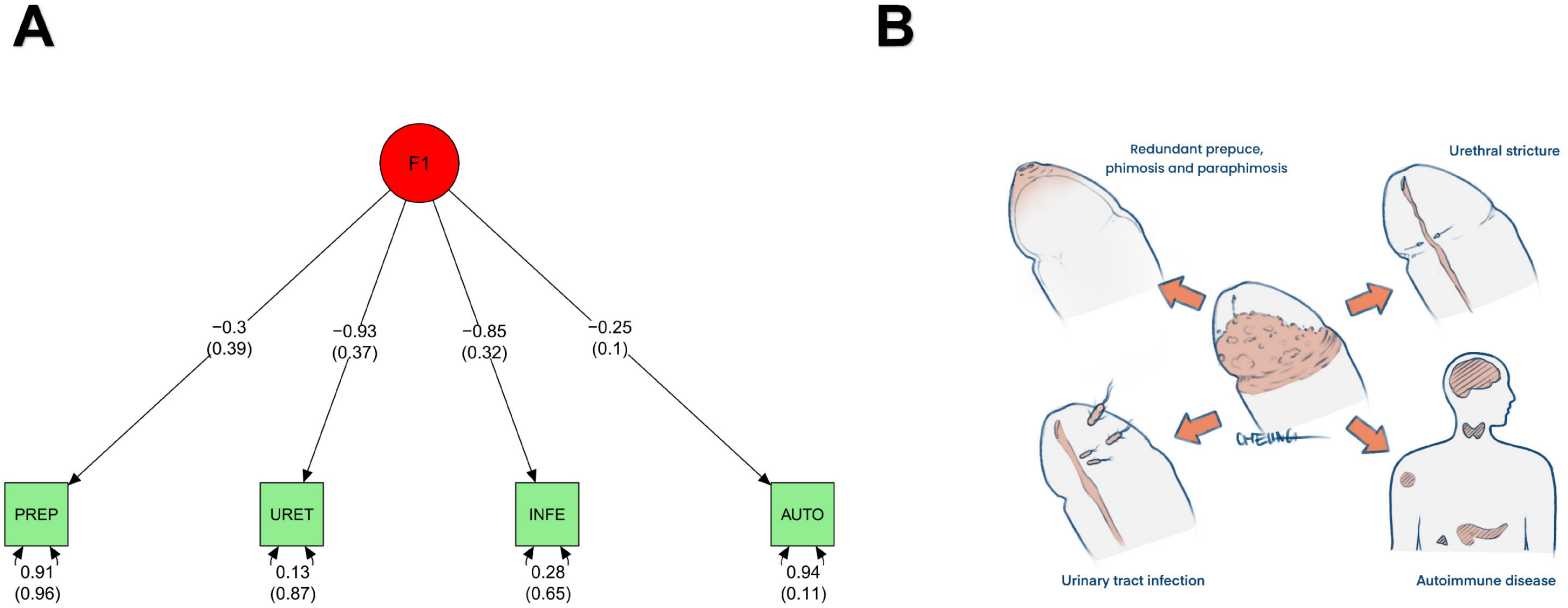
Genetic multivariate genetic factor model and multivariate GWAS of MGLSc. **(A)** Path diagram with standardized factor loadings in the hierarchical model estimated using Genomic-SEM. Red circles denote the shared genetic liability (F1) and green squares denote the observed univariate phenotypes. Single-headed arrows indicate factor loadings (standardized regression coefficients), and double-headed curved arrows indicate residual variances. (PERP, Redundant prepuce, phimosis and paraphimosis; URET, Urethral stricture; INFE, Urinary tract infection; AUTO, Autoimmune diseases) **(B)** Components in the Genomic-SEM for MGLSc (The illustrations were expertly crafted by Professor Zhiming Zhang, a skilled medical illustrator).

We selected MGLSc-associated diseases and phenotypes based on clinical comorbidity, pathophysiological relevance and genetic evidence, including redundant prepuce, phimosis and paraphimosis, urethral stricture, urinary tract infection and autoimmune diseases. The GWAS summary statistics used for Genomic-SEM analysis in this study were derived from four independent GWAS datasets associated with MGLSc, which were previously published in the UK Biobank and FinnGen R12 databases. The endpoints of autoimmune diseases included autoimmune diseases such as Ulcerate colitis, Type1 diabetes, Psoriasis, Crohn disease, Primary biliary cholangitis, Obesity, Dementia etc. We confirm that MGLSc and related genital inflammatory skin diseases are explicitly excluded from the autoimmune diseases definition (Detailed results for the diseases included in autoimmune diseases are available in https://r9.risteys.finngen.fi/endpoints/AUTOIMMUNE#). All input GWAS datasets obtained ethical approval from their respective institutional review boards (IRBs), with informed consent provided by all participants. Data quality control was rigorously applied to ensure data integrity, and detailed information on the GWAS datasets included in this analysis is provided in **Table 1**. The schematic diagram of Genomic-SEM is shown in **Figure 1**.

**Table 1:** GWAS summary statistics sources.

Trait	Role in current study	GWAS id	Source	Population	Sex	Genome build	Download link	N case	N control	Sample size	Number of SNPs
Redundant prepuce, phimosis and paraphimosis	Genomic structure equation model	ukb-b-10668	UK Biobank	European	Males and Females	HG19/GRCh37	https://gwas.mrcieu.ac.uk/files/ukb-b-10668/ukb-b-10668.vcf.gz	1,541	461,469	463,010	9,851,867
Urethral stricture	Genomic structure equation model	ukb-b-9074	UK Biobank	European	Males and Females	HG19/GRCh37	https://gwas.mrcieu.ac.uk/files/ukb-b-9074/ukb-b-9074.vcf.gz	2,013	460,997	463,010	9,851,867
Urinary tract infection	Genomic structure equation model	ukb-b-8814	UK Biobank	European	Males and Females	HG19/GRCh37	https://gwas.mrcieu.ac.uk/files/ukb-b-8814/ukb-b-8814.vcf.gz	5,447	457,563	463,010	9,851,867
Autoimmune diseases	Genomic structure equation model	finn-b-AUTOIMMUNE	Finn 12 database	European	Males and Females	HG19/GRCh37	https://storage.googleapis.com/finngen-public-data-r12/summary_stats/release/finngen_R12_AUTOIMMUNE.gz	128,075	372,273	500,348	NA

### 2.2 Quality control for genomic-SEM

In this study, rigorous quality control (QC) was applied to the GWAS datasets included in the analysis. All summary datasets were harmonized to GRCh37/hg19 genome build. To maintain result consistency, samples with a missing rate exceeding 5% were excluded. Prior to constructing the Genomic-SEM, we first used GWAS datasets filtered by recommended default QC criteria. Subsequently, additional strict QC was performed on all autosomal SNPs across the four GWAS datasets relevant to the Genomic-SEM construction, including: (1) removing SNPs with a minor allele frequency (MAF) < 0.01 (these SNPs are prone to errors due to smaller sample sizes within genotype clusters and typically exhibit higher linkage disequilibrium (LD) score regression standard errors); (2) excluding SNPs with zero effect estimates (to avoid compromising matrix conditioning, which is critical for Genomic-SEM); (3) removing SNPs mismatched with the reference panel; and (4) excluding SNPs with mismatched alleles.

### 2.3 Construction of genomic-SEM and sample overlap in genomic-SEM

The GWAS datasets included in our analysis originated from distinct genome-wide data repositories. Although the summary statistics were derived from large-scale biobank data, we acknowledged the potential for participant overlap, particularly among traits sourced from the UK Biobank. To address this, we employed the Genomic-SEM which explicitly models and corrects for sampling covariance arising from overlapping samples. Consequently, potential sample overlaps between cohorts were explicitly accounted for during the GWAS analysis to ensure the accuracy and generalizability of results, while also addressing the statistical implications of overlapping samples.

The construction of Genomic-SEM was performed using the GenomicSEM R package (v0.0.5) to analyze the four publicly available MGLSc-related GWAS summary statistics, aiming to investigate the broad genetic susceptibility underlying these MGLSc-related traits. As a newly developed method, Genomic-SEM enables the investigation of multiple multivariate models to explore the underlying structure of traits of interest. Notably, Genomic-SEM is not susceptible to biases arising from sample overlap (e.g., UKB participants overlapping across multiple input GWAS in validation) or sample size imbalance. Additionally, it facilitates the identification of variants that influence only a subset of—rather than all—complex traits, which therefore do not represent broad cross-trait susceptibility.

The analysis for Genomic-SEM was conducted in two stages. In the first stage, we estimated the empirical genetic covariance matrix and the corresponding sampling covariance matrix. Summary statistics from MGLSc-associated GWAS were prepared, and the empirical genetic covariance matrix for the four traits was generated using the multivariate extension of cross-trait LD score regression, serving as input for the common factor model. In the second stage, a structural equation model (SEM) was constructed to minimize the discrepancy between the hypothesized covariance matrix and the empirically estimated covariance matrix from the first stage. Here, our primary objective was to characterize the genetic architecture underlying the four MGLSc-related traits, prompting us to test a single-factor model. Model fit was evaluated using standardized metrics, including the standardized root mean square residual (SRMR), chi-squared statistic (χ²), Akaike information criterion (AIC), and comparative fit index (CFI). By applying appropriate common factor Structural Equation Modeling (SEM) specifications, individual autosomal SNP associations were integrated into the genetic and sample covariance matrices, yielding polygenic genome-wide association results for shared covariance across MGLSc-associated GWAS.

### 2.4 SNP heterogeneity in genomic-SEM

To evaluate whether SNP associations in MGLSc-related traits were appropriately modeled within the multivariate SEM, we computed SNP heterogeneity statistics (Q-test). The null hypothesis for the Q-test posits that the genetic effect of a given SNP on the latent MGLSc phenotype is consistent across all input GWAS traits, and that the multivariate SEM model fully accounts for the SNP’ s association, leaving no residual heterogeneity. SNPs with Q-statistic *P* < 0.05 were excluded from further analysis, as they indicated unexplained heterogeneity or model misspecification.

### 2.5 Multilevel evaluation of genomic-SEM

To assess the stability of the constructed Genomic-SEM, we implemented a multilevel strategy for model adjustment. First, we applied a two-stage association testing threshold approach, using both a strict threshold (*P* < 5 × 10^−16^) and a relatively relaxed threshold (*P* < 5 × 10^−12^) to identify novel SNP loci detectable at different analytical depths. Subsequently, we refined the model using a two-step LD Score regression-based genomic control procedure, which involved: (1) removing SNPs with missing values; (2) removing SNPs with an INFO score < 0.9; (3) removing SNPs with a MAF < 0.01; (4) removing SNPs with P values outside the valid range; and (5) removing SNPs with ambiguous strand orientation. LD score partitions with zero variance were removed to ensure data quality. We further applied a two-step estimator with an LD decay cutoff of 30 cM to balance the effects of population structure and the resolution of genetic association signals.

### 2.6 Functional mapping and annotation of genomic-SEM

We applied the “Functional Mapping and Annotation of Genetic Associations” workflow in FUMA (Functional Mapping and Annotation, https://fuma.ctglab.nl/) to identify genome-wide loci associated with MGLSc and potential related SNPs—defined as SNPs in low linkage disequilibrium (LD < 0.1) with other variants and reaching genome-wide significance (*P* < 5 × 10^−8^). First, we input the summary statistics of SNPs from our constructed SEM to evaluate their association strengths. Additionally, we compared the potential related SNP loci identified by the SEM with those from the original single-input GWAS to assess their overlap.

To further validate potential pleiotropic associations of the SEM-derived related SNPs, we cross-referenced significant SNP findings (*P* < 5 × 10^−8^) from the GWAS Catalog, a public repository of genome-wide association results. We also leveraged the functional annotation module of FUMA to perform gene-level locus analysis for the SEM, applying a significance threshold of *P* < 5 × 10^−8^. Output files from FUMA were processed using MAGMA (Multi-marker Analysis of Genomic Annotation), a tool designed for gene-level association analysis and functional annotation integration (10). MAGMA aggregates multiple genetic markers into gene-level signals, calculates gene-trait associations, and applies false discovery rate (FDR) correction (FDR-adjusted *P* < 0.05). This approach extracts functionally relevant information from genome-wide SNP data to uncover gene-level genetic signals and reveal the biological mechanisms underlying complex traits or diseases.

Furthermore, we employed a “GWAS subtraction” strategy to refine lead SNP discovery. This method compares the SNPs identified by the SEM with those reaching genome-wide significance in single-input GWAS, subtracting previously reported loci to prioritize novel, biologically meaningful variants specific to the SEM.

### 2.7 Combined SuSIE and fine-mapping analysis

To identify the most likely causal variants associated with our Genomic-SEM, we applied two fine-mapping tools—Sum of Single Effects (SuSIE) and Fine-Mapping (FINEMAP) (11, 12)—implemented via the R package echolocatoR v.2.0.3. A posterior probability (PP) threshold of 0.95 was set to define a credible set of putative causal variants. The workflow is detailed as follows:

Both SuSIE and FINEMAP are fine-mapping tools designed to pinpoint the most probable causal variants linked to a phenotype. For this analysis, we defined a 250 kb window around each lead SNP to capture the genomic region associated with it, within which we calculated the causal inference probability for each SNP. A PP threshold of 0.95 was applied: variants with PP exceeding this threshold were considered putative causal variants.

Using echolocatoR, we further defined “consensus SNPs”—variants that appeared in both SuSIE and FINEMAP results. For these consensus SNPs, echolocatoR computes their average posterior probability and determines an average credible set: when the PP of a SNP exceeds 0.95 in both SuSIE and FINEMAP, its credibility is assigned a value of 1, otherwise, it is set to 0.

### 2.8 Transcriptome-wide association study

Following the identification of putative causal variants, we performed a Transcriptome-Wide Association Study (TWAS) to prioritize MGLSc-associated genes based on the relationship between gene expression and phenotype (13). We applied the FUSION method for TWAS. FUSION is a suite of tools for performing TWAS. FUSION builds predictive models of the genetic component of a functional/molecular phenotype and predicts and tests that component for association with disease using GWAS summary statistics. The goal is to identify associations between a GWAS phenotype and a functional phenotype that was only measured in reference data. We utilized 37,920 precomputed expression quantitative trait loci (eQTL) features (gene-tissue pairs) from the GTExv.8 dataset. These features quantify expression associations between genes and tissues across diverse biological contexts.

Subsequently, we filtered genes using FDR correction, retaining those with TWAS *P* < 0.05 that were also significantly associated with our constructed structural equation model. These prioritized genes were then subjected to further analysis using the Fine-mapping Of Causal gene Sets (FOCUS) (14) method, a TWAS-specific fine-mapping approach designed to evaluate gene-phenotype causal relationships via FOCUS posterior inclusion probability (PIP). Genes with a PIP threshold > 0.8 were considered to have strong causal evidence.

Consistent with prior studies, we focused on TWAS-significant genes that not only demonstrated statistical significance in the TWAS analysis but also aligned with FOCUS-derived conclusions, indicating their potential causal role in MGLSc.

### 2.9 Gene set and disease ontology enrichment analysis

We performed gene enrichment analysis and gene pathway set analysis using the Multi-marker Analysis of GenoMic Annotation (MAGMA) (10) and Functional Mapping and Annotation (FUMA) tools on Gene Set Enrichment Analysis (GSEA) datasets to investigate the potential associations between MGLSc and Mendelian disease genes, as well as their related pathways. Additionally, gene enrichment analysis was conducted using the MendelVar platform (https://mendelvar.mrcieu.ac.uk/submit/) (15).

### 2.10 Cell annotation analysis and regional heritability contribution analysis

To identify etiological cell types associated with MGLSc, we applied CELL-type Expression-specific integration for Complex Traits (CELLECT) (16)—a method designed for single-cell RNA sequencing (scRNA-seq) data of complex traits—using data from the Tabula Muris90 dataset (17). This database contains transcriptomic data from 100,000 cells across 20 organs and tissues of the Mus musculus. We preprocessed and normalized the Tabula Muris scRNA-seq data using CELL-type Expression-specificity (CELLEX) (16), a tool that calculates expression specificity probability scores for each gene. Subsequently, we performed cell-type-specific analysis using the Linkage Disequilibrium Score Regression (LDSC) tool (18), classified cell types, and applied a FDR threshold of 0.05.

LDSC enables the calculation of partitioned heritability, which quantifies the contribution of distinct genome regions (e.g., genes, enhancers, silencers) to trait heritability by partitioning the genetic information of a phenotype across these regions (18–20). Specifically, LDSC utilizes a weighted LD matrix, genotype frequency files, and summary statistics to estimate the genetic contribution of each genomic region (19).

### 2.11 Construction of polygenic risk scores using summary data​

We computed polygenic risk scores (PRS) based on genome-wide summary statistics and evaluated the genetic contributions of distinct chromosomal regions to the disease’s etiology. Specifically, we applied the Polygenic Risk Score with Continuous Shrinkage (PRS-CS) method, which uses genome-wide association study GWAS data and external LD reference panels to estimate the posterior effect values of SNPs. This method employs a Bayesian regression that integrates GWAS summary statistics with LD reference panels to estimate effect values, ultimately enabling the calculation of PRS.

### 2.12 Sensitivity analysis on the impact of autoimmune genetic background

To investigate whether the MHC/HLA association is driven solely by broad autoimmune signals, we performed a leave-one-out Genomic-SEM analysis. The model was reconstructed using only the three urogenital traits (Redundant prepuce, phimosis and paraphimosis, Urethral stricture and Urinary tract infection), excluding the autoimmune disease GWAS datasets. Model fit indices were compared between this restricted model and the full model (including all four traits). Subsequently, we performed FUMA and TWAS study to assess the impact of removing the autoimmune genetic component.

### 2.13 Prognostic gene expression and quantitative reverse transcription polymerase chain reaction

To elucidate the expression characteristics of prognostic genes, the Wilcoxon signed-rank test was employed to compare differences in prognostic gene expression between MGLSc samples and control samples (with a significance threshold set at *P* < 0.05). The accuracy of the above results was subsequently validated by RT-qPCR. A case sample and control sample were collected at Tangdu Hospital. All participants were required to sign informed consent forms. Ethical approval was granted by the Clinical Ethics Committee of Tangdu Hospital, Air Force Medical University (Approval No: TDLL-KY-20230104). Total RNA was extracted from one pair of samples using TRizol reagent (NuoWeiZan, Nanjing, Jiangsu). RNA concentration was measured using the NanoPhotometer N50. Subsequently, mRNA was reverse transcribed into cDNA using the Hifair^®^ III First Strand cDNA Synthesis Premix Kit (Yisheng Bio, Shanghai) for qPCR detection. Reaction reagents, conditions, and gene primer information are detailed in **Supplementary File 1**. Expression levels of prognostic genes were normalized using the housekeeping gene GAPDH and calculated via the 2^(-ΔΔCt) method. Data were analyzed using GraphPad Prism software (v 5.0).

## 3 Results

### 3.1 Genomic-SEM construction

**Figure 2:**
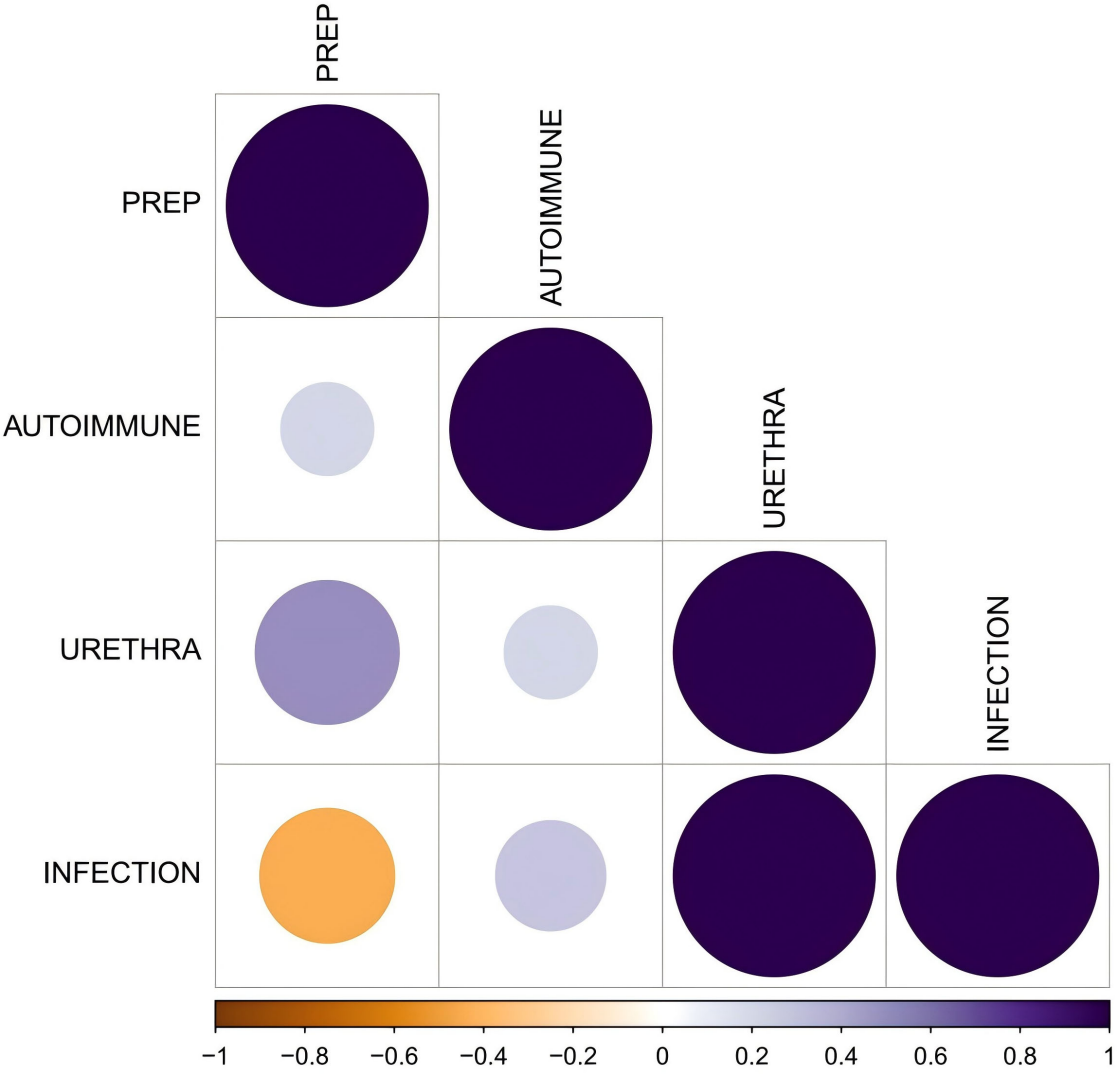
MGLSc genetic correlation matrix. The color intensity and the size of the circles are proportional to the strength of the correlation.

The LD Score regression analysis revealed that the heritability contributions of the four univariate input GWAS variables constituting the MGLSc structural equation—redundant prepuce, phimosis and paraphimosis, urethral stricture, urinary tract infection, and autoimmune diseases—are 0.0012, 0.0016, 0.0017, and 0.0516, respectively. This indicates that autoimmune diseases exhibit the highest genetic contribution within the model, demonstrating significant heritability, while the heritability of traits related to the prepuce is comparatively weaker. Detailed univariate heritability parameters can be found in **Supplementary Table 1**, and the genetic interactions between traits are depicted in **Figure 2**, with covariance values provided in **Table 2**.

**Table 2:** Genetic correlations among the four univariate traits.

Phenotype 1	Phenotype 2	Rg (se)	*P* value
Redundant prepuce, phimosis and paraphimosis	Urethral stricture	0.5082 (0.5725)	0.37469
Redundant prepuce, phimosis and paraphimosis	Urinary tract infection	-0.4461 (0.632)	0.48029
Redundant prepuce, phimosis and paraphimosis	Autoimmune diseases	0.2116 (0.1579)	0.18034
Urethral stricture	Urinary tract infection	1.526 (0.5087)	0.0027024
Urethral stricture	Autoimmune diseases	0.2126 (0.1306)	0.10341
Urinary tract infection	Autoimmune diseases	0.298 (0.1164)	0.010472

Genetic correlations were considered statistically significant at *p* < 0.05.

Rg (se), Genetic correlation coefficient (standard error).

In preparing for structural equation modeling, we conducted a structural equation model analysis. Specifically, the model fit indices showed a CFI of 1.0 and a SRMR of 0.144, indicating that the genetic covariance matrix derived from the four univariate input GWAS fits well with the empirical covariance matrix of the shared factor model (shown in **Table 3** for details). The results for the latent factor loadings and residual covariances of the structural equation model are presented in **Supplementary Table 2**. These findings suggest the presence of shared genetic factors in our constructed structural equation model. The final Genomic-SEM analysis produced an indirect measurement-based GWAS comprising 2,451,318 SNPs to investigate the genetic architecture of MGLSc.

**Table 3:** Fit indices for genomic-SEM model.

chisq	df	*P* chisq	AIC	CFI	SRMR
1.99644090497656	2	0.368534682959621	17.9964409049766	1	0.143971070617284

chisq, Chi-Square Test Statistic; df, Degrees of Freedom; p chisq, p-value for the Chi-Square Test; AIC, Akaike Information Criterion; CFI, Comparative Fit Index; SRMR, Standardized Root Mean Square Residual.

### 3.2 Hierarchical evaluation of genomic-SEM​

By extending the SEM to incorporate individual-level genetic variation, we developed a GWAS to estimate associations between 2,451,318 SNPs and MGLSc. In this analysis, 208 SNP loci reached a conventional significance threshold of *P* < 5 × 10^−8^. Under the genome-wide significance threshold of *P* < 5 × 10^−12^, we identified 150 significant SNPs across the 2,451,318 loci, with 113 SNP loci further validated at a stricter threshold (*P* < 5 × 10^−16^) (shown in **Supplementary Table 3** for details). Notably, these loci differ from those identified by the four single-input GWAS datasets used to construct the Genomic-SEM, underscoring the enhanced detection power of Genomic-SEM.

### 3.3 Assessment the stability of genomic-SEM via LDSC regression

Through parameter-based filtering in our method, a total of 1,867,850 SNPs were removed. Following regression coefficient-based selection, 583,468 effective SNPs were retained. Key statistical metrics included a mean chi-squared value of 0.448 across all SNPs, a genomic control Lambda GC of 0.477, and a maximum chi-squared value of 913.763. At the genome-wide significance level, 21 loci were identified. Heterogeneity testing indicated no significant between-study heterogeneity (*P* > 0.05). The total observed-scale heritability (h²) was estimated to be 1.9088 × 10^−9^ (9.1556 × 10^−10^). Notably, the contribution ratio of genetic to environmental factors was < 1. The intercept term in the regression model was 0.4321 (standard error = 0.0051). Collectively, these results provide robust evidence that the observed inflation in the structural equation of interest arises from polygenic heritability signals, rather than confounding by population stratification bias or pleiotropic parameter effects.

### 3.4 Evaluation of genomic-SEM using FUMA software​

**Figure 3:**
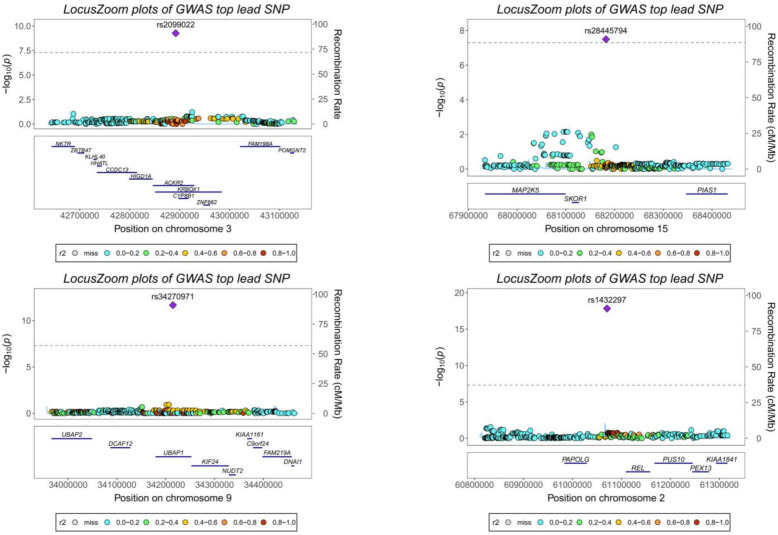
Visualization of risk loci annotation using FUMA for GWAS-identified SNPs.

Using FUMA software, we conducted genome-wide structural analysis and identified 43 risk genetic loci (**Figure 3**, **Supplementary File 2**). We further annotated all identified risk loci (shown in **Supplementary Table 4**). Furthermore, we identified 14 potential genes associated with MGLSc through genome-wide significant control, applying a significance threshold of 5 × 10^−8^ and a FDR of less than 0.05 (shown in **Supplementary Table 5**). By applying FUMA, we annotated 52 lead SNPs, the majority of which were located in intergenic regions (shown in **Supplementary Table 6**). Utilizing our custom “GWAS subtraction” method, we further identified 13 putative novel SNPs, including rs715299 and rs10774625 (shown in **Supplementary Table 7**).

Further analysis of the literature and the annotation results from this study reveals that some novel SNPs have relatively clear phenotypic associations. rs3131084 has been reported multiple times in the past as a breast cancer susceptibility locus. However, it also suggests that it may reflect a shared genetic background for broader complex traits. These loci likely represent additional layers of genetic susceptibility contributing to MGLSc, potentially through pleiotropic effects shared with immune or urinary traits. Although rs12131 has been reported in multiple studies, based on the GWAS subtraction results from this study, we are more inclined to believe that it is not a core locus directly driving the development of MGLSc, but may function as a potential mediator by influencing other intermediate phenotypes. In contrast, the phenotypic associations of rs568896 in previous studies are more consistent with the results of this study, suggesting that this locus may be more directly involved in MGLSc-related immune inflammation or tissue remodeling processes. Overall, these loci collectively support the notion that MGLSc is not driven by a single pathway but may involve the combined effects of immune abnormalities, chronic inflammation, and multifactorial genetic networks.

Among the remaining novel SNPs, rs715299 and rs10774625 have the strongest existing evidence linking them to MGLSc. Although rs715299 has been associated with cardiovascular disease, psychiatric disorders, and inflammatory skin diseases (21–24). However, the direction of the association in immune-mediated inflammatory diseases such as atopic dermatitis and psoriasis is more consistent with the phenotypes observed in this study (23, 24). rs10774625 exhibits a more pronounced multifactorial nature, having been previously associated not only with metabolic syndrome, dyslipidemia, and type 2 diabetes, but also with hematological traits (25–27). Moreover, it is associated with autoimmune diseases, including systemic lupus erythematosus, rheumatoid arthritis, and psoriasis, along with urinary biomarkers (28–35). This indicates that it may represent a critical intersection connecting immune imbalance, metabolic disorders, and abnormalities in the urinary system.

In addition, rs130065 and rs3099837 have been identified in previous studies as genetic loci associated with serum proteins (36), rs3095330, rs2621358, and rs13194770, among others, are more commonly found in studies related to mental health or other complex traits (37–42). Based on the findings of this study, we believe that these loci are more likely to participate in the pathogenesis and progression of MGLSc as potential mediating factors by influencing circulating protein levels, the state of immune inflammation, or other intermediate phenotypes, rather than serving as core genetic signals that directly drive the disease. These results further suggest that the genetic basis of MGLSc may not be determined by a single localized lesion, but rather is influenced by the combined effects of immune abnormalities, systemic inflammation, and multi-level biological networks. While the GWAS subtraction strategy is effective for identifying loci that do not reach genome-wide significance in univariate analyses, we acknowledge its limitation in regions of high LD, such as the MHC. In such regions, subtracting a single lead SNP does not eliminate the influence of extended haplotypes. Therefore, the findings within the MHC region ought to be construed as signifying independent associations within the broader autoimmune haplotype rather than necessarily indicating MGLSc-specific causality.

### 3.5 Finemap

**Figure 4:**
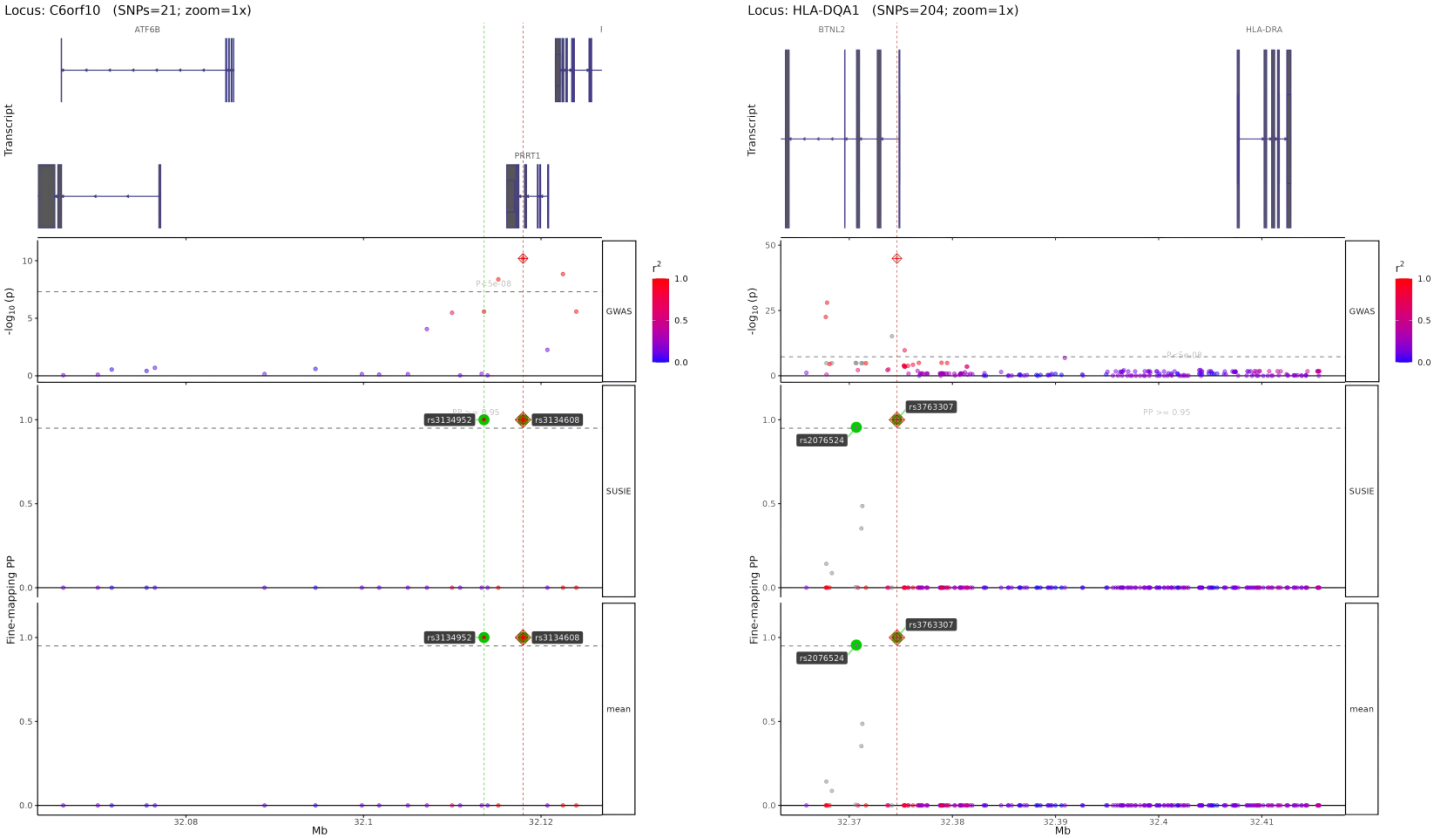
Genomic loci identified by SuSIE and FINEMAP (posterior probability > 0.95).

The results of Fine-mapping analysis identified strong genetic associations at multiple genomic loci, including: rs3134608 and rs3134952 on chromosome 6 (proximal to C6orf10), and rs3763307 and rs2076524 on chromosome 6 (proximal to HLA-DQA1). Corresponding to these fine-mapped loci, the *P* values were 6.40 × 10^−11^, 2.66 × 10^−06^, 1.21 × 10^−45^, and 1.56 × 10^−05^, respectively. Regional plots revealed distinct peaks at these loci, with additional evidence of association observed for other credible set variants (shown in **Figure 4**, **Supplementary Table 8**). Detailed fine-mapping results for all SNPs are provided in **Supplementary File 3**.

### 3.6 Integrative transcriptome-wide association study and colocalization analysis​

**Figure 5:**
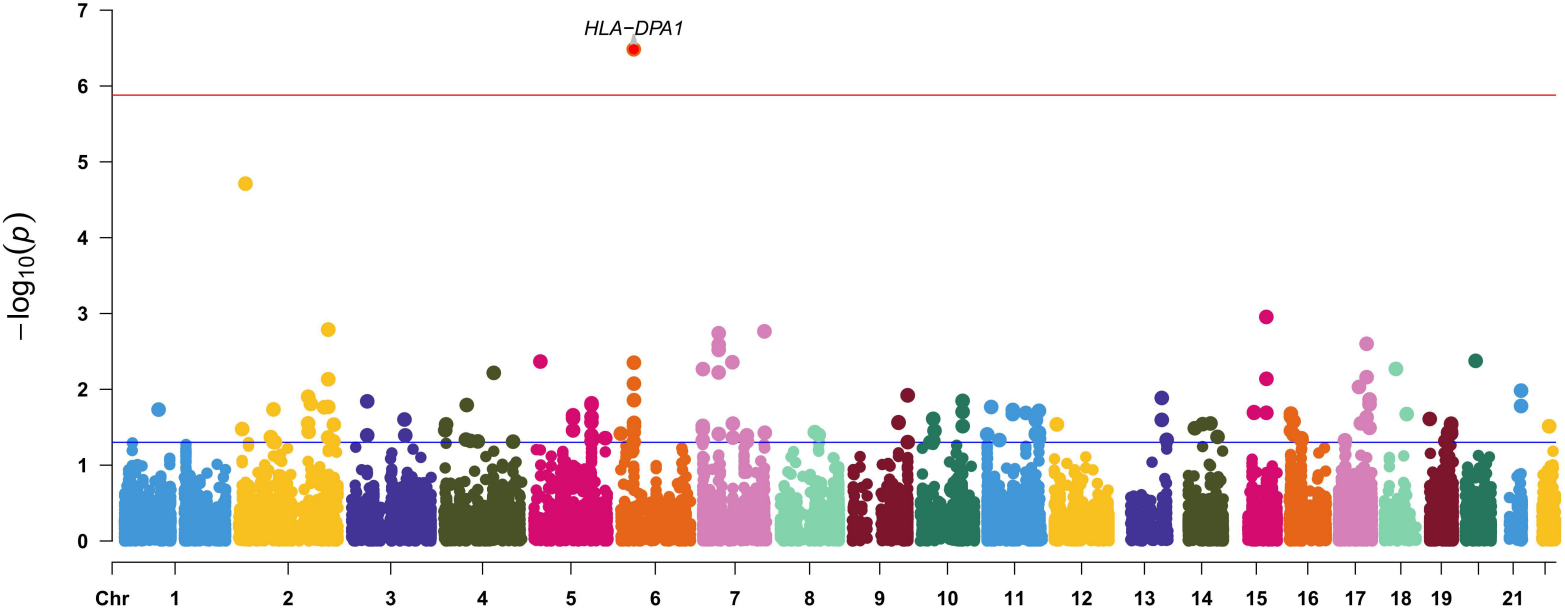
Manhattan plot showing the results of TWAS for genomic-SEM traits using FUSION. The x-axis represents chromosomal positions, and the y-axis shows the -log10(P) values of genetic associations. Each colored point corresponds to a gene locus, with distinct colors denoting different chromosomes. A horizontal red dashed line marks the genome-wide significance threshold, above which associations are considered statistically robust after multiple-testing correction.

**Figure 6:**
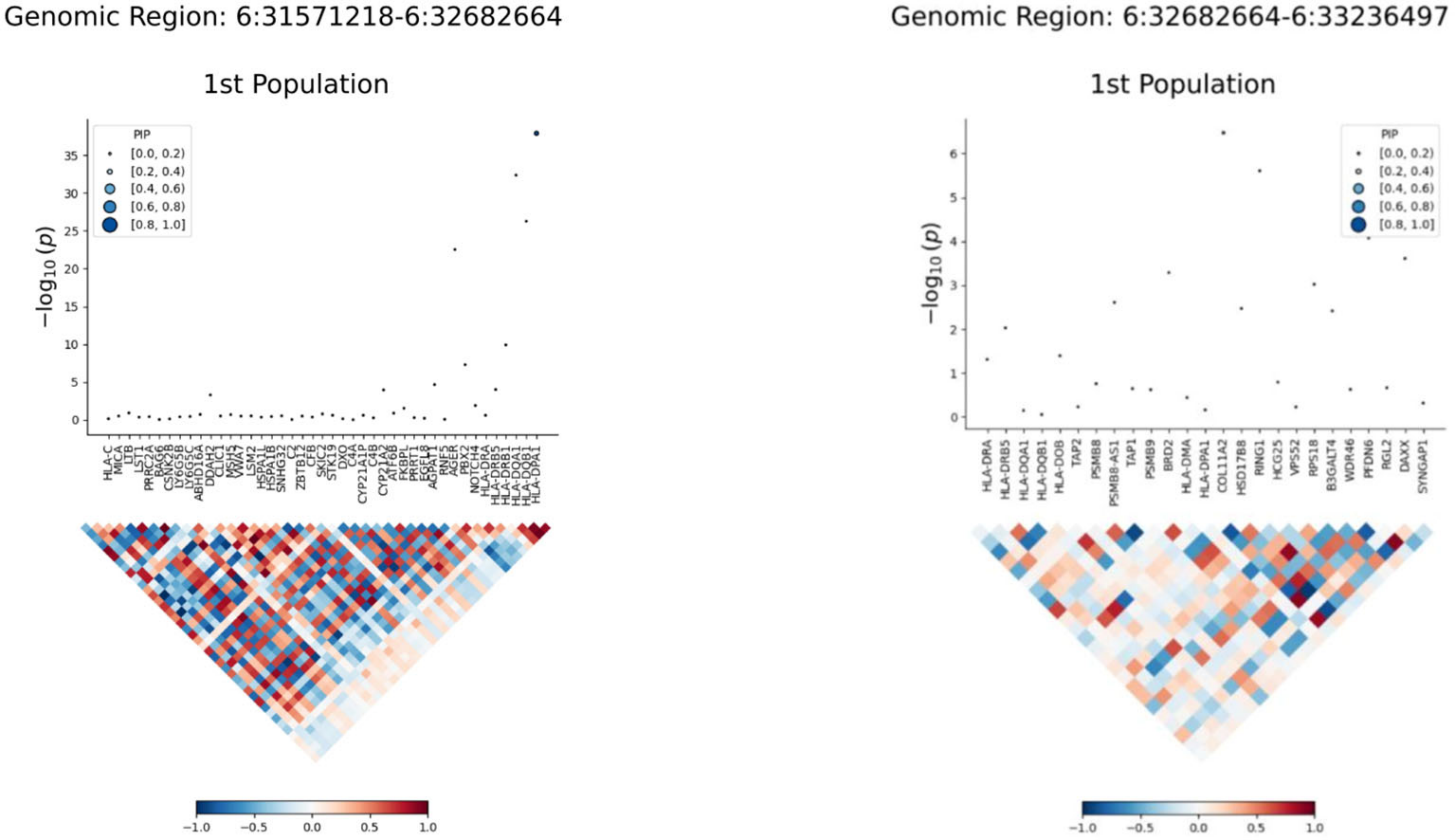
The results of fine-mapping analysis using FOCUS of HLA-DPA1.

Next, we utilized FUSION to conduct TWAS to identify genes associated with the Genomic-SEM traits. We identified HLA-DPA1, which surpassed the threshold adjusted for multiple comparisons (**Figure 5**, detailed TWAS results are provided in **Supplementary File 4**). Subsequently, we performed fine-mapping analysis using FOCUS on the Genomic-SEM data, identifying 116 genes as likely causal signals associated with MGLSc (**Supplementary Table 9**, detailed FOCUS results are provided in **Supplementary File 5**). The results of fine-mapping analysis using FOCUS of HLA-DPA1 are shown in **Figure 6**. To further validate the high-confidence associations at the gene level, we conducted colocalization tests, including HLA-DPA1. HLA-DPA1 showed negative Z-scores (Z < 0), suggesting that the predicted gene expression is negatively correlated with MGLSc. The detailed TWAS and FOCUS colocalization results are summarized in **Supplementary File 6**.

### 3.7 Enrichment analysis of pathways, cell types, and Mendelian disease genes

**Figure 7:**
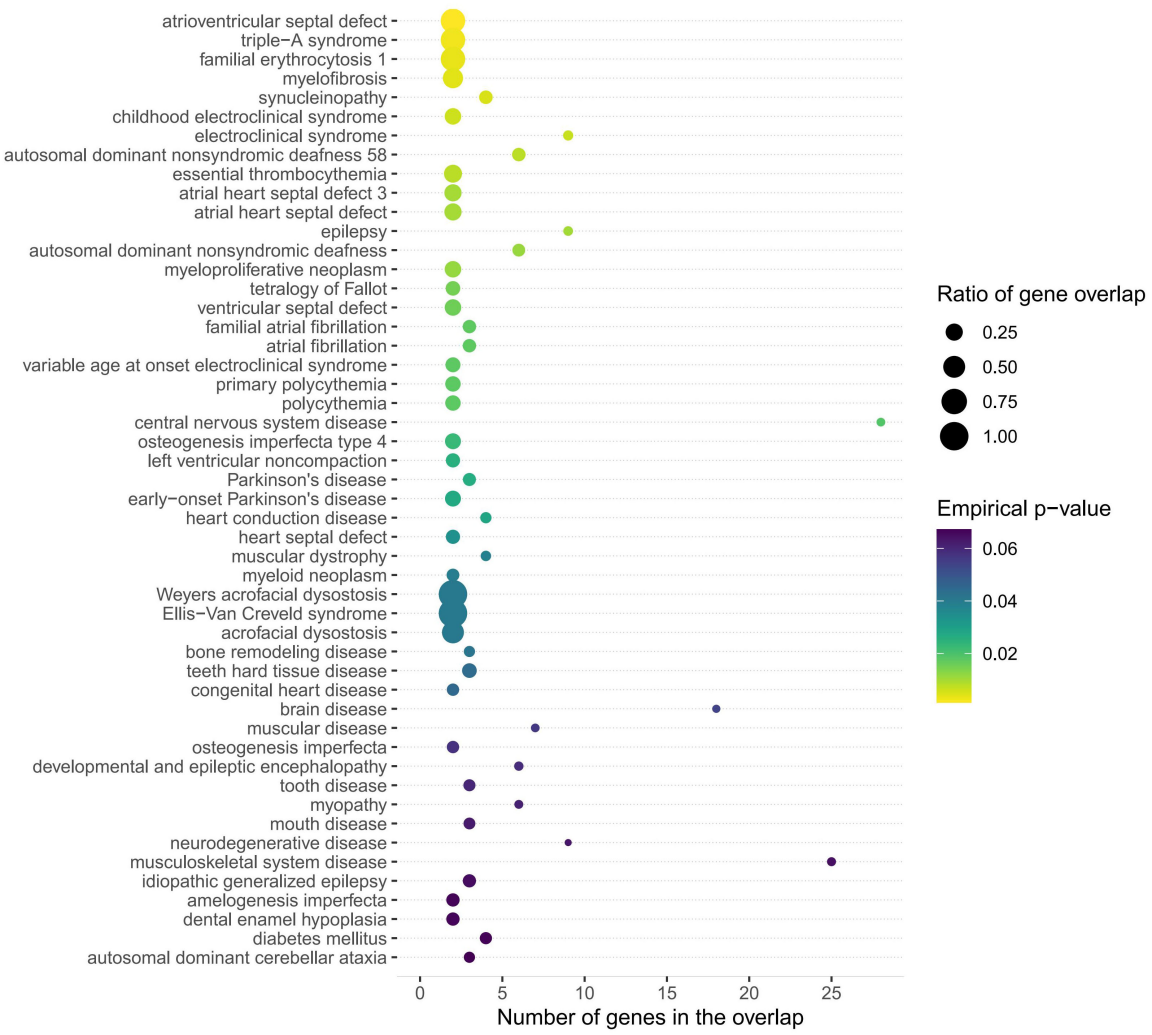
Disease enrichment analysis from the MendelVar database.

**Figure 8:**
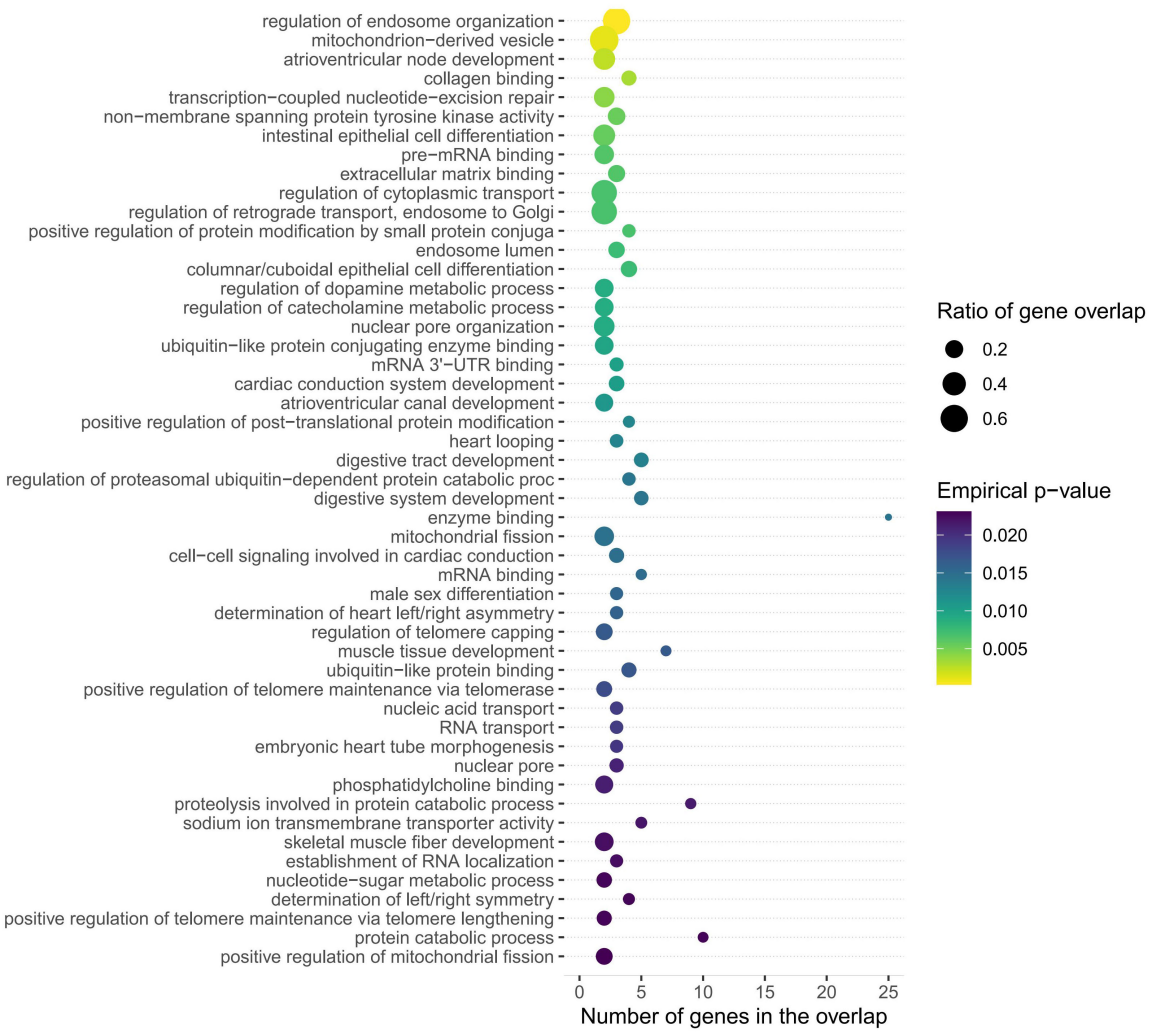
Gene enrichment analysis from the MendelVar database.

MAGMA identified 13 genes through genomic mapping (shown in **Supplementary Table 10**). The results of GSEA of these genes revealed significant enrichment of specific entries (shown in **Supplementary Table 11**), suggesting potential associations between MGLSc and these disorders. The enriched diseases included ulcerative colitis (43, 44), idiopathic membranous nephropathy (45, 46), asthma (47, 48), and autoimmune thyroid diseases (49–51), all of which are linked to immune dysfunction (52). MendelVar enrichment results are presented in **Figure 7** and **Figure 8**. Highly significant gene-overlapped disorders identified by MendelVar included autosomal dominant cerebellar ataxia, myotonic dystrophy, and Weyer’ s acrofacial dysostosis. The biological processes enriched by MendelVar primarily involved regulation of endosome organization, mitochondrial fission, transcription-coupled nucleotide-excision repair, and regulation of cytoplasmic transport, implicating MGLSc in potential links to cellular energy metabolism, transcriptional regulation, DNA repair, and signaling pathways. Notably, cell-type enrichment analyses across diverse cell types did not yield results surviving multiple testing corrections.

### 3.8 Heritability contributions across genomic regions​

To investigate the contribution of genetic heritability across genomic regions, we employed LDSC to analyze functional genomic regions. Our findings revealed that the majority of heritability-contributing loci were enriched in coding regions and expression regulatory regions of chromosomes—regions that typically serve as critical hubs for gene expression regulation, chromatin modification, and transcription factor binding. Notably, genetic variants in coding regions and enhancer regions exhibited the most pronounced effects, suggesting these regions likely exert critical roles in shaping traits or disease susceptibility by regulating gene expression levels. Furthermore, certain conserved sequence regions displayed moderate heritability contributions, implying their potential involvement in complex genetic mechanisms through modulating gene expression or function (shown in **Supplementary Table 12**).

### 3.9 Polygenic risk scores results

Our analyses revealed that PRS variants were strongly associated with the risk of MGLSc, with significant heterogeneity in genetic contributions observed across distinct chromosomal regions (shown in **Supplementary Table 13**). Notably, chromosomes 3 and chromosomes 13 exhibited the highest genetic contributions, potentially harboring critical genes and regulatory elements that influence disease susceptibility. At the SNP level, we identified the top 1,000 SNPs contributing most significantly to MGLSc risk. Among these, 169 SNPs were located on chromosome 6, constituting the majority, followed by 85 SNPs on chromosome 4 and 83 SNPs on chromosome 1. Importantly, the SNPs on chromosome 6 with prominent contribution advantages demonstrated markedly stronger effects on MGLSc risk compared to those on other chromosomes. These findings align with results validated by FINEMAP and MAGMA, further reinforcing the robustness of our conclusions.

### 3.10 Sensitivity analysis results excluding autoimmune traits

As shown in **Table 4**, the fit of this restricted model was visibly degraded compared to the full model. While the chi-square test did not reach the conventional significance threshold (*P* = 0.088), the CFI declined from 1.00 to 0.94 and the SRMR increased from 0.144 to 0.269, both indicating a poorer fit. Additionally, the increased AIC value (from 18.0 to 20.9) further suggests that the exclusion of autoimmune data degraded the model’ s accuracy.

**Table 4:** Fit Indices for Urogenital Traits Only Genomic-SEM Model.

chisq	df	*P* chisq	AIC	CFI	SRMR
4.87071312256224	2	0.087566518650896	20.8707131225622	0.94	0.268896616153148

chisq, Chi-Square Test Statistic; df, Degrees of Freedom; p chisq, p-value for the Chi-Square Test; AIC, Akaike Information Criterion; CFI, Comparative Fit Index; SRMR, Standardized Root Mean Square Residual.

Despite the reduced statistical power, the effect size and direction of the MHC signal remained consistent. The association with HLA-DPA1 remained detectable, albeit with a slightly diminished significance. These findings suggest that while local urogenital factors contribute to MGLSc susceptibility, the systemic autoimmune genetic background is indispensable for capturing the full genetic architecture of the disease. The detailed TWAS results were shown in **Supplementary File 7**.

### 3.11 Validation of prognostic gene expression

**Figure 9:**
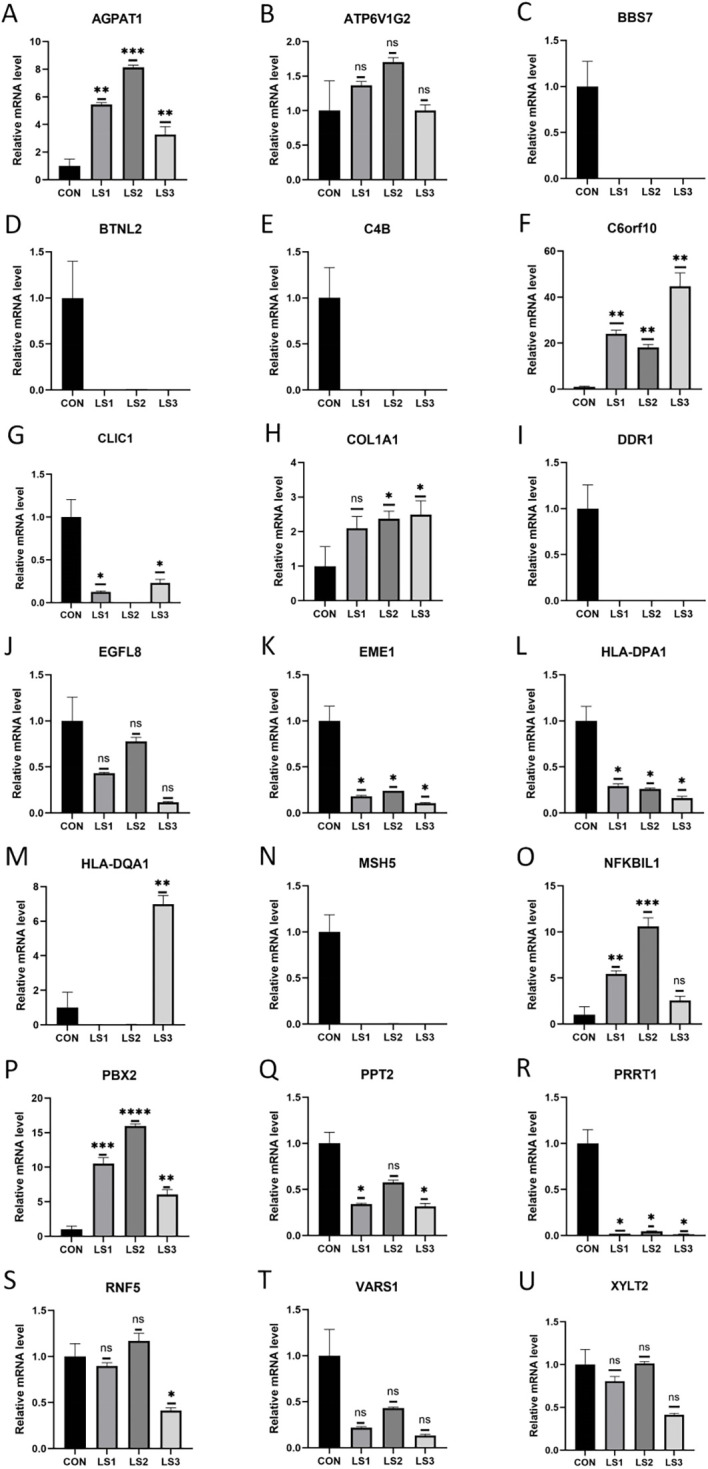
Experimental validation of prognostic gene expression patterns in MGLSc **(A–U)**. (RT-qPCR confirmation using 1 normal male vulvar specimen and 3 MGLSc specimens. Bar graphs quantify concordant expression trends). * P < 0.05; ** P < 0.01; *** P < 0.001.

Due to the rarity of MGLSc and the difficulty in acquiring normal male genital tissue, RT-qPCR validation was performed using one normal male vulvar specimen​ and three MGLSc tissue specimens. To ensure robustness, each sample was run in technical triplicates. Consistent with the bioinformatics predictions, HLA-DPA1expression was significantly decreased in all three MGLSc samples compared to the single control (**Figure 9**).

To validate the prioritized genes experimentally, we performed RT-qPCR *in vitro* for HLA-DPA1, which was jointly supported by TWAS and FOCUS fine-mapping, as well as for selected genes identified by MAGMA enrichment analysis. HLA-DPA1 expression was significantly lower in MGLSc samples than in control samples (*P* < 0.0001), and its direction of change was consistent with the transcriptomic prediction, supporting the robustness of the integrative bioinformatics framework.

Among the MAGMA-prioritized genes, C4B, DDR1, BBS7, VARS1, PRRT1, PPT2, EGFL8, BTNL2, and EME1 showed decreased expression in MGLSc samples relative to controls, whereas AGPAT1 and PBX2 were upregulated. In contrast, ATP6V1G2 and RNF5 did not show obvious differences between groups. Overall, the RT-qPCR results were broadly concordant with the bioinformatics analyses, particularly for HLA-related immune genes, and further support the involvement of immune dysregulation in the pathogenesis of MGLSc. All RT-qPCR results are presented in **Figure 9**.

## 4 Discussion

MGLSc, a chronic inflammatory dermatosis, exerts a significant impact on men’s health-related quality of life, manifesting as pruritus, urinary tract infections, and dyspareunia, in severe cases, it may lead to urethral stricture and secondary voiding difficulties (1, 5, 53–56). Furthermore, previous studies have indicated an association between MGLSc and the development of penile squamous cell carcinoma (1, 57, 58). However, due to the high rarity of this condition and the requirement for multidisciplinary collaboration involving dermatology, urology, pediatrics, and other specialties, there is a paucity of clinical research reports, rendering accurate assessment of its exact incidence and prevalence challenging (59, 60). Given the rarity of MGLSc, The clinical identification of MGLSc primarily relies on symptoms and characteristic cutaneous manifestations, with signs ranging from subtle to pronounced (53, 54). These include atrophic leukodermic patches or plaques, or lilac and slightly scaly patches with telangiectasia and sparse purpura. Furthermore, there may be manifestations of lichenoid balanitis, along with structural changes, hardening, and tightness of the foreskin (58, 60). For MGLSc, diagnosis can also be based on samples obtained from circumcision. Nevertheless, histopathological diagnosis of LS itself presents inherent challenges, particularly in early-stage or mild cases, where pathological features may be subtle or atypical (61).

In present, there is a dearth of GWAS data specifically tailored to MGLSc. Conventional research on MGLSc has predominantly concentrated on clinical case investigations (62). Lin and colleagues employed a multi-omics strategy approach to establish a comprehensive cell atlas of MGLSc, integrating scRNA-seq data with GWAS to probe into the cell-type-specific genes implicated in the pathogenesis of MGLSc. Nevertheless, these endeavors were hampered by several limitations. These included the limited quantity of scRNA-seq datasets, the Asian population origin of the study cohorts, and the fact that most GWAS predominantly involved European populations (5, 60). Furthermore, the existing GWAS datasets for lichen sclerosus (LS) incorporate both male and female LS cases and lesion samples from the external genital regions of these groups, rather than being specific to the male genital region. In this research, we employed a novel Genomic-SEM approach to develop a distinctive SEM model customized for MGLSc. This was accomplished by integrating the summary statistics from GWAS of traits associated with MGLSc, thus consolidating the GWAS data for this rare disease. The stability and reliability of the model were thoroughly validated using a series of rigorous methodologies. By exploring the genetic basis of MGLSc-associated traits included in the study, we integrated multiple analytical frameworks: Genomic-SEM, PRS derived from summary statistics, fine-mapping, and transcriptomic analyses. Through the joint analysis of these complex traits, we identified multiple novel genetic markers. We discovered that genetic factors not only contribute to the etiology of MGLSc as a rare disease but may also exert long-term and profound influences on individuals through their manifestations at the gene, cell, and risk-factor levels. This study offers a novel theoretical foundation for comprehending how genetic loci shape MGLSc and provides crucial insights for the implementation of future precision medicine and public health interventions.

Our study, through analysis of Genomic-SEM and construction of a genetic covariance matrix via LDSC, revealed the genetic covariances among the four traits incorporated into the model. The results indicate that these phenotypes share common genetic factors. Both autoimmune diseases and urinary tract infection play pivotal roles in the pathogenesis of MGLSc, with urinary tract infection being particularly significant. Previous work by Panou et al. suggested that chronic irritation of susceptible epithelium due to continuous urine exposure may represent a core mechanism in MGLSc pathogenesis (60), a view corroborated by several phenomena observed in prior clinical studies (1, 5, 63). For instance, in patients with urinary diversion stomas, LS develops on the peristomal skin, a site characterized by concurrent chronic urine exposure and occlusive pathological conditions. In contrast to female LS cases with concurrent perianal lesions, MGLSc, protected by the prepuce and scrotum from urine exposure in this region, avoids involvement there (64–66). Moreover, males circumcised in early childhood exhibit the lowest incidence of MGLSc, while those circumcised later show a slightly elevated probability (64, 65). Autoimmune diseases are also considered to play a significant role in MGLSc pathogenesis. LS demonstrates a high familial predisposition (67, 68). Through genetic evaluation of LS cohorts, Gao et al. reported that the presence of specific human leukocyte antigen (HLA) alleles and haplotypes (including DQ7, DR12, DQB1, and DRB1*12) is strongly associated with the disease (69). Clinically, LS often co-occurs with a spectrum of mixed autoimmune disorders linked to various serum autoantibodies, such as Hashimoto’ s thyroiditis, rheumatoid arthritis, pernicious anemia, type 1 diabetes mellitus, alopecia areata, vitiligo, and morphea (70–73). Simultaneously, prior studies have documented immune dysregulation in vulvar LS, which is regarded as a crucial factor in the disease’s pathogenesis, which is characterized by the up-regulated expression of type I cytokines from helper T cells and the compromised function of regulatory T cells (64, 65, 74). This set of clinical evidence implies that MGLSc may be associated with the dysregulation of immune self-tolerance. SEM further confirmed the complex genetic interconnections among the four univariate traits—redundant prepuce, phimosis/paraphimosis, urethral stricture, urinary tract infection, and autoimmune diseases—implying that these traits do not exist in isolation but are intertwined and act synergistically.

Through follow-up analysis using Genomic-SEM, we further identified several novel SNPs, including rs715299, rs10774625, rs13194770, rs17843583, rs130065, rs3099837, rs3095330, and rs2621358. Combined with FUMA annotation results, it is evident that these newly discovered loci are predominantly located in intergenic or intronic regions, suggesting that abnormalities in non-coding regulatory elements and the gene expression regulation they mediate may play a significant role in the genetic susceptibility to MGLSc. Furthermore, drawing upon existing literature, these genetic loci are not confined to a single disease phenotype but are broadly associated with a range of conditions, including immune-mediated inflammation, autoimmune diseases, metabolic dysregulation, hematological or urinary biomarkers, and psychopathological characteristics. This pattern indicates that the genetic foundations of MGLSc may exhibit substantial pleiotropy and a shared genetic architecture. Notably, the variant rs715299 has been documented in studies on atopic dermatitis and psoriasis, as well as in research concerning coronary artery disease and autism spectrum disorders (21–24, 75), suggesting that this locus could be implicated in both skin barrier regulation and immune inflammation, alongside broader systemic phenotypes (24, 75). In contrast, the evidence regarding rs10774625 more strongly supports its involvement in the immune-metabolic crosstalk pathway. This genetic variant has been documented in studies investigating shared loci across systemic lupus erythematosus, rheumatoid arthritis, and celiac disease (28–33), as well as in research on shared genetic loci between psoriasis and type 2 diabetes (32, 33). Furthermore, it has been implicated in analyses of blood and urine biomarkers (35, 76) and various hematological and tumor-related traits (25, 27, 77–79). These findings suggest that rs10774625 may not function as a strictly organ-specific risk locus, but rather serves as a pivotal nexus connecting immune dysregulation, chronic inflammation, and metabolic disorders.

Furthermore, other novel single SNPs are more likely to represent mediating mechanisms associated with MGLSc, as opposed to direct pathogenic pathways. Previous GWAS focusing on serum proteins have identified rs130065 and rs3099837, suggesting that these variants may indirectly influence disease progression through modulation of circulating protein levels, inflammatory mediators, or tissue remodeling processes (36). The variant rs3095330 has been associated with shared genetic risk for schizophrenia, well-being factors, and various psychiatric disorders (37–39). Additionally, rs13194770 has been identified in investigations of mental or behavioral phenotypes (41, 42), and rs2621358 has been reported to confer susceptibility to complex diseases (40). These findings indicate that the genetic architecture of MGLSc may not be solely attributable to localized urogenital lesions, but is rather co-modulated by immunological and inflammatory status, systemic protein homeostasis, and psychobiological networks. Collectively, the novel SNPs identified in this study imply that MGLSc likely possesses a complex genetic underpinning driven by immune dysregulation, autoimmune susceptibility, metabolic disturbances, and multi-tiered mediating pathways, thereby offering novel candidate loci for subsequent functional validation and mechanistic investigation.

Notably, the finely mapped variants are predominantly concentrated within the major histocompatibility complex (MHC) region on chromosome 6, indicating that the primary genetic association with MGLSc is likely attributable to immune-related loci rather than to isolated non-immune variants. Among these, the signal encompassing HLA-DQA1 is of particular significance, as HLA class II molecules play a pivotal role in antigen presentation and have been extensively documented in relation to susceptibility to chronic inflammatory and autoimmune disorders (80, 81). This interpretation is also consistent with previous immunogenetic observations regarding LS, which generally report an enrichment of HLA-DQ7 and describe increased frequencies of HLA-DR11, HLA-DR12, and DQ7 in MGLSc, supporting a disease context associated with abnormal adaptive immune activation (69, 82, 83). Furthermore, rs3134608 and rs3134952 are located near C6orf10, a locus situated in the MHC class II region, together with nearby immune regulatory genes such as BTNL2, this region has been repeatedly associated with susceptibility to immune-mediated diseases and T-cell regulation, suggesting that the observed associations may reflect a broader HLA-centered regulatory dysregulation (84, 85). Given the extensive linkage disequilibrium and multilocus structure within the MHC region, traditional GWAS peaks alone are often insufficient to identify the most likely causal variants. However, FINEMAP and SuSIE can prioritize variants with higher posterior support and narrow the candidate set down to more interpretable credible intervals (11, 12). Consequently, our fine-mapping results not only refine the association peaks into a finite number of credible variants but also further support the view that abnormal antigen presentation and immune dysregulation are the primary biological mechanisms underlying MGLSc.

Through FUSION transcriptomic analysis, we further identified potential pathogenic genes associated with these SNPs, including COL1A1, HLA-DPA1, CLIC1, HLA-DQA1, DDR1, BBS7, and VARS1. Most of these genes are involved in critical biological pathways such as inflammatory responses and immune regulation, and are closely linked to various autoimmune diseases (86, 87) (e.g., asthma, systemic sclerosis) as well as established disease-related pathways including breast cancer and lung cancer (88–90). For example, HLA-DPA1 has been implicated in susceptibility to immune-mediated diseases (91). Inamoto et al. demonstrated an association between HLA-DPA1 and sclerotic graft-versus-host disease (GVHD), a distinctive phenotype of chronic GVHD following allogeneic hematopoietic cell transplantation characterized by fibrosis of the skin or fascia. Ji et al. further identified HLA-DPA1 as a crucial biomarker associated with the development and advancement of skin cutaneous melanoma (SKCM). Additionally, HLA-DPA1 has been linked to an increased susceptibility to asthma and systemic sclerosis, underscoring its broad relevance to immune-mediated pathologies (86). Elevated COL1A1 expression has been consistently associated with cancer progression in multiple studies. Zhang et al. confirmed that COL1A1 mutations strongly correlate with substantial infiltration of immune cells, including neutrophils, CD4+ T cells, and dendritic cells, indicating its role in shaping the tumor immune microenvironment. Furthermore, both Zhang and Mohan et al. highlighted that the extracellular matrix (ECM) and cell-substrate junctions are significantly enriched in high COL1A1 expression groups, suggesting that COL1A1 may modulate local immune cell infiltration by affecting the ECM. This finding aligns with the work of Lin et al., who reported that COL1A1 interacts with CD44 to promote T cell migration to the dermo-epidermal junction, leading to a marked increase in T cell proportions in LS tissues. Subsequently, T cell exhaustion under collagen stimulation further exacerbates local chronic inflammation. Genes associated with epidermal cell migration, proliferation, differentiation, matrix synthesis, and remodeling, such as DDR1 and CLIC1, were also verified in this study. As a member of the CLIC family, CLIC1 demonstrates variable expression across the majority of human tissues (92, 93). CLIC1 participates in multiple stages of angiogenesis, including facilitating endothelial cell migration, proliferation, and the formation of capillary-like structures and branches (94). The knockout of CLIC1 leads to a reduction in cell growth, a moderately increased cell viability, and an inhibited network formation, highlighting its crucial role in vascular development (94). Collectively, these transcriptomic analyses provide novel insights into how the immune system and vascular endothelium regulate disease progression. The identified pathways are likely to play a pivotal role in shaping the genetic basis of MGLSc phenotypes, bridging the gap between genetic variation and clinical manifestations of this rare disease.

Through the analysis of GWAS data, we identified multiple risk chromosomal regions associated with the disease. Many of these regions harbor loci within protein-coding regions. Studies have shown that these regions influence diverse biological processes, including cell proliferation, differentiation, and immune responses, by regulating the expression of nearby genes. For instance, we identified nearby genes regulated by multiple risk loci on chromosomes 6 and 17, including NFKBIL1, HLA - DQB1, HLA - DQA1, and EME1, etc. A sub-set of these loci is associated with immune and immunoinflammatory responses. For instance, NFKBIL1, which is situated in the HLA class III region on the short arm of chromosome 6, encodes an inhibitor of kB-like protein (IkBL) (95–99). Numerous studies have verified the associations between NFKBIL1 genetic variants and susceptibility to inflammatory and/or autoimmune diseases, including multiple sclerosis, rheumatoid arthritis (RA), type 1 diabetes, Takayasu’ s arteritis, and chronic thromboembolic pulmonary hypertension (100). Other identified genes are correlated with DNA repair and endothelial cell activity. For instance, EME1 is a crucial factor in upholding genome stability, fulfilling multiple essential functions in DNA repair and resolving intricate DNA structures (101, 102). Specifically, EME1 promotes the repair of meiotic failure induced by inappropriate DNA processing subsequent to double-strand breaks (DSBs), which are among the most severe forms of DNA damage (101, 103, 104). Moreover, the findings of Eldeen et al. demonstrated a significant negative correlation between EME1 and endothelial cells across multiple cancer types, such as bladder cancer, breast cancer, colon adenocarcinoma, kidney renal clear cell carcinoma, lung adenocarcinoma, pancreatic adenocarcinoma, sarcoma, stomach adenocarcinoma, thyroid carcinoma, and thymoma (105). Endothelial cells play a vital role in tumor angiogenesis and metastasis by secreting diverse substances into the bloodstream to facilitate dissemination to distant sites (106, 107). During the identification of risk chromosomal regions, we also observed that altered expression of non-coding RNAs in certain risk regions is closely correlated with disease susceptibility. This suggests that these regions may influence disease pathogenesis not only by modulating gene expression but also by regulating RNA levels.

In this study, we validated via RT-qPCR the HLA-DPA1 gene, which was identified through fine-mapping using TWAS combined with FOCUS, as well as candidate genes identified through MAGMA enrichment analysis. The results showed that HLA-DPA1 was significantly downregulated in the MGLSc group, and the direction of this change was consistent with the results of bioinformatics analysis. Simultaneously, most of the MAGMA-prioritized genes also exhibited disease-associated expression changes in the patient cohort. Among these, C4B, DDR1, BBS7, VARS1, PRRT1, PPT2, EGFL8, BTNL2, and EME1 showed an overall downward trend, while AGPAT1 and PBX2 showed an upward trend and no significant differences were observed for ATP6V1G2 and RNF5. Collectively, the RT-qPCR results provide experimental support for the reliability of the aforementioned multi-omics integrated analysis and indicate that molecular abnormalities in MGLSc are predominantly concentrated in pathways related to HLA-associated antigen presentation, the complement system, T-cell immune regulation, and inflammation-fibrosis-related pathway. Previous studies on LS have repeatedly pointed out that its pathogenesis is closely related to immune abnormalities and HLA susceptibility, with genetic associations in the HLA class II region observed in both male and female genital LS (83, 108–110). Among these, the downregulation of HLA-DPA1 is of particular significance. HLA-DPA1 constitutes a component of the HLA class II molecule, which is integral to the presentation of exogenous antigens and the activation of CD4+ T lymphocytes, thereby forming a critical nexus between innate and adaptive immunity. Previous investigations have established associations between HLA-DP loci and a spectrum of immune-mediated disorders, including autoimmune diabetes, primary sclerosing cholangitis, and immune-related adverse events (111–113). Within the LS disease context, while prior investigations have predominantly emphasized HLA-DQ7, DR11, and DR12 alleles, the convergent evidence indicates that aberrations in the HLA region constitute a significant element of the immunogenetic foundation underlying LS (83, 114). Therefore, the significant downregulation of HLA-DPA1 observed in MGLSc tissue in this study suggests that it may not merely represent a statistical association but rather reflects abnormalities in local antigen presentation efficiency, mucosal immune homeostasis, or patterns of inflammatory cell activation. In other words, reduced HLA-DPA1 expression may contribute to the development of persistent inflammation and abnormal immune responses in MGLSc.

In addition to HLA-DPA1, the downregulation of C4B and BTNL2 further supports the immunopathological nature of MGLSc. C4B functions as a pivotal component of the classical complement cascade, and variations in complement C4 copy number and expression have been demonstrated to correlate with systemic lupus erythematosus, Sjögren’ s syndrome, and other systemic inflammatory autoimmune disorders (115–117). BTNL2, a pivotal immunoregulatory molecule within the butyrophilin-like protein family, functions to inhibit T-cell activation and sustain peripheral immune tolerance. Dysregulation of BTNL2 activity is implicated in the pathogenesis of diverse immune-mediated disorders (85). Therefore, the low expression of C4B and BTNL2 observed in this study indicates that MGLSc may involve both complement regulation imbalance and insufficient T-cell negative regulation. This finding corroborates the previously reported pathological features of LS, which comprise Th1 bias, elevated T-cell infiltration, and amplified autoimmune phenotypes (109, 118).

In our study, we also found that DDR1 tends to be downregulated in MGLSc. DDR1 is a collagen-receptor tyrosine kinase involved in cell-extracellular matrix interactions, inflammatory signaling, and fibrotic remodeling (119). Previous studies have shown that DDR1 is associated with inflammatory and fibrotic processes in various organs. In models of renal and pulmonary tissue injury, DDR1 activation promotes inflammatory cell recruitment, cytokine release, and collagen deposition (119–123). Given that one of the core pathological changes in MGLSc is sclerosis and scar-like remodeling against a background of chronic inflammation, the abnormal expression of DDR1 suggests its potential involvement in the matrix remodeling-inflammation coupling process of the disease. However, while DDR1 was downregulated in this study, it has often been upregulated in a pro-inflammatory/pro-fibrotic upregulation observed in previous fibrotic models (123). This suggests that the role of DDR1 in MGLSc may be tissue- and stage-specific, its downregulation may reflect impaired epithelial-stromal interactions in chronic-phase lesions, or it may indicate that the regulatory mechanisms of DDR1 vary across different tissues. Further clarification is required through protein localization and functional experiments.

As for the upregulation of AGPAT1 and PBX2, this suggests that, in addition to immune abnormalities, MGLSc may also be associated with metabolic reprogramming and alterations in transcriptional regulatory networks. PBX2, located near the MHC region, belongs to the heterologous box transcription factor family and plays a potential role in the regulation of immune-related genes and developmental regulation; AGPAT1, on the other hand, is associated with phospholipid metabolism and membrane lipid remodeling, and its upregulation may be related to changes in membrane lipid metabolism, cellular proliferation and repair, or stress responses in local tissues under chronic inflammatory stimulation. In contrast, no significant differences were observed for ATP6V1G2 and RNF5, suggesting that not all bioinformatics-prioritized genes exhibit stable transcriptional changes under the current sample and testing conditions. This phenomenon is not contradictory, as GWAS/TWAS/MAGMA prioritization reflects genetic contributions and regulatory tendencies at the population level, whereas RT-qPCR validation is influenced by factors such as sample size, tissue heterogeneity, disease stage, and post-transcriptional regulation.

Overall, the RT-qPCR validation results indicate that the significant downregulation of HLA-DPA1 represents the most robust experimental evidence in this study, while changes in the expression of genes such as C4B, BTNL2, and DDR1 further support the immune-mediated nature of MGLSc from the perspectives of complement regulation, T-cell immunosuppression, and inflammatory-fibrotic remodeling. These findings not only reinforce the credibility of the TWAS, FOCUS, and MAGMA analyses but also suggest that the molecular mechanisms underlying MGLSc may not be driven by a single pathway, but rather constitute a complex interplay of HLA-region genetic susceptibility, local antigen presentation abnormalities, immune regulatory imbalance, and tissue remodeling responses. Future studies will require larger sample sizes combined with protein-level validation, single-cell resolution analysis, and functional experiments to further clarify the specific roles of these candidate genes at different stages of MGLSc progression.

Notwithstanding the novel insights provided into the genetics of MGLSc, a rare disease, this study has several limitations. First, the sample population comprised primarily individuals of European ancestry, lacking validation in other ethnic groups. Besides, MGLSc exclusively affects males, the use of female-inclusive summary statistics may have diluted the detection of male-specific genetic effects, future studies utilizing male-only biobank data or targeted sequencing of male MGLSc patients are warranted to validate these findings. Moreover, due to inherent limitations of the GWAS summary statistics used, further stratification of the included cohorts was not feasible. Future studies should therefore expand the cohort size and corroborate these genetic associations across diverse ethnic and geographical populations to ensure broader generalizability. Another limitation is the small sample size for experimental validation, while the consistent downregulation observed across three independent patient samples supports the biological relevance of HLA-DPA1, future studies with larger, matched cohorts are necessary to confirm these findings and exclude potential batch effects. Meanwhile, although multiple genetic loci associated with MGLSc were identified through fine-mapping and transcriptomic analyses, elucidating the precise biological mechanisms underlying these associations remains a formidable challenge. Subsequent research should aim to elucidate the mechanistic pathways through which these genetic variants influence MGLSc susceptibility—such as through modulation of gene expression, neural development, metabolic pathways, and other cellular processes. Furthermore, while our study underscores the significance of genetic factors in MGLSc, the potential contribution of environmental factors also warrants further investigation. Future studies ought to examine how gene-environment interactions modulate disease expressivity, thereby enhancing our understanding of the complex interplay between genetic predisposition and environmental exposures in the pathogenesis of MGLSc.

## 5 Conclusion

This study offers novel perspectives on the genetic architecture underlying MGLSc. By integrating Genomic-SEM, fine-mapping, and transcriptomic analyses, we identified multiple novel genetic loci and delineated their roles in mediating genetic correlations between gene expression and complex traits. Furthermore, by combining TWAS with FOCUS fine-mapping, we precisely localized HLA-DPA1 as the prioritized candidate gene for MGLSc, and *in vitro* experiments further corroborated the reliability of our analytical conclusions. These results not only advance the mechanistic understanding of MGLSc pathogenesis but also suggest potential pathways for precision medicine and public health strategies. Future research will prioritize the validation of these genetic markers and elucidate the contribution of gene-environment interactions to health and educational outcomes, with the ultimate objective of enhancing global healthspan.

## Data Availability

The original contributions presented in the study are included in the article/**Supplementary Material**. Further inquiries can be directed to the corresponding authors.

## Glossary

AIC: Akaike Information Criterion
CFI: Comparative Fit Index
CELLECT: CELL-type Expression-specific integration for Complex Traits
CELLEX: CELL-type EXpression-specificity
DSBs: Double-Strand Breaks
ECM: Extracellular Matrix
eQTL: Expression Quantitative Trait Loci
FDR: False Discovery Rate
FINEMAP: Fine-Mapping Tool
FOCUS: Fine-mapping Of Causal gene Sets
FUMA: Functional Mapping and Annotation
Genomic-SEM: Genomic Structural Equation Modeling
GSEA: Gene Set Enrichment Analysis
GTEx: Genotype-Tissue Expression Project
GVHD: Graft-Versus-Host Disease
GWAS: Genome-Wide Association Study
HLA: Human Leukocyte Antigen
IRBs: Institutional Review Boards
LD: Linkage Disequilibrium
LDSC: Linkage Disequilibrium Score Regression
LS: Lichen Sclerosus
MAF: Minor Allele Frequency
MAGMA: Multi-marker Analysis of Genomic Annotation
MGLSc: Male Genital Lichen Sclerosus
MHC: Major Histocompatibility Complex
PIP: Posterior Inclusion Probability
PRS: Polygenic Risk Scores
QC: Quality Control
RA: Rheumatoid Arthritis
RT-qPCR: Reverse Transcription Quantitative PCR
scRNA-seq: Single-Cell RNA Sequencing
SEM: Structural Equation Modeling
SKCM: Skin Cutaneous Melanoma
SNP: Single-Nucleotide Polymorphism
SRMR: Standardized Root Mean Square Residual
SuSIE: Sum of Single Effects
TWAS: Transcriptome-Wide Association Study.
