# Supplementary material for: Integrating genomic structural equation modeling and experimental validation to unravel the genetic basis of male genital lichen sclerosus
Source: Front Immunol. 2026 Jul 16;17:1857945. doi: 10.3389/fimmu.2026.1857945 (PMC13435250; doi:10.3389/fimmu.2026.1857945)
Supplement: Supplementary file 1 [file Table1.docx]

**Supplementary Table 1.** SNP heritability of Genomic-SEM phenotypes

| **Phenotype** | **N SNPs** | **h^2^ (se)** | **λGC** | **Mean ChiSquare** | **Intercept se** | **Ratio se** | **h^2^ Z** |
| --- | --- | --- | --- | --- | --- | --- | --- |
| Redundant prepuce, phimosis and paraphimosis | 596,135 | 0.0012 (0.0012) | 1 | 1.0164 | 1.0036 (0.0101) | 0.2173 (0.6164) | 0.98 |
| Urethral stricture | 728,151 | 0.0016 (0.0011) | 1 | 1.0236 | 1.0075 (0.0091) | 0.3174 (0.3843) | 1.41 |
| Urinary tract infection | 1,030,428 | 0.0017 (0.001) | 1.0475 | 1.038 | 1.0212 (0.0075) | 0.5589 (0.1974) | 1.74 |
| Autoimmune diseases | 1,161,182 | 0.0516 (0.0046) | 1.4479 | 1.7126 | 1.1924 (0.0203) | 0.2701 (0.0285) | 11.3 |

Abbreviations: h^2^ = SNP heritability, se = Standard error, λGC = Genomic inflation factor, h^2^Z = Z-score for the heritability estimate

**Supplementary Table 2.** Factor Loadings and Residual Covariances in the Genomic-SEM of Male Genital Lichen Sclerosus

| **lhs** | **op** | **rhs** | **Unstandardized Est** | **Unstandardized SE** | **Standardized Est** | **Standardized SE** | ***P* value** |
| --- | --- | --- | --- | --- | --- | --- | --- |
| F1 | =~ | Redundant prepuce, phimosis and paraphimosis | -0.0604725689909809 | 0.0799713469628571 | -0.295279070919289 | 0.39048725080033 | 0.449542512798455 |
| F1 | =~ | Urethral stricture | -0.21191800198515 | 0.0829622060035465 | -0.934775341763359 | 0.365948128060347 | 0.0106373399233914 |
| F1 | =~ | Urinary tract infection | -0.153423165574494 | 0.0588373526788195 | -0.846557523003816 | 0.324652369121075 | 0.00911844819683568 |
| F1 | =~ | Autoimmune diseases | -0.0672506137017494 | 0.0265203845183954 | -0.251739685307508 | 0.0992739083138979 | 0.0112188098164498 |
| Redundant prepuce, phimosis and paraphimosis | ~~ | Redundant prepuce, phimosis and paraphimosis | 0.0382854645167638 | 0.0400746546486669 | 0.912810314680158 | 0.955464877964851 | 0.339398923740443 |
| Urethral stricture | ~~ | Urethral stricture | 0.00648601597276421 | 0.044882620647075 | 0.126195030783507 | 0.873285883938895 | 0.885097259742322 |
| Urinary tract infection | ~~ | Urinary tract infection | 0.00930616408632574 | 0.0213767357301083 | 0.28334037579436 | 0.650837851389142 | 0.66331511307666 |
| Autoimmune diseases | ~~ | Autoimmune diseases | 0.0668430024606239 | 0.00758624727693585 | 0.936627124034168 | 0.106301096215115 | 1.23950126245758e-18 |

Abbreviations: lhs = Left-hand side variable, rhs = Right-hand side variable, op = Operator ("=~" denotes measurement relationship (factor loading), and "~~" denotes covariance relationship, Unstandardized Est = Unstandardized factor loading or variance estimate, Unstandardized SE = Standard error of the unstandardized estimate, Standardized Est = Standardized factor loading or variance estimate, Standardized SE = Standard error of the standardized estimate.

**Supplementary Table 3.** SNP Variants Identified by Genomic-SEM (*P* value < 5×10^-12^)

| **Number** | **SNP** | **CHR** | **BP** | **Eaf** | **Effect allele** | **Other allele** | **Beta** | **SE** | ***P* value** | **N** |
| --- | --- | --- | --- | --- | --- | --- | --- | --- | --- | --- |
| 1 | rs6671315 | 1 | 62452749 | 0.232604 | G | C | 0.000535363522691084 | 7.62189331436289e-05 | 2.15569638349201e-12 | 310915142889.897 |
| 2 | rs11124863 | 2 | 42460163 | 0.326044 | G | C | -0.000803544 | 7.05860357004919e-05 | 5.02997075236964e-30 | 310915142889.897 |
| 3 | rs1432297 | 2 | 61070652 | 0.451292 | G | A | 0.000537083272276102 | 6.10895189323678e-05 | 1.47258992066522e-18 | 310915142889.897 |
| 4 | rs2950611 | 2 | 140902383 | 0.252485 | A | G | 0.00050649456093471 | 7.01240874188325e-05 | 5.09155901644181e-13 | 310915142889.897 |
| 5 | rs35865016 | 3 | 165715990 | 0.410537 | C | A | -0.000454928 | 5.98105047657349e-05 | 2.82367703825173e-14 | 310915142889.897 |
| 6 | rs6842718 | 4 | 5942417 | 0.464215 | G | C | -0.00041643 | 5.27867300287673e-05 | 3.04822724551819e-15 | 310915142889.897 |
| 7 | rs260120 | 4 | 29409982 | 0.372763 | G | A | 0.00049010059458016 | 6.87694510021041e-05 | 1.02789186370492e-12 | 310915142889.897 |
| 8 | rs11097189 | 4 | 76886398 | 0.423459 | G | A | 0.000503923536553328 | 6.70097046222405e-05 | 5.47102709161381e-14 | 310915142889.897 |
| 9 | rs58817164 | 4 | 91669114 | 0.480119 | A | C | 0.000518021537418551 | 5.85827516056805e-05 | 9.35494420482227e-19 | 310915142889.897 |
| 10 | rs11098638 | 4 | 122600522 | 0.43837 | G | A | -0.002486038 | 6.81071262001847e-05 | 1e-200 | 310915142889.897 |
| 11 | rs2844776 | 6 | 30171827 | 0.182903 | T | C | -0.000571905 | 7.8140636375542e-05 | 2.49971782785334e-13 | 310915142889.897 |
| 12 | rs3095330 | 6 | 30693633 | 0.181909 | A | G | -0.000889843 | 7.85835724934465e-05 | 1.00357954315476e-29 | 310915142889.897 |
| 13 | rs2523866 | 6 | 31017347 | 0.400596 | T | C | -0.000469625 | 6.62968938707528e-05 | 1.4038525758302e-12 | 310915142889.897 |
| 14 | rs3094662 | 6 | 31121945 | 0.190855 | A | C | -0.000760847 | 7.66415719846198e-05 | 3.16606346047167e-23 | 310915142889.897 |
| 15 | rs130065 | 6 | 31122500 | 0.191849 | G | A | -0.000884342 | 7.70498810898785e-05 | 1.71097451261583e-30 | 310915142889.897 |
| 16 | rs1062630 | 6 | 31138107 | 0.200795 | G | A | -0.000600399 | 7.52655662085387e-05 | 1.49838344298994e-15 | 310915142889.897 |
| 17 | rs2077010 | 6 | 31138377 | 0.410537 | C | T | -0.000617455 | 6.34472746300655e-05 | 2.20701946622606e-22 | 310915142889.897 |
| 18 | rs2394895 | 6 | 31206979 | 0.213718 | T | C | -0.000733408 | 7.37958451971053e-05 | 2.8353647817832e-23 | 310915142889.897 |
| 19 | rs2442719 | 6 | 31320538 | 0.444334 | C | T | -0.000618091 | 6.69444420544475e-05 | 2.63405532916911e-20 | 310915142889.897 |
| 20 | rs9267091 | 6 | 31413809 | 0.177932 | G | A | -0.000712284 | 7.60791498326304e-05 | 7.7939041107114e-21 | 310915142889.897 |
| 21 | rs9267093 | 6 | 31414262 | 0.249503 | A | T | -0.000749628 | 6.9092898403014e-05 | 2.00385823918821e-27 | 310915142889.897 |
| 22 | rs3099837 | 6 | 31417463 | 0.249503 | G | A | -0.00096301 | 6.89829611170225e-05 | 2.73023013520501e-44 | 310915142889.897 |
| 23 | rs3130475 | 6 | 31424778 | 0.281312 | G | A | -0.000891284 | 6.82810040442178e-05 | 6.09506279923533e-39 | 310915142889.897 |
| 24 | rs2071592 | 6 | 31515340 | 0.294235 | T | A | -0.000889771 | 6.9108628001163e-05 | 6.22701948416991e-38 | 310915142889.897 |
| 25 | rs9267815 | 6 | 32133098 | 0.201789 | A | G | -0.000689683 | 7.77984254081084e-05 | 7.65016270951792e-19 | 310915142889.897 |
| 26 | rs3131297 | 6 | 32141005 | 0.200795 | A | T | -0.000557532 | 7.71674431514726e-05 | 5.012470669996e-13 | 310915142889.897 |
| 27 | rs3134947 | 6 | 32145205 | 0.199801 | C | T | -0.000745327 | 7.78164132767751e-05 | 9.89222039578293e-22 | 310915142889.897 |
| 28 | rs3134944 | 6 | 32146644 | 0.199801 | C | T | -0.000825578 | 7.75092839503852e-05 | 1.71863184506793e-26 | 310915142889.897 |
| 29 | rs3134932 | 6 | 32186147 | 0.377734 | C | A | -0.000644044 | 6.7383780692145e-05 | 1.20237229599337e-21 | 310915142889.897 |
| 30 | rs715299 | 6 | 32189841 | 0.265408 | T | G | -0.001019732 | 6.97764827041386e-05 | 2.27795331245492e-48 | 310915142889.897 |
| 31 | rs915894 | 6 | 32190390 | 0.351889 | T | G | -0.000627812 | 6.74037022130564e-05 | 1.22860917246581e-20 | 310915142889.897 |
| 32 | rs3117133 | 6 | 32313653 | 0.223658 | G | C | 0.000649044992933614 | 6.0559548743867e-05 | 8.42794351416274e-27 | 310915142889.897 |
| 33 | rs2073045 | 6 | 32339548 | 0.350895 | G | A | -0.000693968 | 7.024030629164e-05 | 5.08744411604573e-23 | 310915142889.897 |
| 34 | rs1967688 | 6 | 32340068 | 0.479125 | C | T | -0.001063851 | 6.63687108858689e-05 | 7.96417917392691e-58 | 310915142889.897 |
| 35 | rs1980496 | 6 | 32340070 | 0.346918 | C | T | -0.00104067 | 6.65907662378309e-05 | 4.70508593547321e-55 | 310915142889.897 |
| 36 | rs9268406 | 6 | 32342129 | 0.27336 | C | T | -0.000512959 | 7.00296335233247e-05 | 2.39094847232163e-13 | 310915142889.897 |
| 37 | rs9268413 | 6 | 32343095 | 0.274354 | T | C | -0.000765691 | 7.17271403869392e-05 | 1.33179713960312e-26 | 310915142889.897 |
| 38 | rs9268423 | 6 | 32343882 | 0.27336 | C | T | -0.001006352 | 7.21286728129689e-05 | 3.0517349772369e-44 | 310915142889.897 |
| 39 | rs9268426 | 6 | 32344731 | 0.27336 | A | G | -0.000469207 | 6.71969141019766e-05 | 2.8984588996425e-12 | 310915142889.897 |
| 40 | rs8180659 | 6 | 32347280 | 0.27336 | C | T | -0.001165378 | 7.16952242328434e-05 | 2.07161760937565e-59 | 310915142889.897 |
| 41 | rs9268448 | 6 | 32348463 | 0.27336 | C | G | -0.001137884 | 7.20339183333199e-05 | 3.2889439134239e-56 | 310915142889.897 |
| 42 | rs9268458 | 6 | 32350384 | 0.276342 | C | A | -0.000717549 | 7.09903151107767e-05 | 5.10696605167365e-24 | 310915142889.897 |
| 43 | rs9268462 | 6 | 32351991 | 0.27336 | C | A | -0.000760258 | 7.1834931475964e-05 | 3.55775588676959e-26 | 310915142889.897 |
| 44 | rs9268474 | 6 | 32357165 | 0.27336 | T | C | -0.000672304 | 7.16077572205842e-05 | 6.07447128510305e-21 | 310915142889.897 |
| 45 | rs9268480 | 6 | 32363844 | 0.27336 | C | T | -0.000652475 | 7.10683067941538e-05 | 4.27296091426925e-20 | 310915142889.897 |
| 46 | rs2294880 | 6 | 32367722 | 0.272366 | A | G | -0.000712784 | 7.17883841122801e-05 | 3.1150545512917e-23 | 310915142889.897 |
| 47 | rs3817966 | 6 | 32367847 | 0.276342 | T | C | -0.000790768 | 7.11408695507561e-05 | 1.05433687387043e-28 | 310915142889.897 |
| 48 | rs9268491 | 6 | 32374131 | 0.27336 | C | G | -0.000577813 | 7.16287845078217e-05 | 7.21856507499518e-16 | 310915142889.897 |
| 49 | rs3763307 | 6 | 32374622 | 0.27336 | A | T | -0.001017843 | 7.17788863530135e-05 | 1.21411943928203e-45 | 310915142889.897 |
| 50 | rs9268768 | 6 | 32419291 | 0.441352 | C | T | -0.000498148 | 6.58297411311002e-05 | 3.81299861441411e-14 | 310915142889.897 |
| 51 | rs6457588 | 6 | 32419908 | 0.441352 | A | C | -0.000852239 | 6.62109737954208e-05 | 6.50678397862001e-38 | 310915142889.897 |
| 52 | rs9268835 | 6 | 32428115 | 0.277336 | G | A | -0.000544382 | 7.00579555641231e-05 | 7.82044926152141e-15 | 310915142889.897 |
| 53 | rs9268848 | 6 | 32429303 | 0.484095 | G | A | -0.000819294 | 6.63969257919329e-05 | 5.56124913654021e-35 | 310915142889.897 |
| 54 | rs9268852 | 6 | 32429594 | 0.4334 | A | G | -0.000979909 | 6.74087170152182e-05 | 7.07934657198098e-48 | 310915142889.897 |
| 55 | rs9268858 | 6 | 32429758 | 0.321074 | T | C | -0.00061314 | 6.87119488138286e-05 | 4.52437637963252e-19 | 310915142889.897 |
| 56 | rs9268906 | 6 | 32432181 | 0.321074 | A | G | -0.000738798 | 6.87326156435295e-05 | 5.99919350841451e-27 | 310915142889.897 |
| 57 | rs9268912 | 6 | 32432509 | 0.4334 | C | A | -0.000620949 | 6.71822350781249e-05 | 2.40231764343416e-20 | 310915142889.897 |
| 58 | rs9268923 | 6 | 32432835 | 0.321074 | C | T | -0.000596508 | 6.7974154646994e-05 | 1.70118535975322e-18 | 310915142889.897 |
| 59 | rs2395186 | 6 | 32433192 | 0.322068 | C | G | -0.000602483 | 6.85777418750406e-05 | 1.55803270294064e-18 | 310915142889.897 |
| 60 | rs9268970 | 6 | 32434361 | 0.321074 | C | A | -0.000710861 | 6.88758317769156e-05 | 5.6686740692153e-25 | 310915142889.897 |
| 61 | rs9368726 | 6 | 32438542 | 0.321074 | T | C | -0.000940647 | 6.85795250457935e-05 | 8.12656712133532e-43 | 310915142889.897 |
| 62 | rs9405108 | 6 | 32438648 | 0.32008 | C | T | -0.000936415 | 6.86224357892355e-05 | 2.13538845957803e-42 | 310915142889.897 |
| 63 | rs28366312 | 6 | 32561175 | 0.287276 | T | C | -0.000852819 | 7.18005277382908e-05 | 1.54720902400168e-32 | 310915142889.897 |
| 64 | rs28366315 | 6 | 32561207 | 0.295229 | C | T | -0.000897492 | 6.9926381666755e-05 | 1.04664160471205e-37 | 310915142889.897 |
| 65 | rs28366319 | 6 | 32561495 | 0.300199 | G | A | -0.000885372 | 6.92453511393901e-05 | 1.96273623132812e-37 | 310915142889.897 |
| 66 | rs28366328 | 6 | 32561766 | 0.301193 | A | G | -0.000827139 | 6.92130296087803e-05 | 6.44377093638726e-33 | 310915142889.897 |
| 67 | rs28366331 | 6 | 32561880 | 0.236581 | T | G | -0.001060842 | 8.01938953620185e-05 | 6.01043186950811e-40 | 310915142889.897 |
| 68 | rs28366332 | 6 | 32561885 | 0.236581 | C | A | -0.0011752 | 7.28501122282218e-05 | 1.52625077691646e-58 | 310915142889.897 |
| 69 | rs9270664 | 6 | 32566149 | 0.406561 | G | A | -0.000752591 | 6.8674189939427e-05 | 6.02501412391379e-28 | 310915142889.897 |
| 70 | rs556025 | 6 | 32570880 | 0.32505 | C | T | -0.000976308 | 6.91971457255159e-05 | 3.33930674855597e-45 | 310915142889.897 |
| 71 | rs9270866 | 6 | 32571043 | 0.2833 | G | A | -0.000511611 | 7.37279171050342e-05 | 3.94386371550307e-12 | 310915142889.897 |
| 72 | rs9270895 | 6 | 32571873 | 0.364811 | C | T | -0.000634048 | 7.20155528759323e-05 | 1.31646941074527e-18 | 310915142889.897 |
| 73 | rs9270911 | 6 | 32572202 | 0.463221 | C | T | -0.00069547 | 6.681801990548e-05 | 2.26957574533683e-25 | 310915142889.897 |
| 74 | rs586610 | 6 | 32572311 | 0.275348 | T | C | -0.000573796 | 7.35153134752873e-05 | 5.94415142884205e-15 | 310915142889.897 |
| 75 | rs9270922 | 6 | 32572388 | 0.286282 | T | C | -0.00060038 | 7.36079168193863e-05 | 3.44985904004705e-16 | 310915142889.897 |
| 76 | rs9270935 | 6 | 32572701 | 0.269384 | A | G | -0.000553402 | 7.20978734497873e-05 | 1.64512891340413e-14 | 310915142889.897 |
| 77 | rs9270949 | 6 | 32572975 | 0.286282 | T | C | -0.00088807 | 7.13215233148183e-05 | 1.36990981966984e-35 | 310915142889.897 |
| 78 | rs643889 | 6 | 32575918 | 0.368787 | A | T | -0.000546198 | 6.70064798531808e-05 | 3.59663811692293e-16 | 310915142889.897 |
| 79 | rs9271096 | 6 | 32576426 | 0.274354 | A | G | -0.000597645 | 7.3323656526886e-05 | 3.61602113307625e-16 | 310915142889.897 |
| 80 | rs3129758 | 6 | 32584625 | 0.360835 | A | G | -0.001024333 | 6.86696918954125e-05 | 2.56238163272535e-50 | 310915142889.897 |
| 81 | rs685810 | 6 | 32584952 | 0.355865 | A | G | -0.000545538 | 6.88129372450733e-05 | 2.22980893796067e-15 | 310915142889.897 |
| 82 | rs9271365 | 6 | 32586794 | 0.387674 | T | G | -0.001135214 | 6.59511121089782e-05 | 2.12291838771797e-66 | 310915142889.897 |
| 83 | rs9271475 | 6 | 32588808 | 0.387674 | A | T | -0.000656127 | 6.98883441291381e-05 | 6.10280830768861e-21 | 310915142889.897 |
| 84 | rs9271498 | 6 | 32589282 | 0.293241 | G | A | -0.00069998 | 7.84187571764135e-05 | 4.40960941299803e-19 | 310915142889.897 |
| 85 | rs9271549 | 6 | 32590248 | 0.304175 | T | C | -0.000706504 | 6.88360953549502e-05 | 1.02833404454654e-24 | 310915142889.897 |
| 86 | rs9271563 | 6 | 32590428 | 0.297217 | T | C | -0.000666856 | 7.04563410524878e-05 | 2.94104259436783e-21 | 310915142889.897 |
| 87 | rs9271576 | 6 | 32590612 | 0.303181 | T | C | -0.001110119 | 6.95253105196754e-05 | 2.16553365588309e-57 | 310915142889.897 |
| 88 | rs9271607 | 6 | 32591566 | 0.311133 | A | G | -0.000908858 | 6.88765404387793e-05 | 9.31858771090367e-40 | 310915142889.897 |
| 89 | rs35242582 | 6 | 32600057 | 0.243539 | A | G | 0.00104790854245288 | 7.74354082252191e-05 | 1.00300272007747e-41 | 310915142889.897 |
| 90 | rs17211580 | 6 | 32602754 | 0.265408 | G | A | 0.000843785358938419 | 7.80096353407525e-05 | 2.87785436166402e-27 | 310915142889.897 |
| 91 | rs9272319 | 6 | 32604108 | 0.327038 | T | C | -0.000518816 | 6.90869992420496e-05 | 5.93053737670539e-14 | 310915142889.897 |
| 92 | rs9273044 | 6 | 32611971 | 0.271372 | G | C | -0.000752382 | 8.01436817522957e-05 | 6.11977783600754e-21 | 310915142889.897 |
| 93 | rs9273078 | 6 | 32612309 | 0.415507 | T | A | -0.001536485 | 7.41504978026921e-05 | 2.23193415238992e-95 | 310915142889.897 |
| 94 | rs9273079 | 6 | 32612326 | 0.187873 | C | T | -0.001281916 | 8.11229404709805e-05 | 3.00732835083782e-56 | 310915142889.897 |
| 95 | rs9273082 | 6 | 32612339 | 0.425447 | C | T | -0.00052667 | 7.27019994207555e-05 | 4.34890452421563e-13 | 310915142889.897 |
| 96 | rs9273084 | 6 | 32612396 | 0.491054 | T | C | -0.001001919 | 6.78044000162976e-05 | 2.07372082988589e-49 | 310915142889.897 |
| 97 | rs6927022 | 6 | 32612397 | 0.392644 | A | G | -0.001083229 | 6.7066804283923e-05 | 1.10833275283518e-58 | 310915142889.897 |
| 98 | rs17612562 | 6 | 32615446 | 0.456262 | T | G | -0.000726232 | 7.92544006541152e-05 | 5.03346968683454e-20 | 310915142889.897 |
| 99 | rs17612583 | 6 | 32615470 | 0.49006 | G | A | -0.001384838 | 7.40130112732677e-05 | 4.04717769113755e-78 | 310915142889.897 |
| 100 | rs17843580 | 6 | 32615551 | 0.493042 | A | G | -0.001180493 | 6.81672918607691e-05 | 3.46545979600594e-67 | 310915142889.897 |
| 101 | rs17612590 | 6 | 32615578 | 0.437376 | G | A | -0.000670121 | 6.79934303542362e-05 | 6.47824856130726e-23 | 310915142889.897 |
| 102 | rs17843583 | 6 | 32615652 | 0.461233 | A | C | -0.001449374 | 6.78576827502688e-05 | 3.21472267312713e-101 | 310915142889.897 |
| 103 | rs17612633 | 6 | 32615710 | 0.4334 | G | C | -0.001088017 | 6.75743459733006e-05 | 2.50851339674909e-58 | 310915142889.897 |
| 104 | rs17843604 | 6 | 32620283 | 0.466203 | C | T | -0.000593813 | 6.71917691231312e-05 | 9.78040778500853e-19 | 310915142889.897 |
| 105 | rs17612781 | 6 | 32620311 | 0.493042 | T | C | -0.00076902 | 6.75833392472865e-05 | 5.33013901233379e-30 | 310915142889.897 |
| 106 | rs17612802 | 6 | 32620359 | 0.474155 | C | T | -0.001297048 | 6.82119142592497e-05 | 1.28205899082652e-80 | 310915142889.897 |
| 107 | rs9273329 | 6 | 32623242 | 0.439364 | G | A | -0.001155215 | 6.76504407209344e-05 | 2.23024479417147e-65 | 310915142889.897 |
| 108 | rs9273335 | 6 | 32623348 | 0.410537 | A | G | -0.000925214 | 6.89846106962936e-05 | 5.15086640519367e-41 | 310915142889.897 |
| 109 | rs9273338 | 6 | 32623393 | 0.413519 | A | T | -0.000983881 | 7.14519559059167e-05 | 3.87152309691392e-43 | 310915142889.897 |
| 110 | rs9273363 | 6 | 32626272 | 0.262425 | C | A | -0.00050159 | 7.14371034367633e-05 | 2.19612458027724e-12 | 310915142889.897 |
| 111 | rs9273400 | 6 | 32627128 | 0.408549 | C | T | -0.001500004 | 7.05651770821554e-05 | 2.83962099791833e-100 | 310915142889.897 |
| 112 | rs9273404 | 6 | 32627160 | 0.460239 | A | G | -0.000726367 | 7.18230891700766e-05 | 4.82411812666968e-24 | 310915142889.897 |
| 113 | rs9273419 | 6 | 32627365 | 0.475149 | T | C | -0.001256613 | 8.32189911391534e-05 | 1.61747946726226e-51 | 310915142889.897 |
| 114 | rs1049225 | 6 | 32627747 | 0.209742 | A | G | -0.000904175 | 7.78714464786247e-05 | 3.61814533784096e-31 | 310915142889.897 |
| 115 | rs1049213 | 6 | 32627773 | 0.233598 | A | G | -0.000851611 | 7.67890639494636e-05 | 1.3987743052293e-28 | 310915142889.897 |
| 116 | rs28746843 | 6 | 32634137 | 0.208748 | A | C | -0.000804291 | 8.74311650104875e-05 | 3.60846041716307e-20 | 310915142889.897 |
| 117 | rs3891175 | 6 | 32634467 | 0.170974 | C | T | -0.000561453 | 7.53068502356605e-05 | 8.95051640930494e-14 | 310915142889.897 |
| 118 | rs3828789 | 6 | 32635754 | 0.409543 | G | T | -0.001433155 | 6.73812357979957e-05 | 2.18318294832877e-100 | 310915142889.897 |
| 119 | rs3828790 | 6 | 32635809 | 0.404573 | T | C | -0.000829215 | 6.71659391616729e-05 | 5.13416491623003e-35 | 310915142889.897 |
| 120 | rs9274622 | 6 | 32635990 | 0.37674 | T | C | -0.000514945 | 6.6700205920889e-05 | 1.16066345970768e-14 | 310915142889.897 |
| 121 | rs9274623 | 6 | 32635998 | 0.202783 | G | T | -0.000583599 | 7.67172045469081e-05 | 2.80210974368934e-14 | 310915142889.897 |
| 122 | rs9274626 | 6 | 32636040 | 0.217694 | T | C | -0.000958563 | 7.24668842405238e-05 | 6.0792823453236e-40 | 310915142889.897 |
| 123 | rs3828805 | 6 | 32636120 | 0.217694 | T | C | -0.001019805 | 7.60338380684474e-05 | 5.10726084597151e-41 | 310915142889.897 |
| 124 | rs9275144 | 6 | 32651218 | 0.270378 | T | A | -0.000860476 | 7.76664925225596e-05 | 1.58422055936124e-28 | 310915142889.897 |
| 125 | rs3021061 | 6 | 32651839 | 0.264414 | C | G | -0.000812848 | 7.7692036247465e-05 | 1.28514655949832e-25 | 310915142889.897 |
| 126 | rs3021059 | 6 | 32652344 | 0.287276 | C | T | -0.000788738 | 7.75749558832834e-05 | 2.77103687236216e-24 | 310915142889.897 |
| 127 | rs3021058 | 6 | 32652359 | 0.486083 | A | C | -0.000537365 | 6.68216071575366e-05 | 8.85423169948663e-16 | 310915142889.897 |
| 128 | rs2856691 | 6 | 32653435 | 0.271372 | G | A | -0.000816707 | 7.62163627952684e-05 | 8.59647104690766e-27 | 310915142889.897 |
| 129 | rs9275169 | 6 | 32653721 | 0.469185 | T | A | -0.000483675 | 6.56077218522674e-05 | 1.67798564951365e-13 | 310915142889.897 |
| 130 | rs9275172 | 6 | 32653997 | 0.459245 | A | G | -0.000528941 | 6.55626319803578e-05 | 7.1621964669305e-16 | 310915142889.897 |
| 131 | rs3135190 | 6 | 32667946 | 0.205765 | T | G | -0.000698384 | 7.80666451172643e-05 | 3.68599682928697e-19 | 310915142889.897 |
| 132 | rs2647045 | 6 | 32668100 | 0.276342 | G | A | -0.000600007 | 7.64698746158576e-05 | 4.28446900150469e-15 | 310915142889.897 |
| 133 | rs3104407 | 6 | 32682452 | 0.484095 | G | A | -0.001084048 | 6.80428666072357e-05 | 3.8096267974525e-57 | 310915142889.897 |
| 134 | rs9501664 | 6 | 32768488 | 0.337972 | C | A | 0.000529433594984251 | 7.19721800368851e-05 | 1.89380064554018e-13 | 310915142889.897 |
| 135 | rs2621358 | 6 | 32770808 | 0.337972 | C | T | 0.000740663362320278 | 7.2472351759385e-05 | 1.61434439415866e-24 | 310915142889.897 |
| 136 | rs241444 | 6 | 32797109 | 0.276342 | G | A | 0.00065986390788085 | 7.5942940042042e-05 | 3.65825839676115e-18 | 310915142889.897 |
| 137 | rs2031901 | 6 | 39670040 | 0.44831 | G | A | 0.000417499628432578 | 5.80445515739102e-05 | 6.35017146006919e-13 | 310915142889.897 |
| 138 | rs12669390 | 7 | 9097584 | 0.478131 | T | G | -0.00051278 | 5.47666248182093e-05 | 7.74986401234984e-21 | 310915142889.897 |
| 139 | rs4262274 | 7 | 129639781 | 0.377734 | C | A | 0.000469170001861564 | 6.42521020963415e-05 | 2.83483469862834e-13 | 310915142889.897 |
| 140 | rs2014976 | 9 | 4681536 | 0.233598 | C | T | 0.000538097991138033 | 7.33860046949972e-05 | 2.26010814766022e-13 | 310915142889.897 |
| 141 | rs34270971 | 9 | 34214758 | 0.262425 | T | C | -0.000453239 | 6.45203634183532e-05 | 2.14471326919865e-12 | 310915142889.897 |
| 142 | rs2122777 | 11 | 103372348 | 0.284294 | T | C | 0.000483103535762331 | 6.32915014286569e-05 | 2.29366792615983e-14 | 310915142889.897 |
| 143 | rs10774625 | 12 | 111910219 | 0.477137 | A | G | 0.000512508908044023 | 6.51739557366037e-05 | 3.72928538206313e-15 | 310915142889.897 |
| 144 | rs2554379 | 13 | 103738102 | 0.250497 | T | C | 0.000638321372113107 | 6.53112948551977e-05 | 1.46276780963434e-22 | 310915142889.897 |
| 145 | rs2683100 | 15 | 36146982 | 0.265408 | C | A | -0.00051765 | 6.63896250186713e-05 | 6.33235650060855e-15 | 310915142889.897 |
| 146 | rs1428806 | 16 | 9420635 | 0.429423 | T | C | 0.000470836406032438 | 5.64405844218282e-05 | 7.29454407325476e-17 | 310915142889.897 |
| 147 | rs16948981 | 17 | 48457251 | 0.230616 | G | A | 0.000729304213271395 | 7.13908992558469e-05 | 1.68749879157113e-24 | 310915142889.897 |
| 148 | rs8090533 | 18 | 19690316 | 0.375746 | T | C | 0.000581481239304729 | 6.68071620290545e-05 | 3.20738107994066e-18 | 310915142889.897 |
| 149 | rs10418694 | 19 | 35116023 | 0.395626 | A | C | -0.000580639 | 6.28110603579498e-05 | 2.36960617073372e-20 | 310915142889.897 |
| 150 | rs7510260 | 21 | 34196195 | 0.486083 | G | A | 0.000501505481061087 | 6.1406481802792e-05 | 3.16206477533902e-16 | 310915142889.897 |

**Supplementary Table 4.** Risk genomic locus identified by Genomic-SEM

| **Number** | **Genomic locus** | **SNP** | **CHR** | **Positon** | ***P* value** |
| --- | --- | --- | --- | --- | --- |
| 1 | 1:62452749:C:G | rs6671315 | 1 | 62452749 | 2.15569638349e-12 |
| 2 | 2:42460163:C:G | rs11124863 | 2 | 42460163 | 5.02997075237e-30 |
| 3 | 2:61070652:A:G | rs1432297 | 2 | 61070652 | 1.47258992067e-18 |
| 4 | 2:140902383:A:G | rs2950611 | 2 | 140902383 | 5.09155901644e-13 |
| 5 | 3:42892048:A:G | rs2099022 | 3 | 42892048 | 5.37601498693e-10 |
| 6 | 3:165715990:A:C | rs35865016 | 3 | 165715990 | 2.82367703825e-14 |
| 7 | 4:5942417:C:G | rs6842718 | 4 | 5942417 | 3.04822724552e-15 |
| 8 | 4:29409982:A:G | rs260120 | 4 | 29409982 | 1.0278918637e-12 |
| 9 | 4:76886398:A:G | rs11097189 | 4 | 76886398 | 5.47102709161e-14 |
| 10 | 4:88577127:C:T | rs7678998 | 4 | 88577127 | 5.30449956132e-11 |
| 11 | 4:91669114:A:C | rs58817164 | 4 | 91669114 | 9.35494420482e-19 |
| 12 | 4:122600522:A:G | rs11098638 | 4 | 122600522 | 1e-200 |
| 13 | 4:184636601:A:G | rs11735434 | 4 | 184636601 | 4.88795237311e-08 |
| 14 | 6:29042672:A:G | rs3131084 | 6 | 29042672 | 3.35763925869e-09 |
| 15 | 6:32615652:A:C | rs17843583 | 6 | 32615652 | 3.21472267313e-101 |
| 16 | 6:33124972:A:G | rs3130160 | 6 | 33124972 | 6.44715713499e-09 |
| 17 | 6:39670040:A:G | rs2031901 | 6 | 39670040 | 6.35017146007e-13 |
| 18 | 6:132814037:A:G | rs2788942 | 6 | 132814037 | 5.52504404704e-09 |
| 19 | 6:161916480:C:T | rs9458289 | 6 | 161916480 | 6.92429384837e-09 |
| 20 | 7:9097584:G:T | rs12669390 | 7 | 9097584 | 7.74986401235e-21 |
| 21 | 7:129639781:A:C | rs4262274 | 7 | 129639781 | 2.83483469863e-13 |
| 22 | 8:11737024:A:C | rs74353793 | 8 | 11737024 | 5.94130900233e-09 |
| 23 | 9:4681536:C:T | rs2014976 | 9 | 4681536 | 2.26010814766e-13 |
| 24 | 9:34214758:C:T | rs34270971 | 9 | 34214758 | 2.1447132692e-12 |
| 25 | 9:118339739:C:T | rs2157668 | 9 | 118339739 | 4.53569331462e-08 |
| 26 | 10:10765915:G:T | rs10737043 | 10 | 10765915 | 2.25270113143e-08 |
| 27 | 10:119692579:G:T | rs7073284 | 10 | 119692579 | 1.38515490636e-11 |
| 28 | 11:103372348:C:T | rs2122777 | 11 | 103372348 | 2.29366792616e-14 |
| 29 | 12:33073504:A:G | rs10844385 | 12 | 33073504 | 4.56630495715e-10 |
| 30 | 12:54197266:A:G | rs12830815 | 12 | 54197266 | 1.02260454586e-10 |
| 31 | 12:111910219:A:G | rs10774625 | 12 | 111910219 | 3.72928538206e-15 |
| 32 | 13:55251524:C:T | rs9527250 | 13 | 55251524 | 1.12408970639e-10 |
| 33 | 13:103738102:C:T | rs2554379 | 13 | 103738102 | 1.46276780963e-22 |
| 34 | 14:31392606:C:T | rs7153028 | 14 | 31392606 | 1.63389327855e-10 |
| 35 | 15:36146982:A:C | rs2683100 | 15 | 36146982 | 6.33235650061e-15 |
| 36 | 15:68182254:C:T | rs28445794 | 15 | 68182254 | 3.20433586056e-08 |
| 37 | 16:9420635:C:T | rs1428806 | 16 | 9420635 | 7.29454407325e-17 |
| 38 | 16:57353765:C:T | rs12924770 | 16 | 57353765 | 4.41963963487e-10 |
| 39 | 17:48457251:A:G | rs16948981 | 17 | 48457251 | 1.68749879157e-24 |
| 40 | 18:19690316:C:T | rs8090533 | 18 | 19690316 | 3.20738107994e-18 |
| 41 | 19:35116023:A:C | rs10418694 | 19 | 35116023 | 2.36960617073e-20 |
| 42 | 20:54207397:A:T | rs6069350 | 20 | 54207397 | 5.53863694339e-11 |
| 43 | 21:34196195:A:G | rs7510260 | 21 | 34196195 | 3.16206477534e-16 |

**Supplementary Table 5.** Potential related genomic loci identified by Genomic-SEM

| **Number** | **Gene** | **CHR** | **Positon** | ***P* value** | **FDR** |
| --- | --- | --- | --- | --- | --- |
| 1 | ENSG00000213760 | 6 | 62452749 | 5e-10 | 1.065875e-06 |
| 2 | ENSG00000204498 | 6 | 42460163 | 3.3986e-10 | 1.065875e-06 |
| 3 | ENSG00000204314 | 6 | 61070652 | 7.4938e-08 | 0.000106499387666667 |
| 4 | ENSG00000221988 | 6 | 140902383 | 8.0953e-10 | 1.53396940222222e-06 |
| 5 | ENSG00000258388 | 6 | 42892048 | 5.969e-09 | 9.25412054545455e-06 |
| 6 | ENSG00000241404 | 6 | 165715990 | 5e-10 | 1.065875e-06 |
| 7 | ENSG00000204310 | 6 | 5942417 | 1.4889e-11 | 8.4639002e-08 |
| 8 | ENSG00000204308 | 6 | 29409982 | 4.0487e-13 | 3.45232649e-09 |
| 9 | ENSG00000204304 | 6 | 76886398 | 2.1397e-09 | 3.64904438e-06 |
| 10 | ENSG00000204301 | 6 | 88577127 | 3.0025e-06 | 0.00365747392857143 |
| 11 | ENSG00000204290 | 6 | 91669114 | 2.9368e-10 | 1.065875e-06 |
| 12 | ENSG00000196735 | 6 | 122600522 | 2.8921e-14 | 4.93218734e-10 |
| 13 | ENSG00000179344 | 6 | 184636601 | 5e-10 | 1.065875e-06 |
| 14 | ENSG00000154920 | 17 | 29042672 | 3.7064e-07 | 0.000486222658461538 |

**Supplementary Table 6.** Lead SNP variants identified by Genomic-SEM

| **Number** | **Lead SNP** | **CHR** | **Positon** | **Independent SNPs** | **Location** | **Beta** | ***P* value** |
| --- | --- | --- | --- | --- | --- | --- | --- |
| 1 | rs6671315 | 1 | 62452749 | rs10910093 | intronic | 0.000535363522691 | 2.15569638349e-12 |
| 2 | rs11124863 | 2 | 42460163 | rs6577488, rs12735687, rs7530406, rs11588961 | intronic | -0.000803544277139 | 5.02997075237e-30 |
| 3 | rs1432297 | 2 | 61070652 | rs186621414 | ncRNA intronic | 0.000537083272276 | 1.47258992067e-18 |
| 4 | rs2950611 | 2 | 140902383 | rs16830141 | intergenic | 0.000506494560935 | 5.09155901644e-13 |
| 5 | rs2099022 | 3 | 42892048 | rs192597957 | ncRNA intronic | -0.000434390897658 | 5.37601498693e-10 |
| 6 | rs35865016 | 3 | 165715990 | rs114695117 | intergenic | -0.000454928049643 | 2.82367703825e-14 |
| 7 | rs6842718 | 4 | 5942417 | rs149030372 | intergenic | -0.000416430076436 | 3.04822724552e-15 |
| 8 | rs260120 | 4 | 29409982 | rs4284267 | downstream | 0.00049010059458 | 1.0278918637e-12 |
| 9 | rs11097189 | 4 | 76886398 | rs10800313, rs1723015 | intronic | 0.000503923536553 | 5.47102709161e-14 |
| 10 | rs7678998 | 4 | 88577127 | rs116697924 | ncRNA intronic | -0.00044767150451 | 5.30449956132e-11 |
| 11 | rs58817164 | 4 | 91669114 | rs72756399 | intronic | 0.000518021537419 | 9.35494420482e-19 |
| 12 | rs11098638 | 4 | 122600522 | rs74538397 | intronic | -0.00248603848444 | 1e-200 |
| 13 | rs11735434 | 4 | 184636601 | rs16858573 | intergenic | -0.000391930133545 | 4.88795237311e-08 |
| 14 | rs3131084 | 6 | 29042672 | rs10930919 | intergenic | -0.000388164238447 | 3.35763925869e-09 |
| 15 | rs2394224 | 6 | 29837703 | rs6434942, rs11679484, rs34388051, rs1429419, rs11901138, rs6434934 | intergenic | -0.000474713753842 | 1.07184320218e-11 |
| 16 | rs3095330 | 6 | 30693633 | rs9808132 | downstream | -0.000889842766132 | 1.00357954315e-29 |
| 17 | rs130065 | 6 | 31122500 | rs34077392, rs35359354, rs4425108, rs28433874, rs71351116 | exonic | -0.000884342371979 | 1.71097451262e-30 |
| 18 | rs3099837 | 6 | 31417463 | rs12473044, rs28433874, rs35359354, rs4425108, rs4443030 | ncRNA intronic | -0.000963009529783 | 2.73023013521e-44 |
| 19 | rs3134932 | 6 | 32186147 | rs11927929 | intronic | -0.000644043551425 | 1.20237229599e-21 |
| 20 | rs715299 | 6 | 32189841 | rs9288958 | intronic | -0.00101973209604 | 2.27795331245e-48 |
| 21 | rs3117133 | 6 | 32313653 | rs1709 | intronic | 0.000649044992934 | 8.42794351416e-27 |
| 22 | rs13194770 | 6 | 32451570 | rs6768827 | intergenic | -0.000410477881981 | 3.51841683745e-10 |
| 23 | rs17843583 | 6 | 32615652 | rs112174832 | intergenic | -0.00144937372993 | 3.21472267313e-101 |
| 24 | rs2621358 | 6 | 32770808 | rs11736191 | intergenic | 0.00074066336232 | 1.61434439416e-24 |
| 25 | rs3130160 | 6 | 33124972 | rs6824105, rs75983064, rs5743810, rs34089598, rs10018679, rs11727203, rs10008032 | intergenic | 0.000418635889504 | 6.44715713499e-09 |
| 26 | rs2031901 | 6 | 39670040 | rs7679798, rs5743586, rs34293100, rs12504153, rs6824924, rs11466642 | intronic | 0.000417499628433 | 6.35017146007e-13 |
| 27 | rs2788942 | 6 | 132814037 | rs6843572, rs9996722, rs11727203, rs4286521, rs5743810 | intronic | -0.000329038403089 | 5.52504404704e-09 |
| 28 | rs9458289 | 6 | 161916480 | rs112282407 | intronic | -0.00036683883951 | 6.92429384837e-09 |
| 29 | rs12669390 | 7 | 9097584 | rs112040197, rs75983064 | intergenic | -0.000512780321409 | 7.74986401235e-21 |
| 30 | rs4262274 | 7 | 129639781 | rs13113644, rs732056 | ncRNA intronic | 0.000469170001862 | 2.83483469863e-13 |
| 31 | rs74353793 | 8 | 11737024 | rs145630320 | intergenic | 0.000400185069039 | 5.94130900233e-09 |
| 32 | rs2014976 | 9 | 4681536 | rs115458629 | intronic | 0.000538097991138 | 2.26010814766e-13 |
| 33 | rs34270971 | 9 | 34214758 | rs76058999 | intronic | -0.000453238521803 | 2.1447132692e-12 |
| 34 | rs2157668 | 9 | 118339739 | rs72774811, rs244407, rs72774818, rs11241068, rs72771461, rs6886061, rs381661, rs6873681 | intergenic | 0.000346556954335 | 4.53569331462e-08 |
| 35 | rs10737043 | 10 | 10765915 | rs62379371 | intergenic | -0.000403383835197 | 2.25270113143e-08 |
| 36 | rs7073284 | 10 | 119692579 | rs2961918 | intergenic | 0.00045752644225 | 1.38515490636e-11 |
| 37 | rs2122777 | 11 | 103372348 | rs75320366 | intergenic | 0.000483103535762 | 2.29366792616e-14 |
| 38 | rs10844385 | 12 | 33073504 | rs1150658, rs2070642 | intergenic | -0.000452216815995 | 4.56630495715e-10 |
| 39 | rs12830815 | 12 | 54197266 | rs145982144 | intergenic | -0.000365550374546 | 1.02260454586e-10 |
| 40 | rs10774625 | 12 | 111910219 | rs3025651, rs116611655 | intronic | 0.000512508908044 | 3.72928538206e-15 |
| 41 | rs9527250 | 13 | 55251524 | rs3131857 | intergenic | -0.000407670901363 | 1.12408970639e-10 |
| 42 | rs2554379 | 13 | 103738102 | rs62407901 | intergenic | 0.000638321372113 | 1.46276780963e-22 |
| 43 | rs7153028 | 14 | 31392606 | rs7750412 | intronic | 0.000419023424662 | 1.63389327855e-10 |
| 44 | rs2683100 | 15 | 36146982 | rs143392496 | ncRNA intronic | -0.000517649519536 | 6.33235650061e-15 |
| 45 | rs28445794 | 15 | 68182254 | rs142883099 | intergenic | -0.000380956518503 | 3.20433586056e-08 |
| 46 | rs1428806 | 16 | 9420635 | rs739751 | intergenic | 0.000470836406032 | 7.29454407325e-17 |
| 47 | rs12924770 | 16 | 57353765 | rs2023789, rs79721266, rs28423374 | intergenic | 0.000397935228988 | 4.41963963487e-10 |
| 48 | rs16948981 | 17 | 48457251 | rs186735625, rs6971086, rs2015027 | intronic | 0.000729304213271 | 1.68749879157e-24 |
| 49 | rs8090533 | 18 | 19690316 | rs4562243 | intergenic | 0.000581481239305 | 3.20738107994e-18 |
| 50 | rs10418694 | 19 | 35116023 | rs3918226 | intronic | -0.000580639318385 | 2.36960617073e-20 |
| 51 | rs6069350 | 20 | 54207397 | rs11783015 | intergenic | 0.000397308322331 | 5.53863694339e-11 |
| 52 | rs7510260 | 21 | 34196195 | rs66632892 | intronic | 0.000501505481061 | 3.16206477534e-16 |

**Supplementary Table 7.** Novel SNP variants identified by Genomic-SEM

| **Number** | **Novel SNP** | **CHR** | **Positon** | **Nearest Gene** | **Effect allele** | **Other allele** | **Location** | **MAF** | **Beta** | **SE** | ***P* value** |
| --- | --- | --- | --- | --- | --- | --- | --- | --- | --- | --- | --- |
| 1 | rs3131084 | 6 | 29042672 | SAR1P1 | G | A | intergenic | 0.488072 | -0.000388164238447 | 6.56449896435e-05 | 3.35763925869e-09 |
| 2 | rs2394224 | 6 | 29837703 | HCG4P7 | A | G | intergenic | 0.356859 | -0.000474713753842 | 6.98467001718e-05 | 1.07184320218e-11 |
| 3 | rs3095330 | 6 | 30693633 | TUBB | A | G | downstream | 0.181909 | -0.000889842766132 | 7.85835724934e-05 | 1.00357954315e-29 |
| 4 | rs130065 | 6 | 31122500 | CCHCR1 | G | A | exonic | 0.191849 | -0.000884342371979 | 7.70498810899e-05 | 1.71097451262e-30 |
| 5 | rs3099837 | 6 | 31417463 | HCP5 | G | A | ncRNA intronic | 0.249503 | -0.000963009529783 | 6.8982961117e-05 | 2.73023013521e-44 |
| 6 | rs3134932 | 6 | 32186147 | NOTCH4 | C | A | intronic | 0.377734 | -0.000644043551425 | 6.73837806921e-05 | 1.20237229599e-21 |
| 7 | rs715299 | 6 | 32189841 | NOTCH4 | T | G | intronic | 0.265408 | -0.00101973209604 | 6.97764827041e-05 | 2.27795331245e-48 |
| 8 | rs3117133 | 6 | 32313653 | C6orf10 | G | C | intronic | 0.223658 | 0.000649044992934 | 6.05595487439e-05 | 8.42794351416e-27 |
| 9 | rs13194770 | 6 | 32451570 | HLA-DRB9 | C | T | intergenic | 0.44334 | -0.000410477881981 | 6.54250123035e-05 | 3.51841683745e-10 |
| 10 | rs17843583 | 6 | 32615652 | HLA-DQA1 | A | C | intergenic | 0.461233 | -0.00144937372993 | 6.78576827503e-05 | 3.21472267313e-101 |
| 11 | rs2621358 | 6 | 32770808 | HLA-DOB | C | T | intergenic | 0.337972 | 0.00074066336232 | 7.24723517594e-05 | 1.61434439416e-24 |
| 12 | rs3130160 | 6 | 33124972 | COL11A2 | A | G | intergenic | 0.272366 | 0.000418635889504 | 7.21198436737e-05 | 6.44715713499e-09 |
| 13 | rs10774625 | 12 | 111910219 | ATXN2 | A | G | intronic | 0.477137 | 0.000512508908044 | 6.51739557366e-05 | 3.72928538206e-15 |

**Supplementary Table 8.** Significant Genomic Loci identified by SuSIE and FINEMAP

| **Number** | **SNP** | **CHR** | **Positon** | **Nearest Gene** | **Effect allele** | **Other allele** | **MAF** | **Beta** | **SE** | ***P* value** |
| --- | --- | --- | --- | --- | --- | --- | --- | --- | --- | --- |
| 1 | rs3134608 | 6 | 32117971 | C6orf10 | T | G | 0.204771 | -0.000502990455392443 | 7.69800066339515e-05 | 6.40187638823043e-11 |
| 2 | rs3134952 | 6 | 32113571 | C6orf10 | A | T | 0.204771 | -0.000360438995983838 | 7.67676109810364e-05 | 2.66350939227694e-06 |
| 3 | rs3763307 | 6 | 32374622 | HLA-DQA1 | A | T | 0.27336 | -0.00101784260752969 | 7.17788863530135e-05 | 1.21411943928203e-45 |
| 4 | rs2076524 | 6 | 32370684 | HLA-DQA1 | A | G | 0.27336 | -0.000303303241325288 | 7.02144584634477e-05 | 1.56263183071738e-05 |

**Supplementary Table 9.** Potential MGLSC associated gene identified by FOCUS

| **Number** | **Block** | **Gene** | **CHR** | **Start** | **End** | **Type** | **Z score** | **Eqtl file** |
| --- | --- | --- | --- | --- | --- | --- | --- | --- |
| 1 | 6:30798168-6:31571218 | CLIC1 | 6 | 31739762 | 31739763 | protein_coding | -6.87 | sCCA3.db |
| 2 | 17:47517400-17:49898957 | ENSG00000279792.1 | 17 | 50365231 | 50365232 | TEC | 9.54 | sCCA2.db |
| 3 | 17:47517400-17:49898957 | XYLT2 | 17 | 50346091 | 50346092 | protein_coding | -0.658 | sCCA2.db |
| 4 | 6:30798168-6:31571218 | DDAH2 | 6 | 31730579 | 31730580 | protein_coding | 8.8 | sCCA1.db |
| 5 | 17:47517400-17:49898957 | COL1A1 | 17 | 50201631 | 50201632 | protein_coding | 0.954 | sCCA2.db |
| 6 | 6:30798168-6:31571218 | MSH5 | 6 | 31739947 | 31739948 | protein_coding | -6.27 | sCCA2.db |
| 7 | 6:30798168-6:31571218 | NFKBIL1 | 6 | 31546869 | 31546870 | protein_coding | 3.38 | sCCA2.db |
| 8 | 6:30798168-6:31571218 | C4B | 6 | 32014761 | 32014762 | protein_coding | -0.662 | sCCA3.db |
| 9 | 6:31571218-6:32682664 | HLA-DPA1 | 6 | 33073771 | 33073772 | protein_coding | 8.43 | sCCA1.db |
| 10 | 6:31571218-6:32682664 | HLA-DPA1 | 6 | 33073771 | 33073772 | protein_coding | -8.43 | sCCA3.db |
| 11 | 6:32682664-6:33236497 | HLA-DPA1 | 6 | 33073771 | 33073772 | protein_coding | -4.43 | sCCA2.db |
| 12 | 8:11278998-8:13491775 | NULL | 8 | NA | NA | NULL | 0 | sCCA1.db |
| 13 | 3:165055469-3:167117429 | NULL | 3 | NA | NA | NULL | 0 | sCCA2.db |
| 14 | 3:165055469-3:167117429 | NULL | 3 | NA | NA | NULL | 0 | sCCA1.db |
| 15 | 12:53039004-12:54778823 | NULL | 12 | NA | NA | NULL | 0 | sCCA1.db |
| 16 | 12:53039004-12:54778823 | NULL | 12 | NA | NA | NULL | 0 | sCCA2.db |
| 17 | 8:11278998-8:13491775 | NULL | 8 | NA | NA | NULL | 0 | sCCA2.db |
| 18 | 12:53039004-12:54778823 | NULL | 12 | NA | NA | NULL | 0 | sCCA3.db |
| 19 | 8:11278998-8:13491775 | NULL | 8 | NA | NA | NULL | 0 | sCCA3.db |
| 20 | 6:31571218-6:32682664 | HLA-DQA1 | 6 | 32628178 | 32628179 | protein_coding | -7.12 | sCCA2.db |
| 21 | 19:34262952-19:36469295 | NULL | 19 | NA | NA | NULL | 0 | sCCA1.db |
| 22 | 6:30798168-6:31571218 | DDR1 | 6 | 30876420 | 30876421 | protein_coding | 0.000953 | sCCA1.db |
| 23 | 19:34262952-19:36469295 | NULL | 19 | NA | NA | NULL | 0 | sCCA2.db |
| 24 | 11:101331121-11:103959636 | NULL | 11 | NA | NA | NULL | 0 | sCCA3.db |
| 25 | 12:110336719-12:113263518 | NULL | 12 | NA | NA | NULL | 0 | sCCA3.db |
| 26 | 11:101331121-11:103959636 | NULL | 11 | NA | NA | NULL | 0 | sCCA2.db |
| 27 | 19:34262952-19:36469295 | NULL | 19 | NA | NA | NULL | 0 | sCCA3.db |
| 28 | 12:110336719-12:113263518 | NULL | 12 | NA | NA | NULL | 0 | sCCA1.db |
| 29 | 14:29972145-14:32383265 | NULL | 14 | NA | NA | NULL | 0 | sCCA1.db |
| 30 | 17:47517400-17:49898957 | NULL | 17 | NA | NA | NULL | 0 | sCCA3.db |
| 31 | 2:60292000-2:62429044 | NULL | 2 | NA | NA | NULL | 0 | sCCA2.db |
| 32 | 4:183371275-4:184929455 | NULL | 4 | NA | NA | NULL | 0 | sCCA1.db |
| 33 | 4:74592390-4:77130707 | NULL | 4 | NA | NA | NULL | 0 | sCCA2.db |
| 34 | 18:14440799-18:20649472 | NULL | 18 | NA | NA | NULL | 0 | sCCA3.db |
| 35 | 14:29972145-14:32383265 | NULL | 14 | NA | NA | NULL | 0 | sCCA3.db |
| 36 | 12:110336719-12:113263518 | NULL | 12 | NA | NA | NULL | 0 | sCCA2.db |
| 37 | 11:101331121-11:103959636 | NULL | 11 | NA | NA | NULL | 0 | sCCA1.db |
| 38 | 9:117921960-9:121321537 | NULL | 9 | NA | NA | NULL | 0 | sCCA1.db |
| 39 | 14:29972145-14:32383265 | NULL | 14 | NA | NA | NULL | 0 | sCCA2.db |
| 40 | 15:35083251-15:37456502 | NULL | 15 | NA | NA | NULL | 0 | sCCA3.db |
| 41 | 4:74592390-4:77130707 | NULL | 4 | NA | NA | NULL | 0 | sCCA3.db |
| 42 | 18:14440799-18:20649472 | NULL | 18 | NA | NA | NULL | 0 | sCCA2.db |
| 43 | 16:55903774-16:57664330 | NULL | 16 | NA | NA | NULL | 0 | sCCA2.db |
| 44 | 4:183371275-4:184929455 | NULL | 4 | NA | NA | NULL | 0 | sCCA2.db |
| 45 | 15:35083251-15:37456502 | NULL | 15 | NA | NA | NULL | 0 | sCCA1.db |
| 46 | 3:42540002-3:45165153 | NULL | 3 | NA | NA | NULL | 0 | sCCA1.db |
| 47 | 2:60292000-2:62429044 | NULL | 2 | NA | NA | NULL | 0 | sCCA1.db |
| 48 | 4:183371275-4:184929455 | NULL | 4 | NA | NA | NULL | 0 | sCCA3.db |
| 49 | 2:60292000-2:62429044 | NULL | 2 | NA | NA | NULL | 0 | sCCA3.db |
| 50 | 18:14440799-18:20649472 | NULL | 18 | NA | NA | NULL | 0 | sCCA1.db |
| 51 | 15:35083251-15:37456502 | NULL | 15 | NA | NA | NULL | 0 | sCCA2.db |
| 52 | 9:117921960-9:121321537 | NULL | 9 | NA | NA | NULL | 0 | sCCA2.db |
| 53 | 3:42540002-3:45165153 | NULL | 3 | NA | NA | NULL | 0 | sCCA2.db |
| 54 | 9:33578334-9:34642243 | NULL | 9 | NA | NA | NULL | 0 | sCCA1.db |
| 55 | 6:132765669-6:134244243 | NULL | 6 | NA | NA | NULL | 0 | sCCA2.db |
| 56 | 4:74592390-4:77130707 | NULL | 4 | NA | NA | NULL | 0 | sCCA1.db |
| 57 | 7:128778386-7:130422414 | NULL | 7 | NA | NA | NULL | 0 | sCCA3.db |
| 58 | 3:42540002-3:45165153 | NULL | 3 | NA | NA | NULL | 0 | sCCA3.db |
| 59 | 16:55903774-16:57664330 | NULL | 16 | NA | NA | NULL | 0 | sCCA3.db |
| 60 | 6:28917608-6:29737971 | NULL | 6 | NA | NA | NULL | 0 | sCCA2.db |
| 61 | 9:117921960-9:121321537 | NULL | 9 | NA | NA | NULL | 0 | sCCA3.db |
| 62 | 16:8338519-16:9799419 | NULL | 16 | NA | NA | NULL | 0 | sCCA2.db |
| 63 | 16:8338519-16:9799419 | NULL | 16 | NA | NA | NULL | 0 | sCCA3.db |
| 64 | 4:120392684-4:122657987 | NULL | 4 | NA | NA | NULL | 0 | sCCA1.db |
| 65 | 9:33578334-9:34642243 | NULL | 9 | NA | NA | NULL | 0 | sCCA2.db |
| 66 | 16:55903774-16:57664330 | NULL | 16 | NA | NA | NULL | 0 | sCCA1.db |
| 67 | 6:28917608-6:29737971 | NULL | 6 | NA | NA | NULL | 0 | sCCA1.db |
| 68 | 6:28917608-6:29737971 | NULL | 6 | NA | NA | NULL | 0 | sCCA3.db |
| 69 | 7:128778386-7:130422414 | NULL | 7 | NA | NA | NULL | 0 | sCCA2.db |
| 70 | 6:39295677-6:40345115 | NULL | 6 | NA | NA | NULL | 0 | sCCA3.db |
| 71 | 16:8338519-16:9799419 | NULL | 16 | NA | NA | NULL | 0 | sCCA1.db |
| 72 | 10:10249396-10:12586797 | NULL | 10 | NA | NA | NULL | 0 | sCCA3.db |
| 73 | 21:32668642-21:34376999 | NULL | 21 | NA | NA | NULL | 0 | sCCA1.db |
| 74 | 7:7808752-7:9123423 | NULL | 7 | NA | NA | NULL | 0 | sCCA1.db |
| 75 | 9:33578334-9:34642243 | NULL | 9 | NA | NA | NULL | 0 | sCCA3.db |
| 76 | 6:132765669-6:134244243 | NULL | 6 | NA | NA | NULL | 0 | sCCA3.db |
| 77 | 10:10249396-10:12586797 | NULL | 10 | NA | NA | NULL | 0 | sCCA1.db |
| 78 | 21:32668642-21:34376999 | NULL | 21 | NA | NA | NULL | 0 | sCCA3.db |
| 79 | 6:132765669-6:134244243 | NULL | 6 | NA | NA | NULL | 0 | sCCA1.db |
| 80 | 13:102300523-13:104066710 | NULL | 13 | NA | NA | NULL | 0 | sCCA2.db |
| 81 | 7:128778386-7:130422414 | NULL | 7 | NA | NA | NULL | 0 | sCCA1.db |
| 82 | 10:10249396-10:12586797 | NULL | 10 | NA | NA | NULL | 0 | sCCA2.db |
| 83 | 10:119523934-10:120591353 | NULL | 10 | NA | NA | NULL | 0 | sCCA3.db |
| 84 | 4:120392684-4:122657987 | NULL | 4 | NA | NA | NULL | 0 | sCCA2.db |
| 85 | 13:102300523-13:104066710 | NULL | 13 | NA | NA | NULL | 0 | sCCA3.db |
| 86 | 15:67094767-15:69017999 | NULL | 15 | NA | NA | NULL | 0 | sCCA2.db |
| 87 | 10:119523934-10:120591353 | NULL | 10 | NA | NA | NULL | 0 | sCCA2.db |
| 88 | 13:54682864-13:55817131 | NULL | 13 | NA | NA | NULL | 0 | sCCA1.db |
| 89 | 4:87534648-4:89238028 | NULL | 4 | NA | NA | NULL | 0 | sCCA3.db |
| 90 | 6:39295677-6:40345115 | NULL | 6 | NA | NA | NULL | 0 | sCCA1.db |
| 91 | 21:32668642-21:34376999 | NULL | 21 | NA | NA | NULL | 0 | sCCA2.db |
| 92 | 4:87534648-4:89238028 | NULL | 4 | NA | NA | NULL | 0 | sCCA1.db |
| 93 | 4:87534648-4:89238028 | NULL | 4 | NA | NA | NULL | 0 | sCCA2.db |
| 94 | 6:39295677-6:40345115 | NULL | 6 | NA | NA | NULL | 0 | sCCA2.db |
| 95 | 15:67094767-15:69017999 | NULL | 15 | NA | NA | NULL | 0 | sCCA1.db |
| 96 | 13:102300523-13:104066710 | NULL | 13 | NA | NA | NULL | 0 | sCCA1.db |
| 97 | 15:67094767-15:69017999 | NULL | 15 | NA | NA | NULL | 0 | sCCA3.db |
| 98 | 10:119523934-10:120591353 | NULL | 10 | NA | NA | NULL | 0 | sCCA1.db |
| 99 | 6:160580497-6:162169564 | NULL | 6 | NA | NA | NULL | 0 | sCCA3.db |
| 100 | 17:47517400-17:49898957 | NULL | 17 | NA | NA | NULL | 0 | sCCA1.db |
| 101 | 7:7808752-7:9123423 | NULL | 7 | NA | NA | NULL | 0 | sCCA2.db |
| 102 | 9:4495328-9:4884926 | NULL | 9 | NA | NA | NULL | 0 | sCCA3.db |
| 103 | 12:32165592-12:33076989 | NULL | 12 | NA | NA | NULL | 0 | sCCA1.db |
| 104 | 9:4495328-9:4884926 | NULL | 9 | NA | NA | NULL | 0 | sCCA1.db |
| 105 | 12:32165592-12:33076989 | NULL | 12 | NA | NA | NULL | 0 | sCCA3.db |
| 106 | 7:7808752-7:9123423 | NULL | 7 | NA | NA | NULL | 0 | sCCA3.db |
| 107 | 6:29737971-6:30798168 | NULL | 6 | NA | NA | NULL | 0 | sCCA1.db |
| 108 | 6:160580497-6:162169564 | NULL | 6 | NA | NA | NULL | 0 | sCCA1.db |
| 109 | 9:4495328-9:4884926 | NULL | 9 | NA | NA | NULL | 0 | sCCA2.db |
| 110 | 6:29737971-6:30798168 | NULL | 6 | NA | NA | NULL | 0 | sCCA2.db |
| 111 | 6:29737971-6:30798168 | NULL | 6 | NA | NA | NULL | 0 | sCCA3.db |
| 112 | 4:120392684-4:122657987 | BBS7 | 4 | 121870496 | 121870497 | protein_coding | 3.26 | sCCA3.db |
| 113 | 6:160580497-6:162169564 | NULL | 6 | NA | NA | NULL | 0 | sCCA2.db |
| 114 | 12:32165592-12:33076989 | NULL | 12 | NA | NA | NULL | 0 | sCCA2.db |
| 115 | 6:30798168-6:31571218 | VARS1 | 6 | 31795952 | 31795953 | protein_coding | 2.94 | sCCA1.db |
| 116 | 6:32682664-6:33236497 | NULL | 6 | NA | NA | NULL | 0 | sCCA1.db |

**Supplementary Table 10.** Genes mapped by multi-marker analysis of genomic annotation

| **Number** | **Gene** | **CHR** | **Start** | **End** | **Z score** | **Type** | ***P* value** | ***P* bon** |
| --- | --- | --- | --- | --- | --- | --- | --- | --- |
| 1 | ATP6V1G2 | 6 | 31512239 | 31516204 | 6.1094 | protein_coding | 5e-10 | 8.527e-06 |
| 2 | NFKBIL1 | 6 | 31514647 | 31526606 | 6.1707 | protein_coding | 3.3986e-10 | 5.79597244e-06 |
| 3 | PRRT1 | 6 | 32116136 | 32122150 | 5.2527 | protein_coding | 7.4938e-08 | 0.001277992652 |
| 4 | PPT2 | 6 | 32121218 | 32134011 | 6.032 | protein_coding | 8.0953e-10 | 1.380572462e-05 |
| 5 | PPT2-EGFL8 | 6 | 32121622 | 32139755 | 5.7006 | protein_coding | 5.969e-09 | 0.000101795326 |
| 6 | EGFL8 | 6 | 32132360 | 32136058 | 6.1094 | protein_coding | 5e-10 | 8.527e-06 |
| 7 | AGPAT1 | 6 | 32135989 | 32145873 | 6.6477 | protein_coding | 1.4889e-11 | 2.53917006e-07 |
| 8 | RNF5 | 6 | 32146131 | 32151930 | 7.1595 | protein_coding | 4.0487e-13 | 6.90465298e-09 |
| 9 | PBX2 | 6 | 32152512 | 32157963 | 5.873 | protein_coding | 2.1397e-09 | 3.64904438e-05 |
| 10 | BTNL2 | 6 | 32361740 | 32374905 | 6.1938 | protein_coding | 2.9368e-10 | 5.00841872e-06 |
| 11 | HLA-DQA1 | 6 | 32595956 | 32614839 | 7.5129 | protein_coding | 2.8921e-14 | 4.93218734e-10 |
| 12 | HLA-DQB1 | 6 | 32627244 | 32636160 | 6.1094 | protein_coding | 5e-10 | 8.527e-06 |
| 13 | EME1 | 17 | 48450581 | 48458844 | 4.9502 | protein_coding | 3.7064e-07 | 0.00632089456 |

Abbreviations: *P* bon = *P* value after Bonferroni multiple test correction.

**Supplementary Table 11.** Enriched Pathways by the Molecular Signatures Database (MSigDB)

| **Number** | **Category** | **Gene Set** | **Number of genes** | **Number of overlap** | ***P* value** | ***P* FDR** |
| --- | --- | --- | --- | --- | --- | --- |
| 1 | GWAS catalog | Ulcerative colitis | 357 | 8 | 2.52992376909659e-12 | 1.11949126782524e-08 |
| 2 | GWAS catalog | Autism spectrum disorder or schizophrenia | 469 | 8 | 2.25071050901111e-11 | 4.97969700118707e-08 |
| 3 | GWAS catalog | Inflammatory bowel disease | 605 | 8 | 1.71490406550066e-10 | 2.52948349661348e-07 |
| 4 | GWAS catalog | Prostate cancer | 297 | 6 | 6.53316510813732e-09 | 7.22731390087691e-06 |
| 5 | GWAS catalog | Idiopathic membranous nephropathy | 8 | 3 | 8.42526535817299e-09 | 7.45635984198309e-06 |
| 6 | GWAS catalog | Asthma | 772 | 7 | 5.61718911802329e-08 | 4.14267697454218e-05 |
| 7 | GWAS catalog | Sarcoidosis (non-Lofgren's syndrome without extrapulmonary manifestations) | 23 | 3 | 2.65171032428254e-07 | 0.000167625974070718 |
| 8 | GWAS catalog | Oleic acid (18:1n-9) levels | 11 | 2 | 1.71969159942361e-05 | 0.00951204415931185 |
| 9 | GWAS catalog | Clozapine-induced agranulocytosis/granulocytopenia in treatment-resistant schizophrenia | 16 | 2 | 3.74604791273524e-05 | 0.0184180689042816 |
| 10 | GWAS catalog | Type 1 diabetes and autoimmune thyroid diseases | 17 | 2 | 4.24416080631012e-05 | 0.0187804115679223 |
| 11 | Positional gene sets | chr6p21 | 281 | 8 | 3.68825274250172e-13 | 1.10647582275051e-10 |

Abbreviations: *P* FDR = *P* value after [False Discovery Rate](https://blog.csdn.net/weixin_61365716/article/details/137372660) test correction.

**Supplementary Table 12.** Heritability Enrichment Across Genomic Functional and Regulatory Regions

| **Number** | **Category** | **Enrichment** | **Coefficient** | **SE** | **Z score** | ***P* FDR** |
| --- | --- | --- | --- | --- | --- | --- |
| 1 | baseL2_0 | 1 | -2.11718596806687e-10 | 1.20999166954763e-10 | -1.74975251594781 | NA |
| 2 | Coding_UCSC.bedL2_0 | 10.1450789195667 | 6.2418846228517e-10 | 3.11599981758358e-10 | 2.00317233256201 | 0.00615851834091062 |
| 3 | Coding_UCSC.extend.500.bedL2_0 | 1.68192514070605 | -1.2225596783848e-10 | 8.10661169224128e-11 | -1.50810193555328 | 0.714955507807309 |
| 4 | Conserved_LindbladToh.bedL2_0 | 19.7212232685101 | 2.0681852257751e-09 | 5.33203140462138e-10 | 3.87879415710599 | 0.000281066787930074 |
| 5 | Conserved_LindbladToh.extend.500.bedL2_0 | 0.972036576795269 | -1.87800706444272e-10 | 1.63762614250571e-10 | -1.14678620211156 | 0.972320021238609 |
| 6 | CTCF_Hoffman.bedL2_0 | -4.70785699590576 | -6.27630960707647e-10 | 3.83746756915102e-10 | -1.63553424074018 | 0.350213864004163 |
| 7 | CTCF_Hoffman.extend.500.bedL2_0 | 0.507662623786462 | 9.84379082460104e-11 | 1.35297752350669e-10 | 0.727564993030155 | 0.741427178131691 |
| 8 | DGF_ENCODE.bedL2_0 | -1.24636571256908 | -2.65187171571638e-10 | 2.21592602445249e-10 | -1.19673296240636 | 0.564832456498134 |
| 9 | DGF_ENCODE.extend.500.bedL2_0 | 1.30614186484698 | -1.88868716525599e-11 | 6.83160521491873e-11 | -0.276463159951267 | 0.564832456498134 |
| 10 | DHS_peaks_Trynka.bedL2_0 | -0.599225485033759 | 4.62244586052795e-10 | 2.05030300623085e-10 | 2.25451840361175 | 0.741427178131691 |
| 11 | DHS_Trynka.bedL2_0 | -2.27916884202828 | -1.00097837054239e-09 | 4.76101661422074e-10 | -2.10244670760558 | 0.564832456498134 |
| 12 | DHS_Trynka.extend.500.bedL2_0 | 1.94999804385504 | 4.09231271355067e-10 | 2.60643746224785e-10 | 1.57007899588021 | 0.350213864004163 |
| 13 | Enhancer_Andersson.bedL2_0 | 0.774970725556905 | 7.28959332596338e-10 | 6.19409315129508e-10 | 1.17686207616029 | 0.972320021238609 |
| 14 | Enhancer_Andersson.extend.500.bedL2_0 | -3.62064864750222 | -7.58939934475311e-10 | 5.19008242856409e-10 | -1.46228878812871 | 0.564832456498134 |
| 15 | Enhancer_Hoffman.bedL2_0 | 3.78971847839903 | 4.66764173449277e-10 | 2.20459556413407e-10 | 2.1172326618221 | 0.0571436386154032 |
| 16 | Enhancer_Hoffman.extend.500.bedL2_0 | 1.99624270646282 | -1.73026463830855e-10 | 1.79303549833529e-10 | -0.96499184757635 | 0.496372332965999 |
| 17 | FetalDHS_Trynka.bedL2_0 | -1.53159235086582 | -4.0796089371229e-11 | 3.05697946904737e-10 | -0.133452284466739 | 0.710243279618505 |
| 18 | FetalDHS_Trynka.extend.500.bedL2_0 | 1.25273565651966 | -1.20009637927866e-10 | 9.74363521405411e-11 | -1.23167211509279 | 0.597442019961668 |
| 19 | H3K27ac_Hnisz.bedL2_0 | 1.18266377847814 | -3.07044956459687e-10 | 2.20319286590615e-10 | -1.39363630488792 | 0.593594521510919 |
| 20 | H3K27ac_Hnisz.extend.500.bedL2_0 | 1.44179859336881 | 2.70880931580704e-10 | 2.12018322185666e-10 | 1.27762982363143 | 0.0709443596473869 |
| 21 | H3K27ac_PGC2.bedL2_0 | 2.57097493005769 | 5.6041635929792e-10 | 7.21820113716865e-10 | 0.776393382018923 | 0.564832456498134 |
| 22 | H3K27ac_PGC2.extend.500.bedL2_0 | 1.54267491904202 | -3.75205224358757e-10 | 5.20246453015739e-10 | -0.721206693834791 | 0.0693275016417602 |
| 23 | H3K4me1_peaks_Trynka.bedL2_0 | 3.00463326819202 | 3.48474770990034e-10 | 3.33754288120961e-10 | 1.04410574902857 | 0.549509574910964 |
| 24 | H3K4me1_Trynka.bedL2_0 | 1.22155158535302 | -2.40052818109985e-10 | 1.41001749147674e-10 | -1.70248113630543 | 0.791346339049735 |
| 25 | H3K4me1_Trynka.extend.500.bedL2_0 | 1.5805737859667 | 1.7415123653902e-10 | 1.45902864073049e-10 | 1.19361081528755 | 0.0481619079964625 |
| 26 | H3K4me3_peaks_Trynka.bedL2_0 | 2.2208998670465 | 5.18333217018079e-11 | 2.34481691995598e-10 | 0.221054877507371 | 0.645965865878316 |
| 27 | H3K4me3_Trynka.bedL2_0 | 2.03936512816161 | 5.18234692008365e-11 | 1.24979631678066e-10 | 0.414655320271133 | 0.0709443596473869 |
| 28 | H3K4me3_Trynka.extend.500.bedL2_0 | 1.62673129168251 | -3.85854252102565e-11 | 7.39841551078063e-11 | -0.521536336449765 | 0.210864778737346 |
| 29 | H3K9ac_peaks_Trynka.bedL2_0 | 2.79126003356934 | 3.10014048756193e-11 | 6.56801099212234e-10 | 0.047200598343703 | 0.795741381678056 |
| 30 | H3K9ac_Trynka.bedL2_0 | 2.71239663756521 | 1.62216381692275e-10 | 1.86308130917039e-10 | 0.87068868596244 | 0.0333984290468712 |
| 31 | H3K9ac_Trynka.extend.500.bedL2_0 | 1.98782856648596 | 9.09217911444403e-12 | 8.38530099226908e-11 | 0.108429967186946 | 0.0693275016417602 |
| 32 | Intron_UCSC.bedL2_0 | 1.11532415661793 | -3.80345286554051e-10 | 4.83559940828217e-10 | -0.786552512812816 | 0.597442019961668 |
| 33 | Intron_UCSC.extend.500.bedL2_0 | 1.37412616087755 | 4.09402493634722e-10 | 4.86532404755533e-10 | 0.841470145941119 | 0.0693275016417602 |
| 34 | PromoterFlanking_Hoffman.bedL2_0 | 2.89549229236445 | 2.43755785940376e-10 | 5.26207360508492e-10 | 0.463231425924614 | 0.791346339049735 |
| 35 | PromoterFlanking_Hoffman.extend.500.bedL2_0 | 0.509845584820857 | -2.8160614105176e-10 | 2.46680934098447e-10 | -1.14158048768931 | 0.795741381678056 |
| 36 | Promoter_UCSC.bedL2_0 | 1.40298523398297 | 2.75299919700056e-10 | 7.93690770295981e-10 | 0.34686042726362 | 0.795741381678056 |
| 37 | Promoter_UCSC.extend.500.bedL2_0 | 0.822002624964345 | -3.32580994339999e-10 | 6.70892117562914e-10 | -0.495729470705565 | 0.903339583657506 |
| 38 | Repressed_Hoffman.bedL2_0 | 0.514240870325604 | -5.91734502505616e-12 | 1.35717120341958e-10 | -0.0436005789847781 | 0.710243279618505 |
| 39 | Repressed_Hoffman.extend.500.bedL2_0 | 0.938120192103378 | 1.84996615583556e-10 | 2.06896123732511e-10 | 0.894152158320433 | 0.710243279618505 |
| 40 | SuperEnhancer_Hnisz.bedL2_0 | 1.24835041499048 | -1.8856511792284e-09 | 2.03029758252831e-09 | -0.92875605795689 | 0.187324481324903 |
| 41 | SuperEnhancer_Hnisz.extend.500.bedL2_0 | 1.6031679578067 | 1.85332260855272e-09 | 1.99341555389273e-09 | 0.929722156995095 | 0.270920237996322 |
| 42 | TFBS_ENCODE.bedL2_0 | 0.438842283743514 | 1.89014733113819e-11 | 1.651924462054e-10 | 0.114420929924845 | 0.795741381678056 |
| 43 | TFBS_ENCODE.extend.500.bedL2_0 | 1.67133300814741 | 6.50837627703962e-11 | 1.09990520415974e-10 | 0.591721564042568 | 0.352136534954172 |
| 44 | Transcribed_Hoffman.bedL2_0 | 2.03805790707195 | 2.03053411957531e-10 | 1.68989222029614e-10 | 1.20157610952222 | 0.593594521510919 |
| 45 | Transcribed_Hoffman.extend.500.bedL2_0 | 1.1225437741611 | -2.32576753672671e-11 | 1.05889397832958e-10 | -0.219641209065673 | 0.564832456498134 |
| 46 | TSS_Hoffman.bedL2_0 | -0.276522667517952 | -3.77341013044839e-10 | 4.81982643234591e-10 | -0.782893364193572 | 0.834278134121505 |
| 47 | TSS_Hoffman.extend.500.bedL2_0 | 2.28033908849927 | 3.12861887345699e-10 | 2.41555859472822e-10 | 1.29519477618343 | 0.638681792987161 |
| 48 | UTR_3_UCSC.bedL2_0 | 2.25167909294724 | -3.89902361804645e-10 | 4.795224258271e-10 | -0.813105583398159 | 0.791346339049735 |
| 49 | UTR_3_UCSC.extend.500.bedL2_0 | 1.36295213126499 | -4.24699640030446e-11 | 1.81720607968089e-10 | -0.233710224051762 | 0.831872436331421 |
| 50 | UTR_5_UCSC.bedL2_0 | 4.75731475373395 | -1.47418544084098e-10 | 3.74023264449942e-10 | -0.394142712755847 | 0.564832456498134 |
| 51 | UTR_5_UCSC.extend.500.bedL2_0 | 1.69001575275441 | 4.55607744605199e-11 | 1.37882198748618e-10 | 0.330432607501312 | 0.791346339049735 |
| 52 | WeakEnhancer_Hoffman.bedL2_0 | 3.88900485391725 | 2.59581579904798e-10 | 2.41071055839172e-10 | 1.07678451484435 | 0.337857284301324 |
| 53 | WeakEnhancer_Hoffman.extend.500.bedL2_0 | 2.99794038915946 | 1.58677083386277e-10 | 1.01041123001605e-10 | 1.57042082146847 | 0.0693275016417602 |

Abbreviations: *P* FDR = *P* value after [False Discovery Rate](https://blog.csdn.net/weixin_61365716/article/details/137372660) test correction.

**Supplementary Table 13.** Polygenic Risk Score and Genetic Contribution Across Chromosomal Regions

| **CHR** | **PRS Score Sum** |
| --- | --- |
| 1 | 0.0151090813696779 |
| 2 | -0.0218353203794986 |
| 3 | 0.0387590982231304 |
| 4 | -0.010068882106643 |
| 5 | -0.012407806075944 |
| 6 | -0.00302536087282091 |
| 7 | 0.00825858014339812 |
| 8 | -0.000384683735466353 |
| 9 | -0.010648367641591 |
| 10 | -0.00504901154611895 |
| 11 | -0.0103666450379779 |
| 12 | -0.0091593558568441 |
| 13 | -0.028751881981009 |
| 14 | -0.00432175988479003 |
| 15 | -0.00462212979227203 |
| 16 | 0.00333959671246594 |
| 17 | 0.001783134488078 |
| 18 | -0.00905056969738403 |
| 19 | 0.00506911286311699 |
| 20 | -0.017275516895837 |
| 21 | -0.004167925641845 |
| 22 | 0.000695477446789996 |
