# Supplementary material for: Integrating genomic structural equation modeling and experimental validation to unravel the genetic basis of male genital lichen sclerosus
Source: Front Immunol. 2026 Jul 16;17:1857945. doi: 10.3389/fimmu.2026.1857945 (PMC13435250; doi:10.3389/fimmu.2026.1857945)
Supplement: Supplementary file 3 [file SupplementaryFile2.zip › Sfig_rs16948981_locusZoom.pdf]

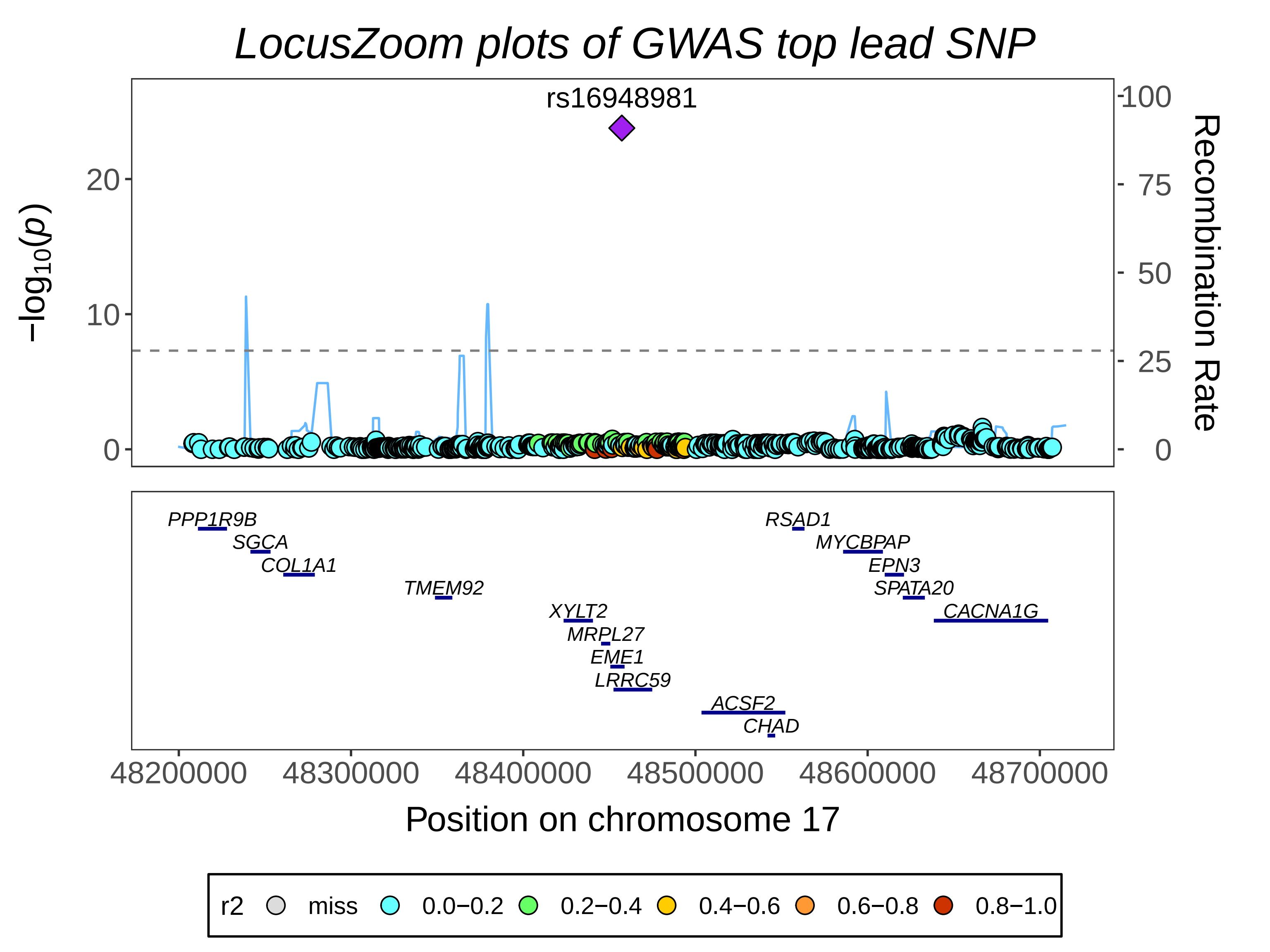

LocusZoom plots of GWAS top lead SNP
rs16948981
−log10(p)
Recombination Rate
20
10
0
100
75
50
25
0
PPP1R9B
SGCA
COL1A1
TMEM92
XYLT2
MRPL27
EME1
LRRC59
ACSF2
CHAD
RSAD1
MYCBPAP
EPN3
SPATA20
CACNA1G
48200000
48300000
48400000
48500000
48600000
48700000
Position on chromosome 17
r2
miss
0.0−0.2
0.2−0.4
0.4−0.6
0.6−0.8
0.8−1.0
