# Supplementary material for: Integrating genomic structural equation modeling and experimental validation to unravel the genetic basis of male genital lichen sclerosus
Source: Front Immunol. 2026 Jul 16;17:1857945. doi: 10.3389/fimmu.2026.1857945 (PMC13435250; doi:10.3389/fimmu.2026.1857945)
Supplement: Supplementary file 3 [file SupplementaryFile2.zip › Sfig_rs12924770_locusZoom.pdf]

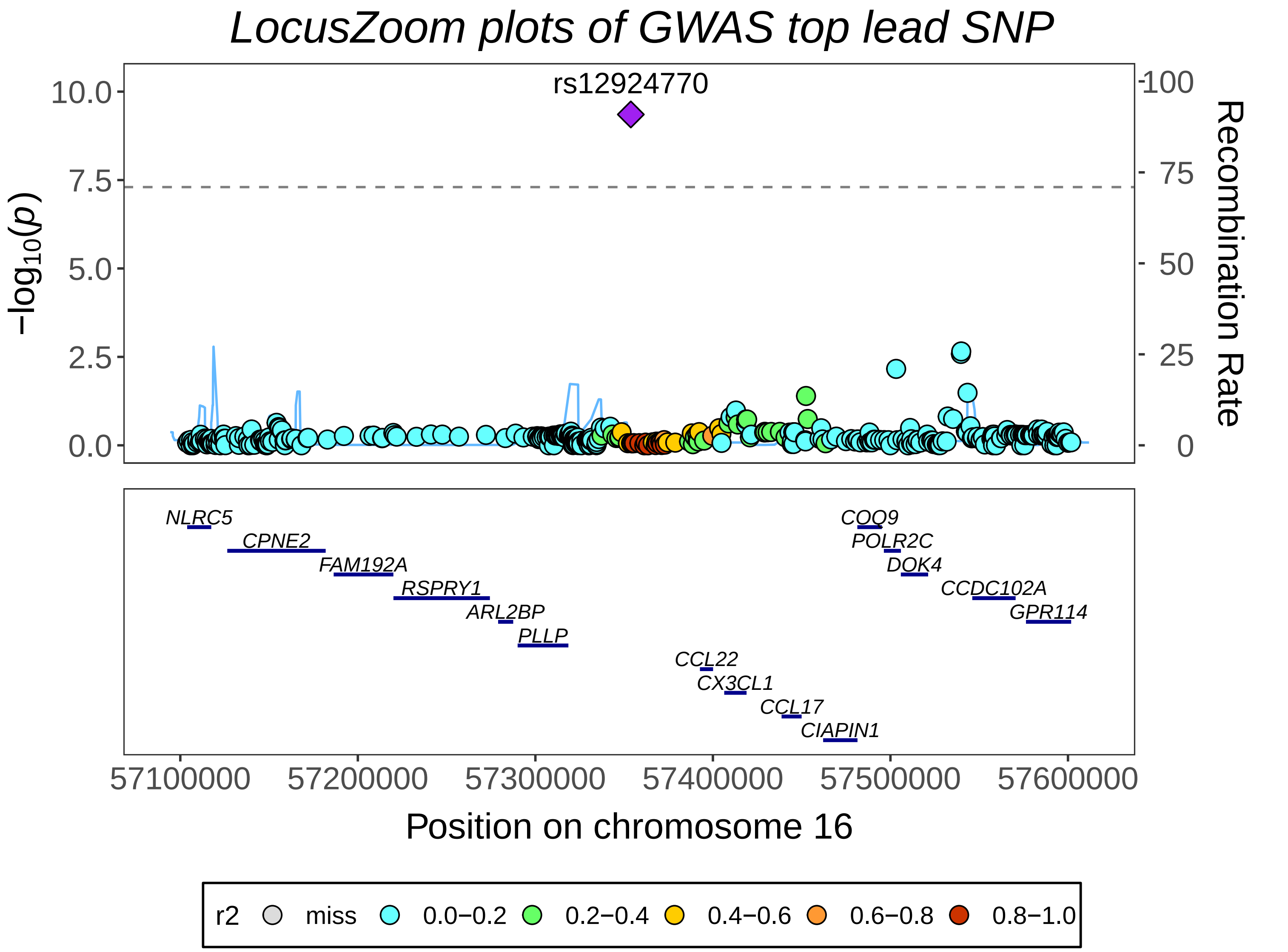

LocusZoom plots of GWAS top lead SNP
rs12924770
−log10(p)
10.0
7.5
5.0
2.5
0.0
Recombination Rate
100
75
50
25
0
NLRC5
CPNE2
FAM192A
RSPRY1
ARL2BP
PLLP
CCL22
CX3CL1
CCL17
CIAPIN1
COQ9
POLR2C
DOK4
CCDC102A
GPR114
57100000
57200000
57300000
57400000
57500000
57600000
Position on chromosome 16
r2
miss
0.0−0.2
0.2−0.4
0.4−0.6
0.6−0.8
0.8−1.0
