# Supplementary material for: Integrating genomic structural equation modeling and experimental validation to unravel the genetic basis of male genital lichen sclerosus
Source: Front Immunol. 2026 Jul 16;17:1857945. doi: 10.3389/fimmu.2026.1857945 (PMC13435250; doi:10.3389/fimmu.2026.1857945)
Supplement: Supplementary file 3 [file SupplementaryFile2.zip › Sfig_rs1428806_locusZoom.pdf]

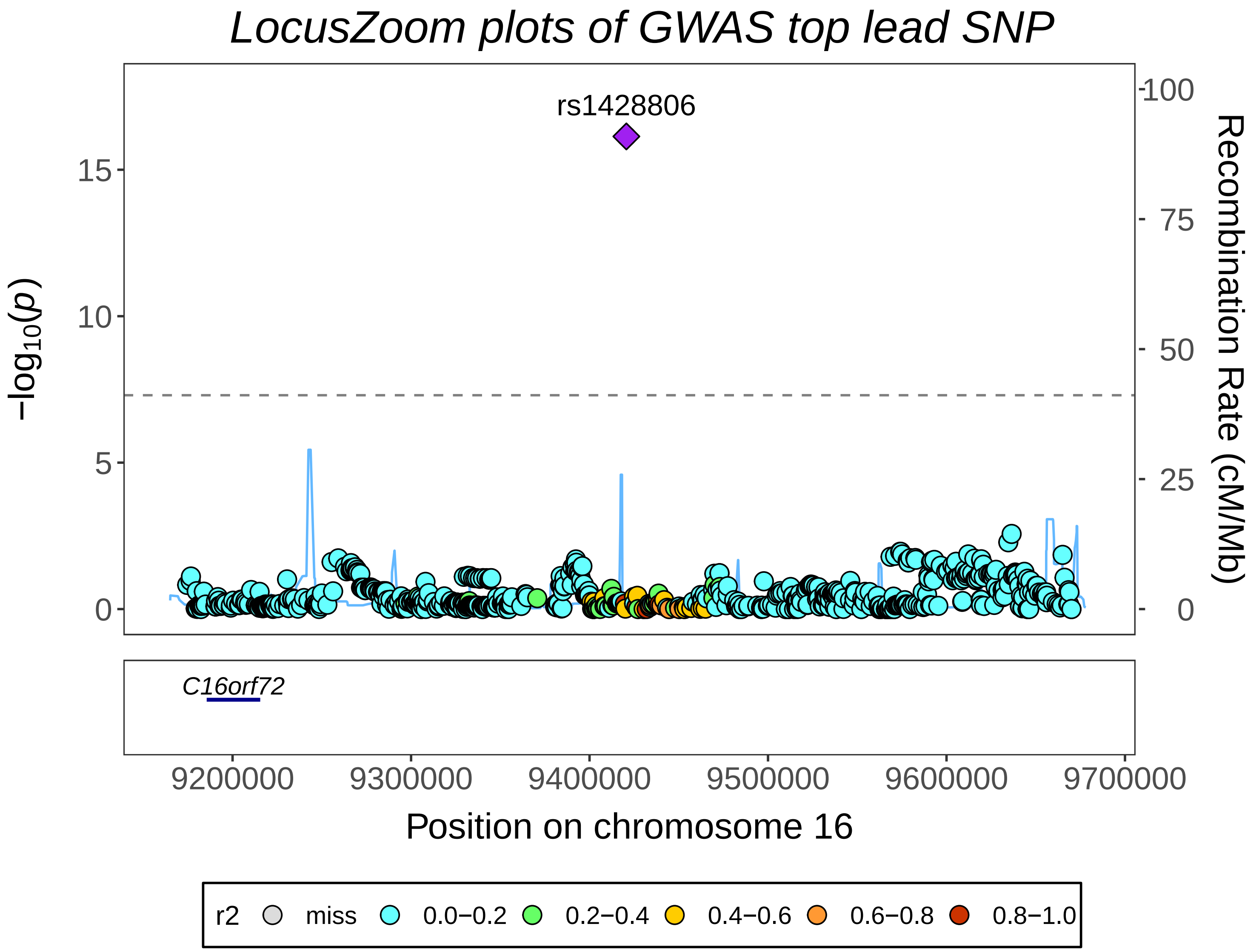

LocusZoom plots of GWAS top lead SNP
rs1428806
−log10(p)
0
5
10
15
Recombination Rate (cM/Mb)
0
25
50
75
100
C16orf72
9200000
9300000
9400000
9500000
9600000
9700000
Position on chromosome 16
r2
miss
0.0−0.2
0.2−0.4
0.4−0.6
0.6−0.8
0.8−1.0
