# Supplementary material for: Integrating genomic structural equation modeling and experimental validation to unravel the genetic basis of male genital lichen sclerosus
Source: Front Immunol. 2026 Jul 16;17:1857945. doi: 10.3389/fimmu.2026.1857945 (PMC13435250; doi:10.3389/fimmu.2026.1857945)
Supplement: Supplementary file 3 [file SupplementaryFile2.zip › Sfig_rs7153028_locusZoom.pdf]

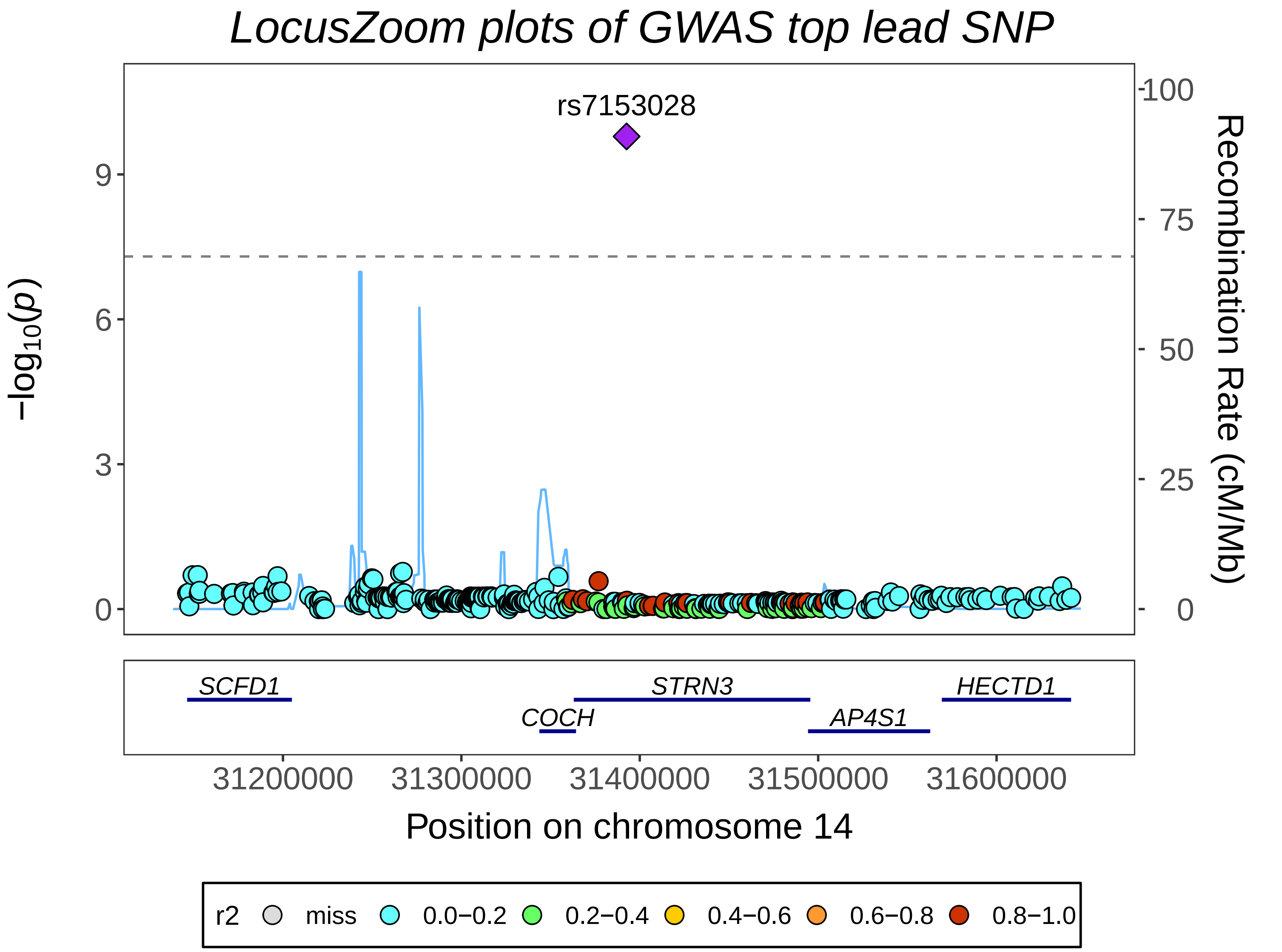
LocusZoom plots of GWAS top lead SNP
rs7153028
−log10(p)
0
3
6
9
Recombination Rate (cM/Mb)
0
25
50
75
100
SCFD1
STRN3
HECTD1
COCH
AP4S1
31200000
31300000
31400000
31500000
31600000
Position on chromosome 14
r2
miss
0.0−0.2
0.2−0.4
0.4−0.6
0.6−0.8
0.8−1.0
