# Supplementary material for: Integrating genomic structural equation modeling and experimental validation to unravel the genetic basis of male genital lichen sclerosus
Source: Front Immunol. 2026 Jul 16;17:1857945. doi: 10.3389/fimmu.2026.1857945 (PMC13435250; doi:10.3389/fimmu.2026.1857945)
Supplement: Supplementary file 3 [file SupplementaryFile2.zip › Sfig_rs2554379_locusZoom.pdf]

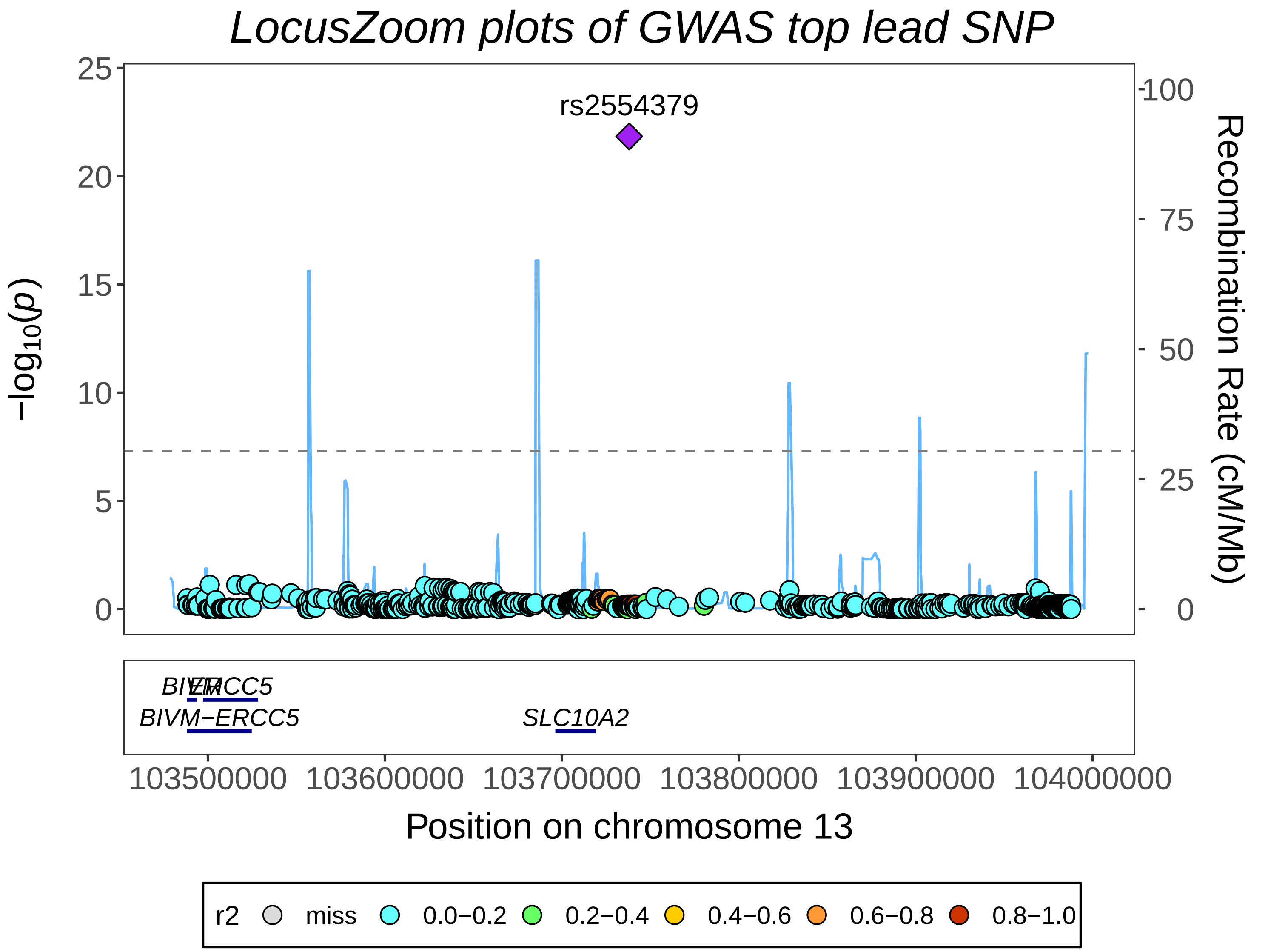
LocusZoom plots of GWAS top lead SNP
rs2554379
−log10(p)
Recombination Rate (cM/Mb)
0
5
10
15
20
25
0
25
50
75
100
BIVM
ERCC5
BIVM−ERCC5
SLC10A2
103500000
103600000
103700000
103800000
103900000
104000000
Position on chromosome 13
r2
miss
0.0−0.2
0.2−0.4
0.4−0.6
0.6−0.8
0.8−1.0
