# Supplementary material for: Integrating genomic structural equation modeling and experimental validation to unravel the genetic basis of male genital lichen sclerosus
Source: Front Immunol. 2026 Jul 16;17:1857945. doi: 10.3389/fimmu.2026.1857945 (PMC13435250; doi:10.3389/fimmu.2026.1857945)
Supplement: Supplementary file 3 [file SupplementaryFile2.zip › Sfig_rs12830815_locusZoom.pdf]

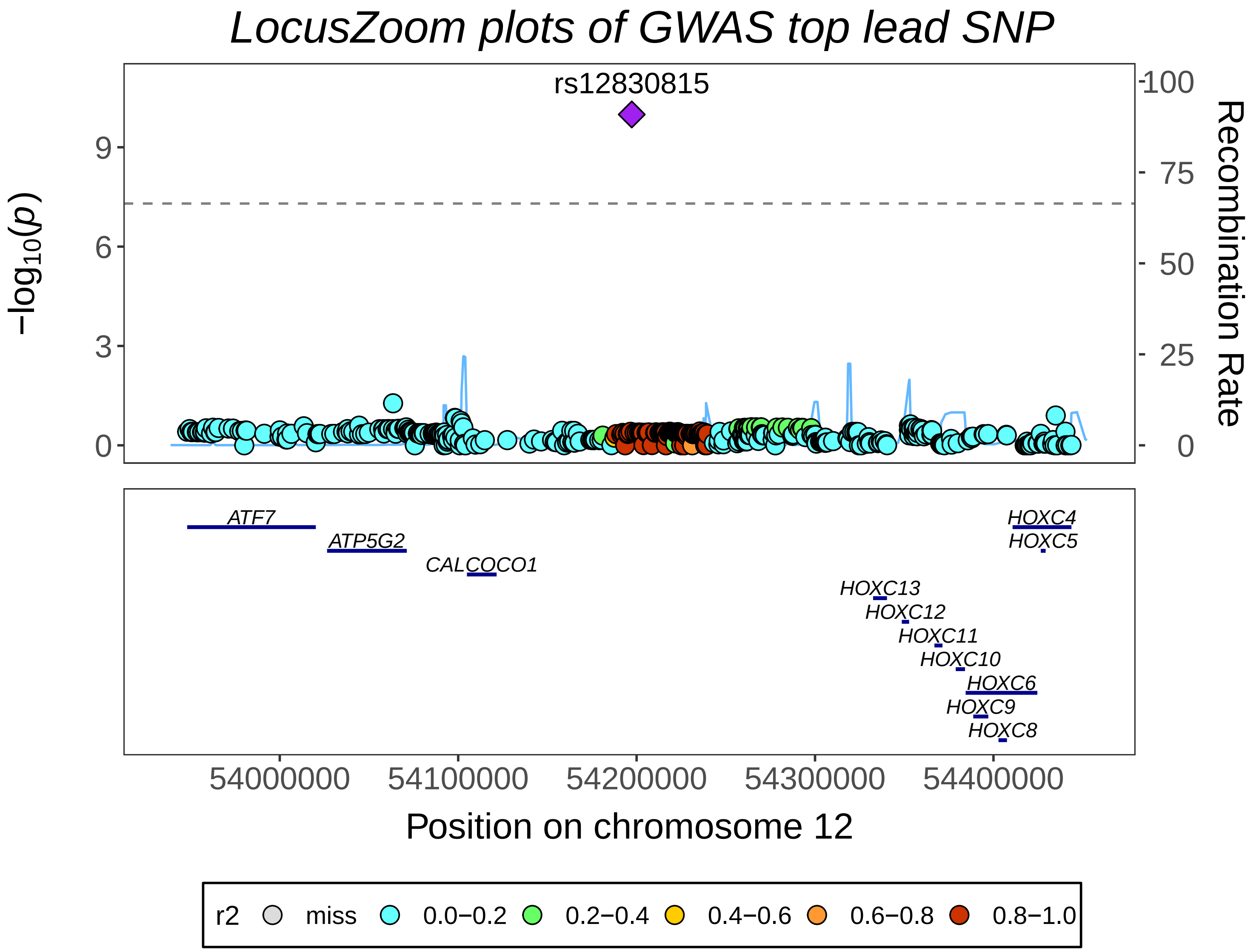

LocusZoom plots of GWAS top lead SNP
rs12830815
−log10(p)
Recombination Rate
0
3
6
9
0
25
50
75
100
ATF7
ATP5G2
CALCOCO1
HOXC13
HOXC12
HOXC11
HOXC10
HOXC6
HOXC9
HOXC8
HOXC4
HOXC5
54000000
54100000
54200000
54300000
54400000
Position on chromosome 12
r2
miss
0.0−0.2
0.2−0.4
0.4−0.6
0.6−0.8
0.8−1.0
