# Supplementary material for: Integrating genomic structural equation modeling and experimental validation to unravel the genetic basis of male genital lichen sclerosus
Source: Front Immunol. 2026 Jul 16;17:1857945. doi: 10.3389/fimmu.2026.1857945 (PMC13435250; doi:10.3389/fimmu.2026.1857945)
Supplement: Supplementary file 3 [file SupplementaryFile2.zip › Sfig_rs10844385_locusZoom.pdf]

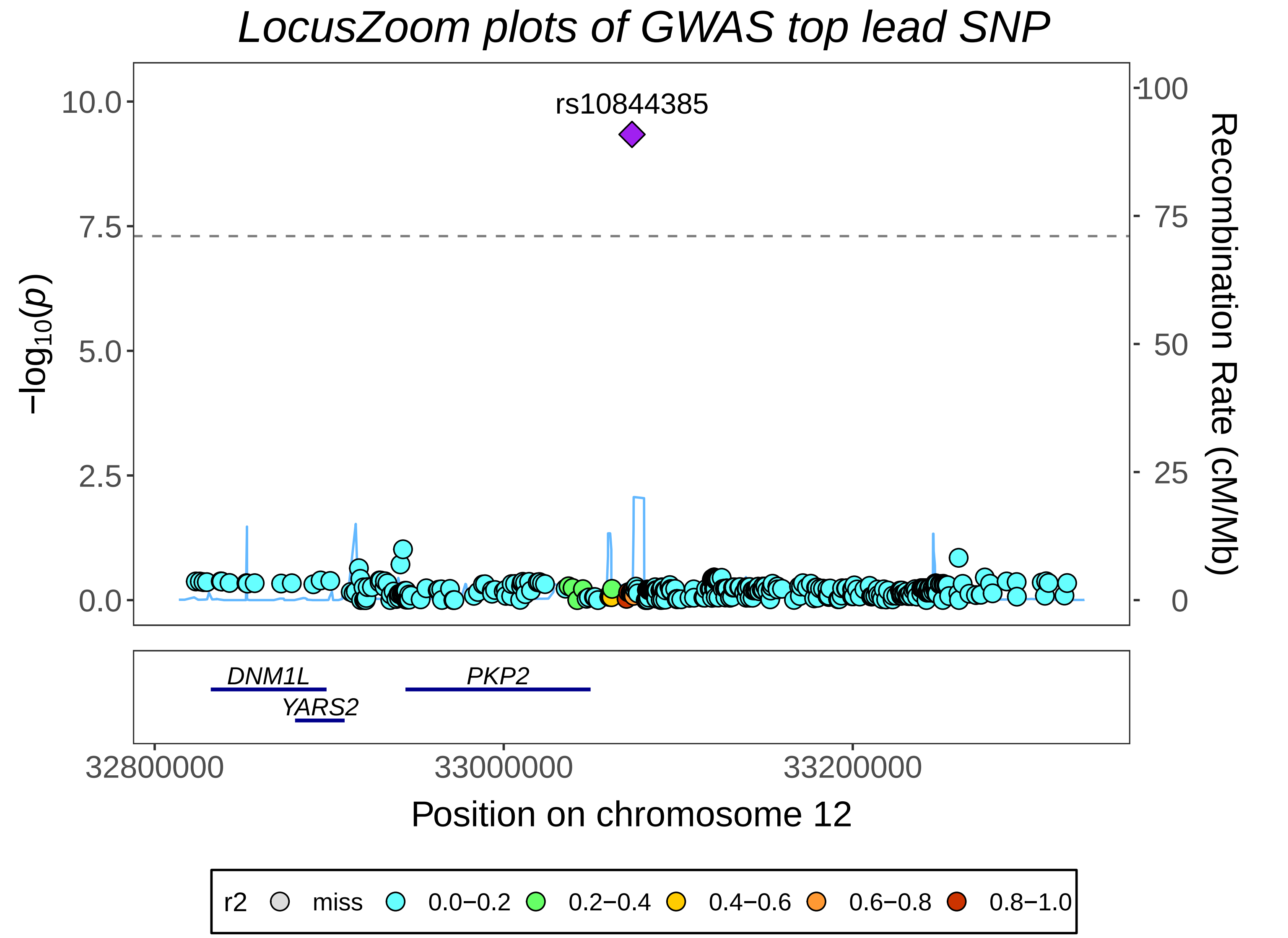
LocusZoom plots of GWAS top lead SNP
rs10844385
10.0
7.5
5.0
2.5
0.0
-log10(p)
100
75
50
25
0
Recombination Rate (cM/Mb)
DNM1L
YARS2
PKP2
32800000
33000000
33200000
Position on chromosome 12
r2
miss
0.0−0.2
0.2−0.4
0.4−0.6
0.6−0.8
0.8−1.0
