# Supplementary material for: Integrating genomic structural equation modeling and experimental validation to unravel the genetic basis of male genital lichen sclerosus
Source: Front Immunol. 2026 Jul 16;17:1857945. doi: 10.3389/fimmu.2026.1857945 (PMC13435250; doi:10.3389/fimmu.2026.1857945)
Supplement: Supplementary file 3 [file SupplementaryFile2.zip › Sfig_rs74353793_locusZoom.pdf]

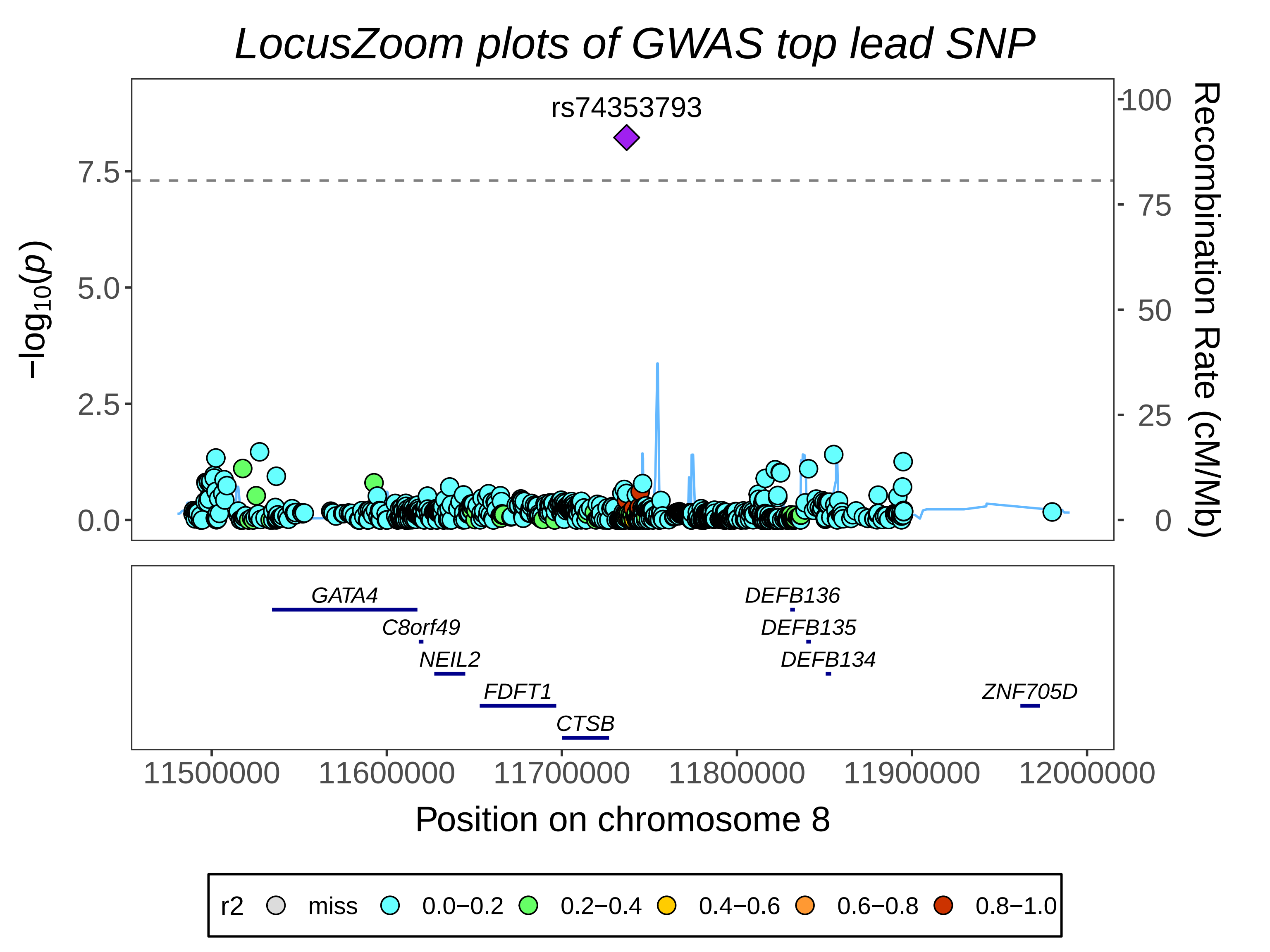
LocusZoom plots of GWAS top lead SNP
rs74353793
−log10(p)
7.5
5.0
2.5
0.0
Recombination Rate (cM/Mb)
100
75
50
25
0
GATA4
C8orf49
NEIL2
FDFT1
CTSB
DEFB136
DEFB135
DEFB134
ZNF705D
11500000
11600000
11700000
11800000
11900000
12000000
Position on chromosome 8
r2
miss
0.0−0.2
0.2−0.4
0.4−0.6
0.6−0.8
0.8−1.0
