# Supplementary material for: Integrating genomic structural equation modeling and experimental validation to unravel the genetic basis of male genital lichen sclerosus
Source: Front Immunol. 2026 Jul 16;17:1857945. doi: 10.3389/fimmu.2026.1857945 (PMC13435250; doi:10.3389/fimmu.2026.1857945)
Supplement: Supplementary file 3 [file SupplementaryFile2.zip › Sfig_rs12669390_locusZoom.pdf]

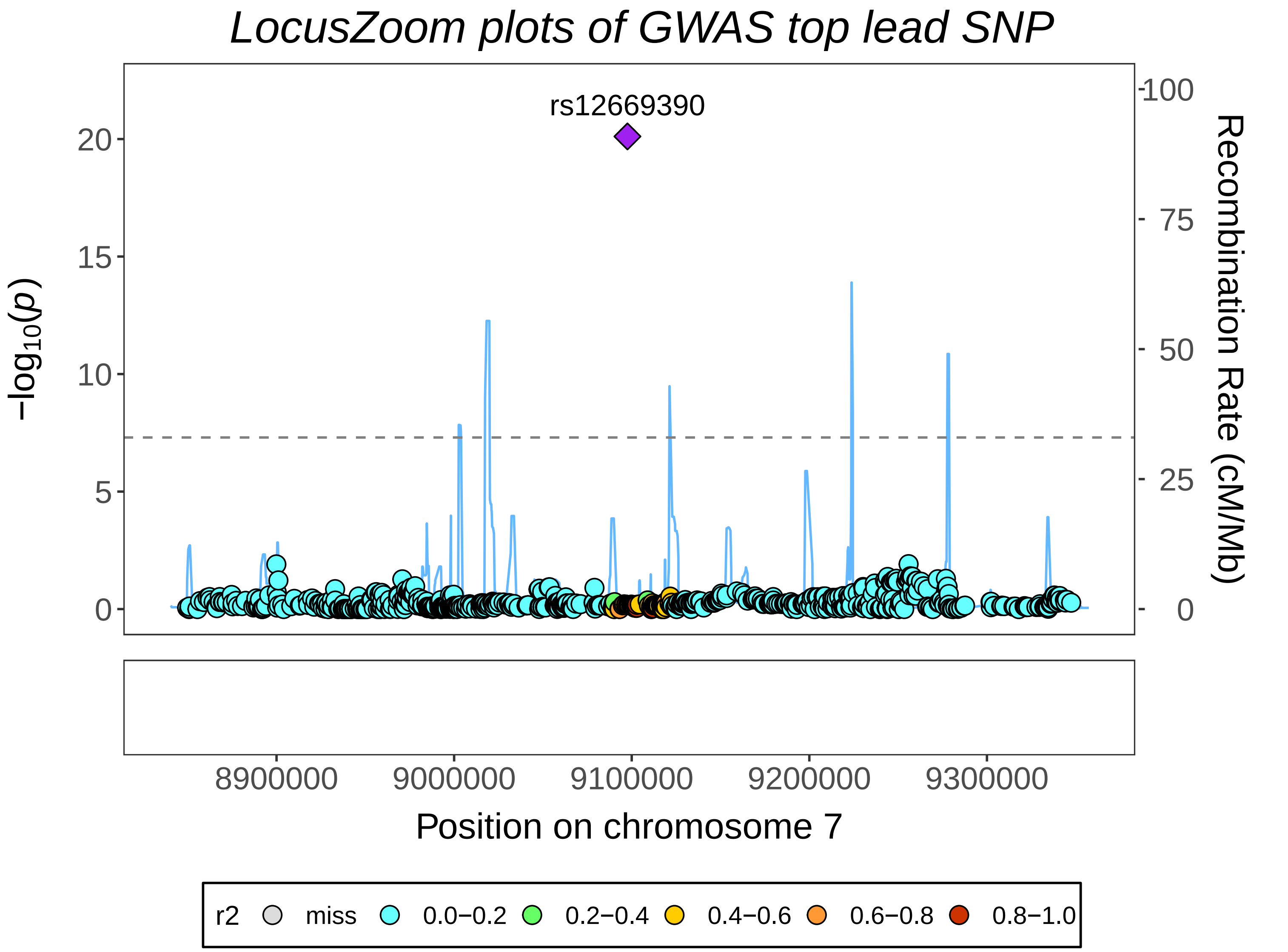

LocusZoom plots of GWAS top lead SNP
rs12669390
20
15
10
5
0
−log10(p)
100
75
50
25
0
Recombination Rate (cM/Mb)
8900000
9000000
9100000
9200000
9300000
Position on chromosome 7
r2
miss
0.0−0.2
0.2−0.4
0.4−0.6
0.6−0.8
0.8−1.0
