# Supplementary material for: Integrating genomic structural equation modeling and experimental validation to unravel the genetic basis of male genital lichen sclerosus
Source: Front Immunol. 2026 Jul 16;17:1857945. doi: 10.3389/fimmu.2026.1857945 (PMC13435250; doi:10.3389/fimmu.2026.1857945)
Supplement: Supplementary file 3 [file SupplementaryFile2.zip › Sfig_rs17843583_locusZoom.pdf]

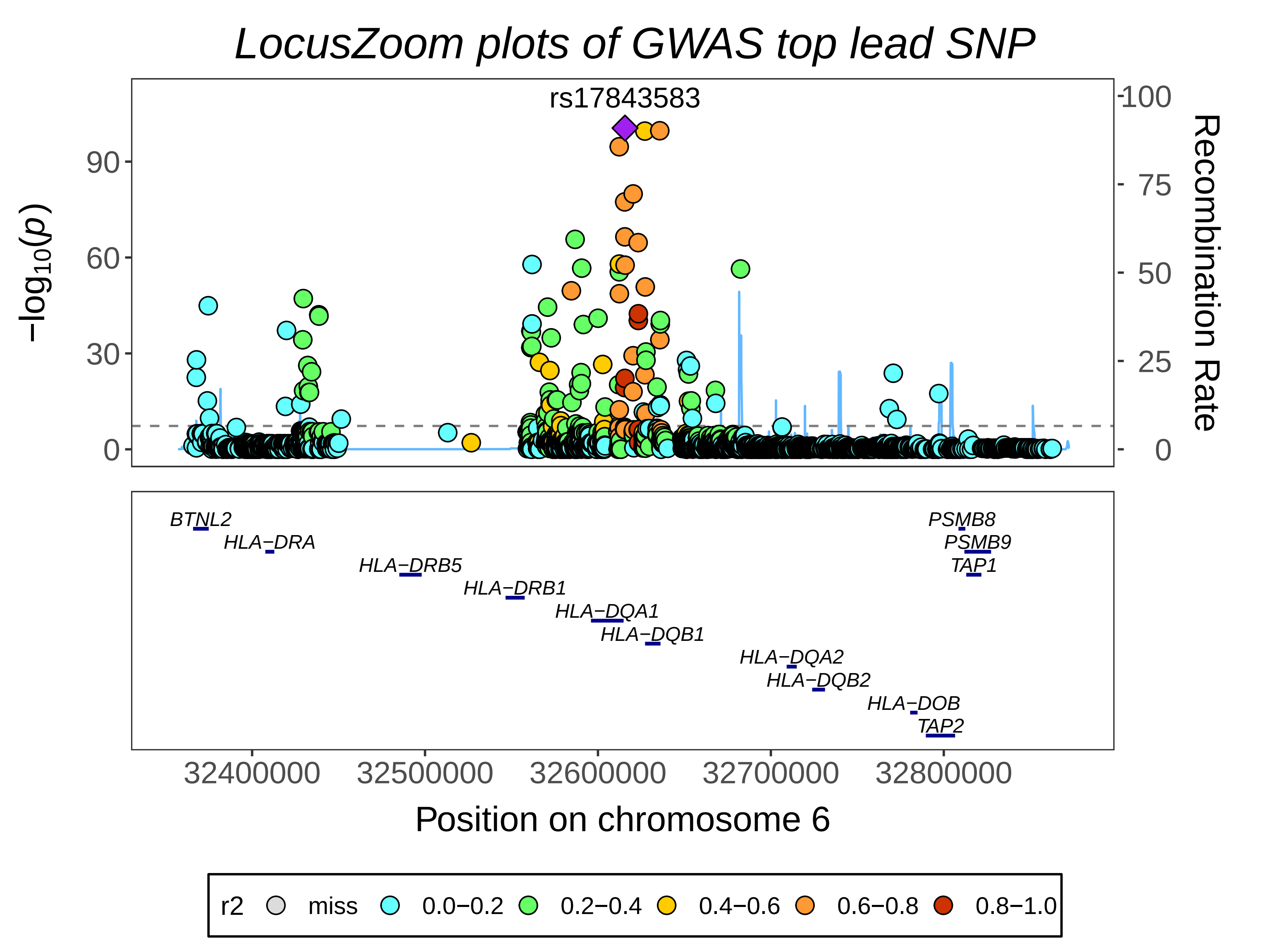
LocusZoom plots of GWAS top lead SNP
rs17843583
$-\log_{10}(p)$
0
30
60
90
Recombination Rate
0
25
50
75
100
BTNL2
HLA−DRA
HLA−DRB5
HLA−DRB1
HLA−DQA1
HLA−DQB1
HLA−DQA2
HLA−DQB2
HLA−DOB
TAP2
PSMB8
PSMB9
TAP1
32400000
32500000
32600000
32700000
32800000
Position on chromosome 6
r2
miss
0.0−0.2
0.2−0.4
0.4−0.6
0.6−0.8
0.8−1.0
