# Supplementary material for: Integrating genomic structural equation modeling and experimental validation to unravel the genetic basis of male genital lichen sclerosus
Source: Front Immunol. 2026 Jul 16;17:1857945. doi: 10.3389/fimmu.2026.1857945 (PMC13435250; doi:10.3389/fimmu.2026.1857945)
Supplement: Supplementary file 3 [file SupplementaryFile2.zip › Sfig_rs3131084_locusZoom.pdf]

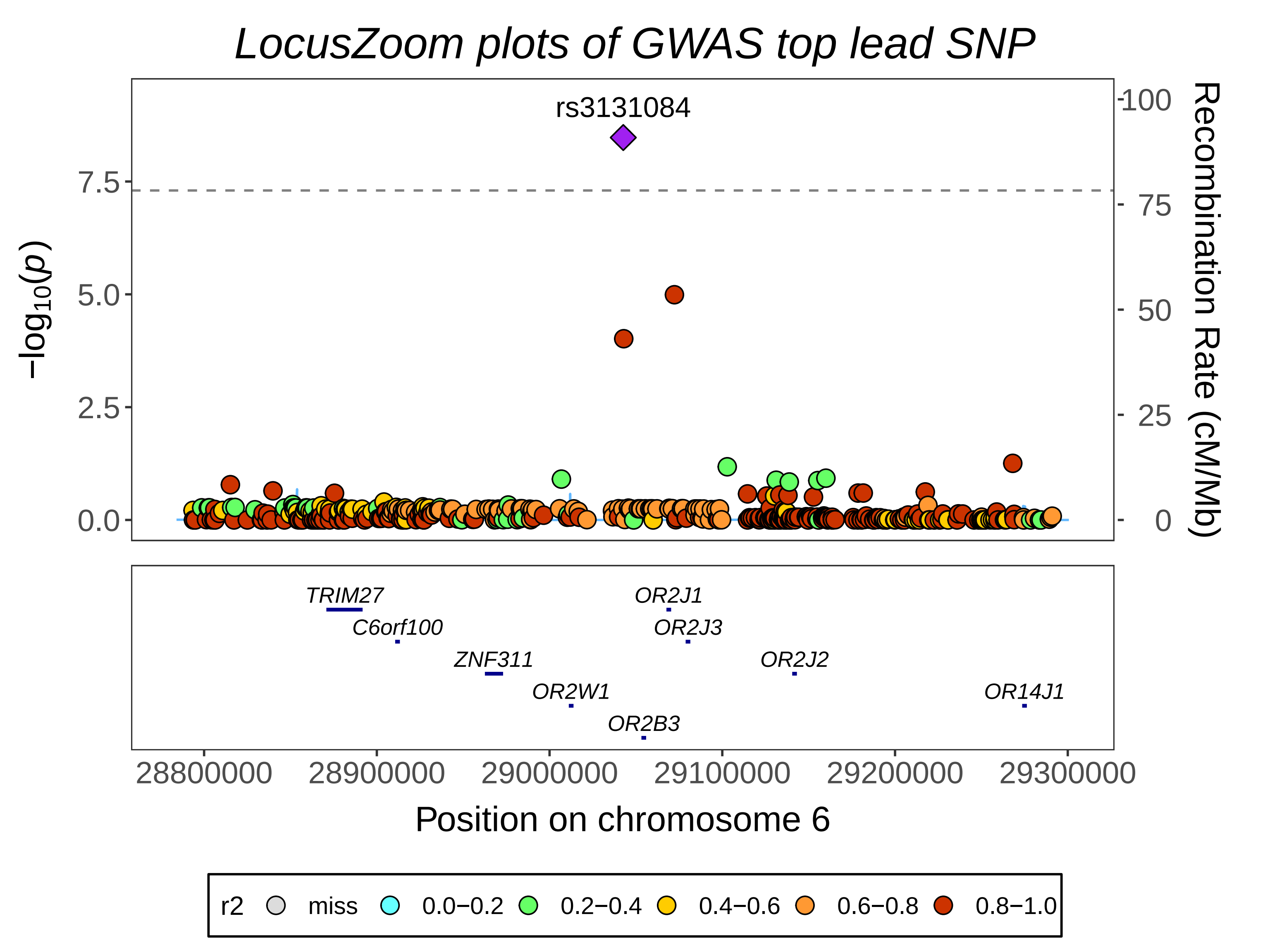
LocusZoom plots of GWAS top lead SNP
rs3131084
−log10(p)
7.5
5.0
2.5
0.0
Recombination Rate (cM/Mb)
100
75
50
25
0
TRIM27
C6orf100
ZNF311
OR2W1
OR2B3
OR2J1
OR2J3
OR2J2
OR14J1
28800000
28900000
29000000
29100000
29200000
29300000
Position on chromosome 6
r2
miss
0.0−0.2
0.2−0.4
0.4−0.6
0.6−0.8
0.8−1.0
