# Supplementary material for: Integrating genomic structural equation modeling and experimental validation to unravel the genetic basis of male genital lichen sclerosus
Source: Front Immunol. 2026 Jul 16;17:1857945. doi: 10.3389/fimmu.2026.1857945 (PMC13435250; doi:10.3389/fimmu.2026.1857945)
Supplement: Supplementary file 3 [file SupplementaryFile2.zip › Sfig_rs11735434_locusZoom.pdf]

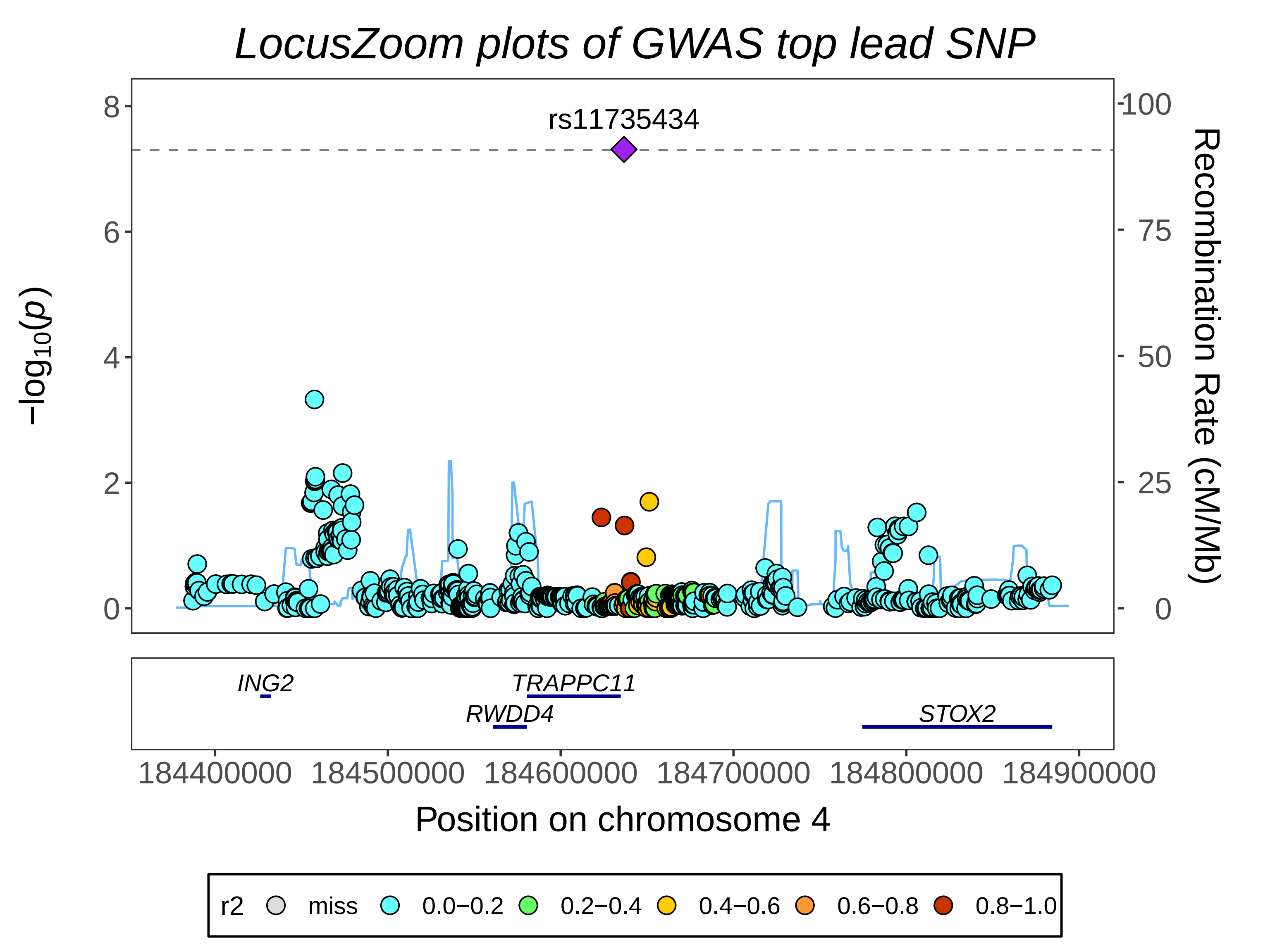
LocusZoom plots of GWAS top lead SNP
rs11735434
−log10(p)
Recombination Rate (cM/Mb)
8
6
4
2
0
100
75
50
25
0
ING2
TRAPPC11
RWDD4
STOX2
184400000
184500000
184600000
184700000
184800000
184900000
Position on chromosome 4
r2
miss
0.0−0.2
0.2−0.4
0.4−0.6
0.6−0.8
0.8−1.0
