# Supplementary material for: Integrating genomic structural equation modeling and experimental validation to unravel the genetic basis of male genital lichen sclerosus
Source: Front Immunol. 2026 Jul 16;17:1857945. doi: 10.3389/fimmu.2026.1857945 (PMC13435250; doi:10.3389/fimmu.2026.1857945)
Supplement: Supplementary file 3 [file SupplementaryFile2.zip › Sfig_rs7678998_locusZoom.pdf]

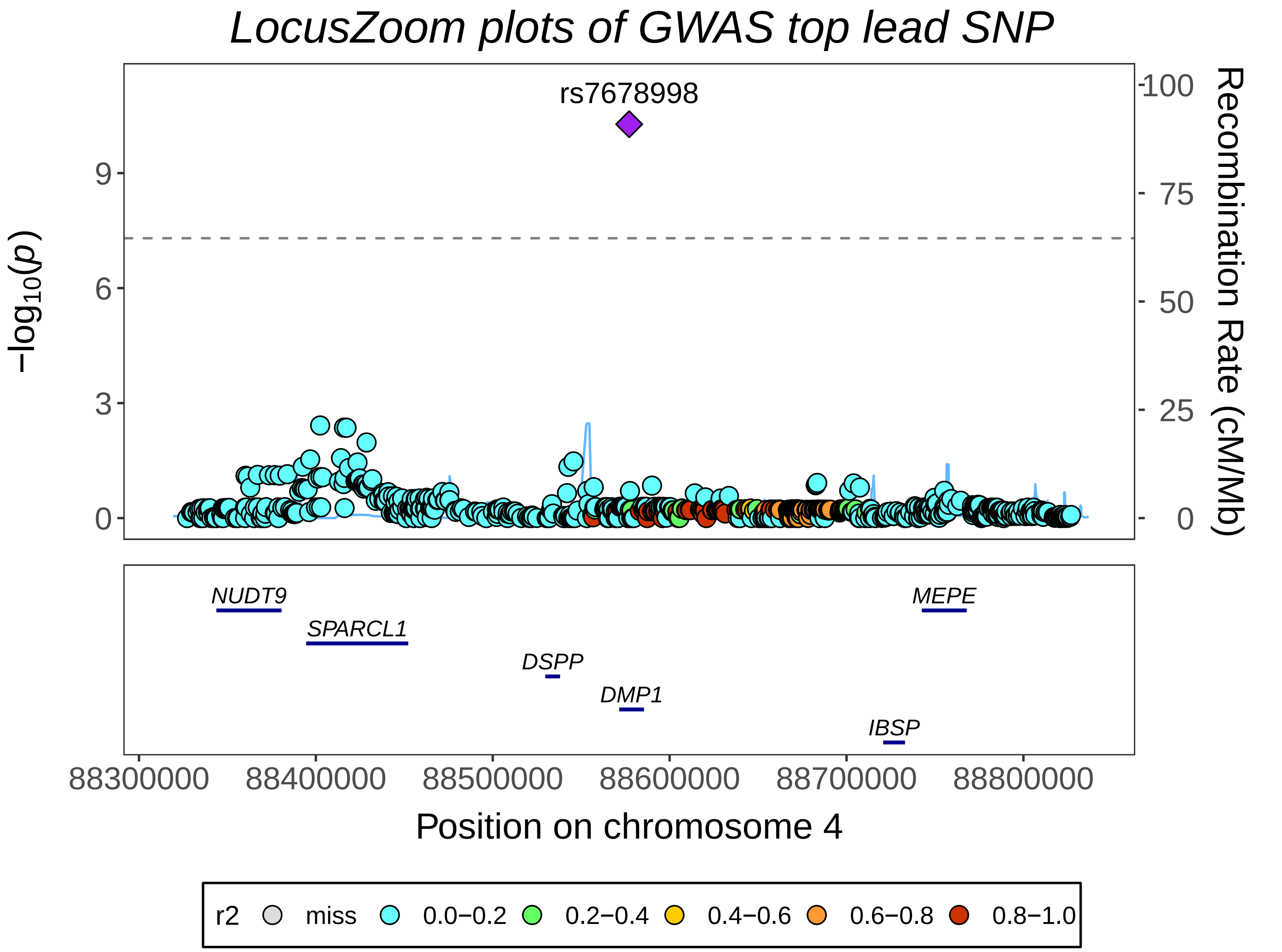
LocusZoom plots of GWAS top lead SNP
rs7678998
−log10(p)
9
6
3
0
Recombination Rate (cM/Mb)
100
75
50
25
0
NUDT9
SPARCL1
DSPP
DMP1
IBSP
MEPE
88300000
88400000
88500000
88600000
88700000
88800000
Position on chromosome 4
r2
miss
0.0−0.2
0.2−0.4
0.4−0.6
0.6−0.8
0.8−1.0
