# Supplementary material for: Integrating genomic structural equation modeling and experimental validation to unravel the genetic basis of male genital lichen sclerosus
Source: Front Immunol. 2026 Jul 16;17:1857945. doi: 10.3389/fimmu.2026.1857945 (PMC13435250; doi:10.3389/fimmu.2026.1857945)
Supplement: Supplementary file 3 [file SupplementaryFile2.zip › Sfig_rs11097189_locusZoom.pdf]

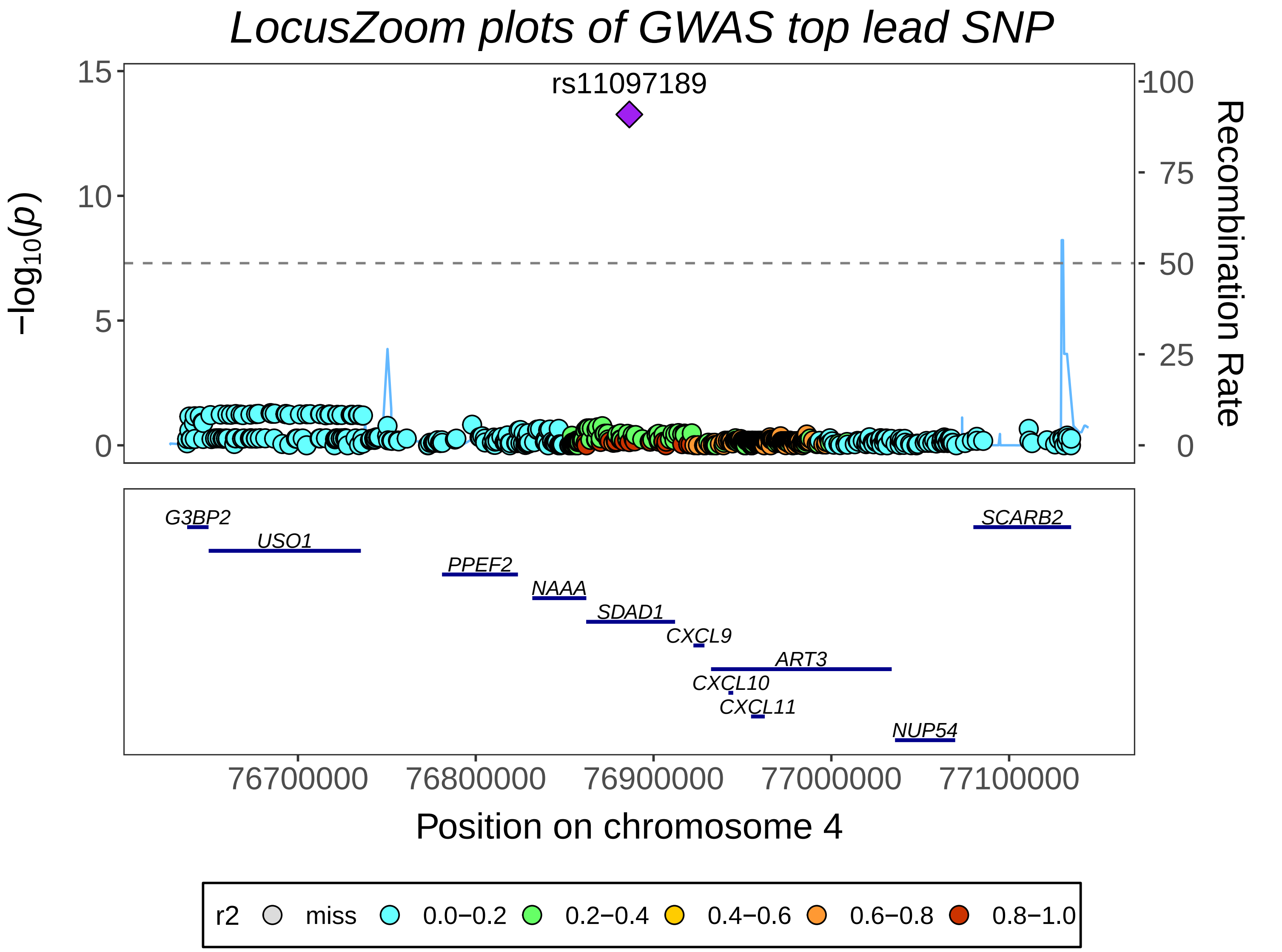
LocusZoom plots of GWAS top lead SNP
rs11097189
−log10(p)
15
10
5
0
Recombination Rate
100
75
50
25
0
G3BP2
USO1
PPEF2
NAAA
SDAD1
CXCL9
ART3
CXCL10
CXCL11
NUP54
SCARB2
76700000
76800000
76900000
77000000
77100000
Position on chromosome 4
r2
miss
0.0−0.2
0.2−0.4
0.4−0.6
0.6−0.8
0.8−1.0
