# Supplementary material for: Integrating genomic structural equation modeling and experimental validation to unravel the genetic basis of male genital lichen sclerosus
Source: Front Immunol. 2026 Jul 16;17:1857945. doi: 10.3389/fimmu.2026.1857945 (PMC13435250; doi:10.3389/fimmu.2026.1857945)

LocusZoom plots of GWAS top lead SNP

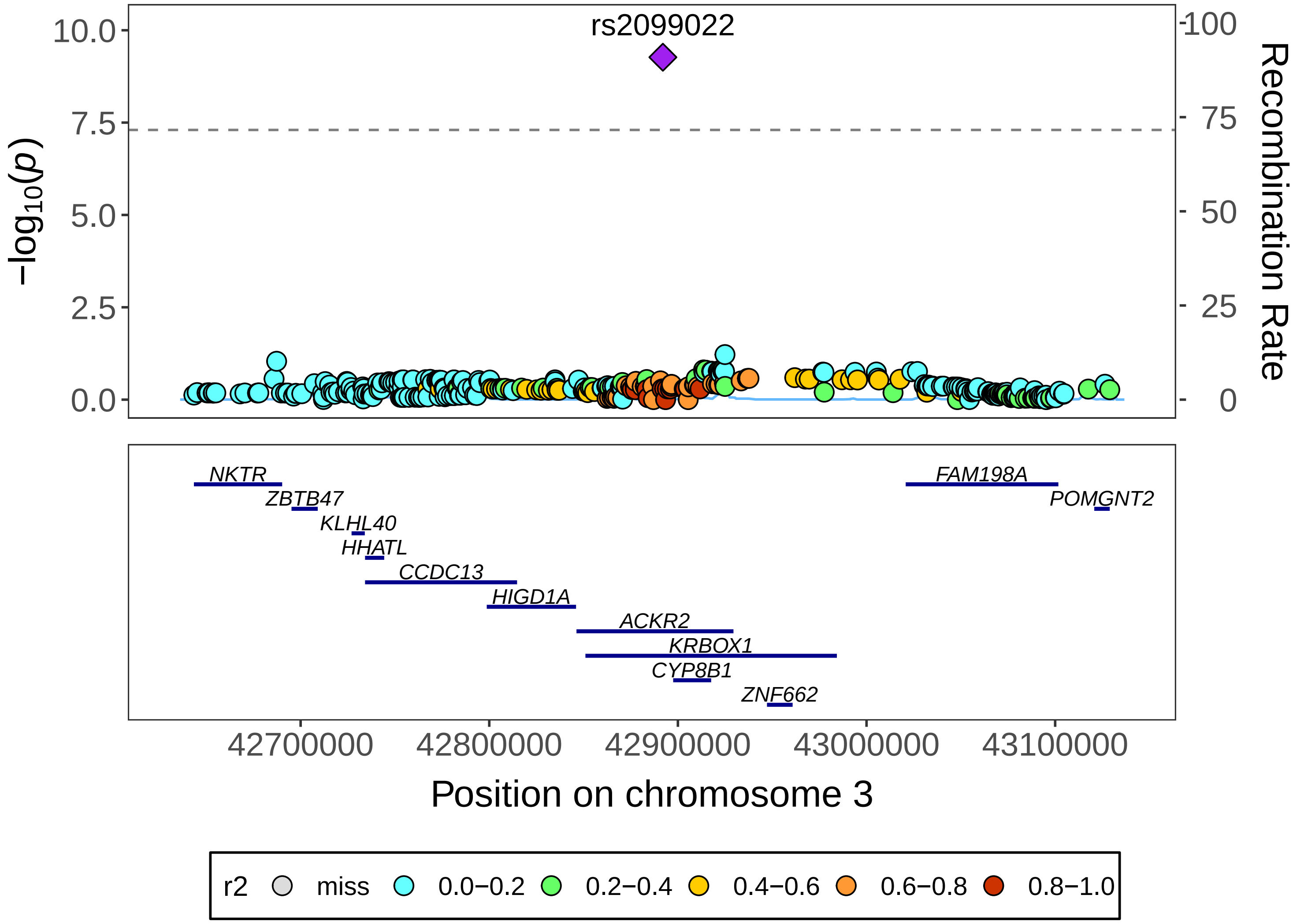

Supplement: Supplementary file 3 [file SupplementaryFile2.zip › Sfig_rs2099022_locusZoom.pdf]
